# Defects in sarcolemma repair and skeletal muscle function after injury in a mouse model of Niemann-Pick type A/B disease

**DOI:** 10.1186/s13395-018-0187-5

**Published:** 2019-01-05

**Authors:** V. Michailowsky, H. Li, B. Mittra, S. R. Iyer, D. A. G. Mazála, M. Corrotte, Y. Wang, E. R. Chin, R. M. Lovering, N. W. Andrews

**Affiliations:** 1 Department of Cell Biology and Molecular Genetics, University of Maryland, 2134 Bioscience Research Building, College Park, MD 20742-5815 USA; 2 Department of Kinesiology, University of Maryland School of Public Health, College Park, MD USA; 3 Center for Genetic Medicine Research, Children’s National Health System, Washington DC, USA; 4 Cytokinetics Inc., South San Francisco, CA USA; 5 Department of Orthopaedics, University of Maryland School of Medicine, Baltimore, MD USA; 6 Proteomics Core Facility, College of Computer, Mathematical and Natural Sciences, University of Maryland, College Park, MD USA

**Keywords:** Acid sphingomyelinase, Skeletal muscle, Lysosome, Calcium, Plasma membrane repair

## Abstract

**Background:**

Niemann-Pick disease type A (NPDA), a disease caused by mutations in acid sphingomyelinase (ASM), involves severe neurodegeneration and early death. Intracellular lipid accumulation and plasma membrane alterations are implicated in the pathology. ASM is also linked to the mechanism of plasma membrane repair, so we investigated the impact of ASM deficiency in skeletal muscle, a tissue that undergoes frequent cycles of injury and repair in vivo.

**Methods:**

Utilizing the NPDA/B mouse model ASM^−/−^ and wild type (WT) littermates, we performed excitation-contraction coupling/Ca^2+^ mobilization and sarcolemma injury/repair assays with isolated *flexor digitorum brevis* fibers, proteomic analyses with *quadriceps femoris*, flexor digitorum brevis, and *tibialis posterior* muscle and in vivo tests of the contractile force (maximal isometric torque) of the quadriceps femoris muscle before and after eccentric contraction-induced muscle injury.

**Results:**

ASM^−/−^ flexor digitorum brevis fibers showed impaired excitation-contraction coupling compared to WT, a defect expressed as reduced tetanic [Ca^2+^]_i_ in response to electrical stimulation and early failure in sustaining [Ca^2+^]_i_ during repeated tetanic contractions. When injured mechanically by needle passage, ASM^−/−^ flexor digitorum brevis fibers showed susceptibility to injury similar to WT, but a reduced ability to reseal the sarcolemma. Proteomic analyses revealed changes in a small group of skeletal muscle proteins as a consequence of ASM deficiency, with downregulation of calsequestrin occurring in the three different muscles analyzed. In vivo, the loss in maximal isometric torque of WT quadriceps femoris was similar immediately after and 2 min after injury. The loss in ASM^−/−^ mice immediately after injury was similar to WT, but was markedly larger at 2 min after injury.

**Conclusions:**

Skeletal muscle fibers from ASM^−/−^ mice have an impairment in intracellular Ca^2+^ handling that results in reduced Ca^2+^ mobilization and a more rapid decline in peak Ca^2+^ transients during repeated contraction-relaxation cycles. Isolated fibers show reduced ability to repair damage to the sarcolemma, and this is associated with an exaggerated deficit in force during recovery from an in vivo eccentric contraction-induced muscle injury. Our findings uncover the possibility that skeletal muscle functional defects may play a role in the pathology of NPDA/B disease.

**Electronic supplementary material:**

The online version of this article (10.1186/s13395-018-0187-5) contains supplementary material, which is available to authorized users.

## Background

Niemann-Pick disease types A and B (NPDA/B) are diseases caused by recessive mutations in *SMPD1*, the gene encoding the lysosomal enzyme acid sphingomyelinase (ASM). NPDA, the most serious form of the disease, is characterized by very low ASM activity (< 5% of normal levels), extensive neurodegeneration, and early death at around 2 years of age. NPDB, on the other hand, presents a phenotypic heterogeneity that is attributed to partial ASM deficiency (5–10% of normal levels) [1, 2]. The birth rate of NPDA children in the Ashkenazi Jewish community is estimated at approximately 1:40,000. In this population, three mutations account for > 90% of infants diagnosed with NPDA, and DNA-based screening of Ashkenazi adults estimate the carrier frequency for these mutations at 1:80–1:100 [1]. Thus, NPDA and NPDB represent very serious health concerns in certain human populations.

ASM hydrolyses the head group of sphingomyelin, generating phosphorylcholine and ceramide. NPDA and NPDB have characteristics of lysosomal storage diseases, because intra-lysosomal accumulation of lipids resulting from ASM deficiency is thought to lead to cellular dysfunction and death, particularly in neurons. However, during the last decade, it has become apparent that ASM also has an extracellular role, remodeling lipid microdomains on the outer leaflet of the plasma membrane (PM) after being secreted during conditions of cellular stress [3–7]. These findings have led to the suggestion that the clinical findings in NPDA/B might also be due to PM abnormalities [7]. In particular, recent studies uncovered a link between the extracellular release of ASM from lysosomes and the mechanism by which cells repair wounds to their PM [8, 9].

Injury and repair of the PM is a normal physiological process that occurs in many cell types [10–13] and is particularly frequent in tissues under mechanical stress such as the skeletal muscle [14–16]. A major advance in the understanding of PM repair came from the realization that lysosomes, in addition to their role as intracellular degradative organelles, respond to the influx of Ca^2+^ through PM wounds by fusing with the PM [17, 18]. Ca^2+^-triggered exocytosis of lysosomes is required for the repair of PM lesions in several cell types [9, 11, 12, 19], through a process that was initially thought to involve a membranous “repair patch” [20]. However, recent studies revealed that the extracellular release of ASM from lysosomes after wounding has a direct role in PM resealing by promoting lesion removal through endocytosis [21, 22]. Thus, impaired PM repair after injury may be an important but so far overlooked component of the pathophysiology of NPDA/B. In this study, we investigated this hypothesis by focusing on the skeletal muscle, a tissue that is frequently injured in vivo [14, 16], using an ASM knockout mouse model that shows clinical, biochemical, and pathological abnormalities that mimic the characteristics of human NPDA/B [23, 24]. These ASM^−/−^ mice develop ataxia and tremors at approximately 8 weeks after birth, followed by a rapidly progressing neurodegeneration that culminates in unresponsiveness, lethargy, and death by 6–8 months of age. Prior to this study, however, no information was available on the skeletal muscle function of these animals.

## Methods

### Animal handling and *flexor digitorum brevis* (FDB) muscle fiber isolation

ASM^+/−^ mice (generated by E. Schuchman and provided by S. Muro, University of Maryland) were bred to generate ASM^−/−^ and ASM^+/+^ (WT) littermates. All protocols for animal handling were approved by the University of Maryland’s Institutional Animal Care and Use Committee (IACUC). The University of Maryland at College Park is an AAALAC-accredited institution. At about 8 weeks of age, gender-matched mice (male or female animals were used in most assays with no differences observed) were euthanized, and the FDB muscle was excised and digested with 0.2% type 2 collagenase/minimal essential media (MEM)/10% fetal bovine serum (FBS) solution at 37 °C in a 5% CO_2_ atmosphere for 4 h to obtain FDB single muscle fibers [25, 26].

### FDB mechanical wounding

After collagenase digestion, FDB fibers were gently dissociated by several passages in a Pasteur pipette and washed twice in DMEM without Ca^2+^ using spontaneous sedimentation (1 g) for 15 min at room temperature followed by removal of the supernatant. The fibers were then resuspended in 1.2 ml DMEM without Ca^2+^ + 10 mM EGTA, and aliquots of 200 μl were transferred to six tubes and again allowed to sediment. The supernatant was removed, the fibers were resuspended in 1 ml DMEM with Ca^2+^ (condition permissive for sarcolemma repair) or 1 ml DMEM without Ca^2+^ (condition not permissive for sarcolemma repair) and allowed to sediment for 15 min on ice. The sedimented fibers were then passed through a 30-gauge needle using a 1-ml syringe (pulling the plunger up and down once), incubated at 37 °C for 5 min, followed by addition of propidium iodide (PI) (1:50 dilution of 5 mg/ml solution). After 5 min on ice, 1 ml of DMEM without Ca^2+^ 10 mM EGTA was added and the fibers were allowed to sediment for 15 min on ice. After removing the supernatant, the fibers in each tube were resuspended in 500 μl PBS 4% paraformaldehyde (PFA) and left at room temperature for 15 min, centrifuged at 21×*g* for 5 min and resuspended in 0.25 ml PBS and imaged in DeltaVision deconvolution microscope using a × 10 objective. PI staining levels in fibers were determined by fluorescence intensity measurements using Volocity software, on > 200 fibers for each experimental condition. Hyper-contracted fibers, which were PI-positive under all conditions, were excluded from the analysis.

### Electron microscopy

Isolated FDB fibers were injured by passage through a 30-gauge needle as described above. Fibers were then immediately incubated at 37 °C for 1 min to induce the sarcolemma repair reaction, before being placed in an ice-cold bath to stop the process. Non-wounded control fibers were also placed on ice. All fibers were then allowed to sediment for 15 min on ice before being washed in ice-cold PBS, sedimented again, and finally resuspended in ice-cold 2% glutaraldehyde in 0.1 M cacodylate fixative and placed at room temperature for 1 h before being processed for transmission electron microscopy (TEM) and imaged in a Zeiss EM10CA electron microscope, as previously described [25].

### Intracellular Ca^2+^ measurements

Fura-2 AM was used to assess changes in intracellular Ca^2+^ levels in single muscle fibers, as previously described [26]. Briefly, after single muscle fibers were isolated from FDB muscle, the fibers were loaded with Fura-2 AM for 15 min, which allows the fluorescent dye sufficient time to diffuse into the myoplasm. Fura-2 emits a signal when excited at 380 nm (unbound state) or at 340 nm (bound to Ca^2+^), and the ratio at 340 nm/380 nm reflects the relative intracellular Ca^2+^ concentration ([Ca^2+^]_i_). The loaded fibers were then washed of excess dye and placed in a stimulation chamber containing parallel electrodes. The stimulation chamber was placed on top of a Nikon TiU microscope, and the IonOptix Hyperswitch system was used to assess the Fura-2 fluorescence ratio.

### Single muscle fiber stimulation protocol

For the assessment of muscle fiber [Ca^2+^]_i_, fibers were perfused with a stimulation solution comprised of 121 mM NaCl, 5 mM KCl, 0.5 mM MgCl_2_, 0.4 mM NaH_2_PO_4_, 24 mM NaHCO3, 5.5 mM glucose, and 1.8 mM CaCl_2_ [27]. Fetal bovine serum (FBS) (0.2%) was added to the stimulation solution, which was continuously aerated with 95% O_2_/5% CO_2_ and maintained at pH 7.3 [27]. Intracellular [Ca^2+^] was measured in single muscle fibers across a range of pulse frequencies (10, 30, 50, 70, 100, 120, and 150 Hz) to establish a Ca^2+^-frequency relationship. The 10–150 Hz pulses were delivered as 350 ms pulse trains with a 1-min interval between frequencies (i.e., seven stimulation frequencies assessed over 7 min) to minimize the potential for fatigue. Following the Ca^2+^-frequency assessment, muscle fibers were allowed to recover for 5 min before the repeated tetanic contraction (RTC) protocol.

The RTC protocol consisted of intermittent 100 Hz tetani (350 ms duration), with the duration of the rest periods between tetanic contractions reduced every 2 min, as previously described [27]. The fibers underwent one contraction every 4 s for 2 min, then one contraction every 3 s for 2 min, then one contraction every 2 s for 2 min, and finally one contraction every second, until the peak Fura-2 ratio was reduced to 50% of the initial ratio [27]. This 50% decrease in peak Ca^2+^ transient was previously shown to correspond to a 70% decrease in force in isolated fibers exposed to RTC and defined as the point of fatigue [28]. Following the RTC, the Ca^2+^-frequency relationship was reassessed 30 and 60 min after the last 100 Hz tetanus to determine the frequency-dependent recovery of [Ca^2+^]_i_ and E-C coupling from repetitive contraction-relaxation cycles. A two-way analysis of variance (ANOVA) of Fura-2 ratios at each stimulation frequency was performed to analyze the results, with statistical significance set at an *α* level of 0.05.

### SERCA1 and calsequestrin protein expression

Total protein was isolated from FDB by homogenization in lysis buffer (20 mM HEPES buffer, pH 7.5, 100 mM NaCl, 1.5 mM MgCl_2_, 0.1% Triton X-100, 20% glycerol) containing 1 mM DTT and a protease inhibitor cocktail (Sigma) on ice. Samples were centrifuged at 20,000 *g* at 4 °C, and the supernatant was collected and frozen at − 80 °C until analyzed. Total protein concentration was determined using a BCA assay (Thermo Fisher Scientific). Samples were solubilized in SDS loading buffer and denatured by heating at 100 °C for 5 min. For immunoblotting, 20–30 μg total protein was loaded on bis-acrylamide gels, separated by SDS-PAGE electrophoresis, and transferred to PVDF membranes (EMD Millipore, Billerica, MA, USA). Membranes were blocked with 5% nonfat milk at room temperature for 1 h followed by overnight incubation at 4 °C in anti-SERCA1 (Thermo Fisher Scientific) or anti-calsequestrin polyclonal antibodies (Abcam ab3516). Antibodies against α-tubulin or actin (Abcam) were used as loading controls. Antibody-reactive proteins were detected with Clarity western ECL substrate (Bio-Rad, Hercules, CA, USA), imaged using an Image Lab system (Bio-Rad), and quantified by densitometry.

### Proteomics analysis

Independently isolated (three biological replicates) *quadriceps femoris* (QF), *tibialis posterior* (TP), and FDB muscles from WT or ASM^−/−^ mice were lysed in 0.1 M Tris-HCL, pH 7.4, 0.1 M DTT and 4% SDS and incubated at 95 °C for 5 min, followed by homogenization in a Tissue-Tearer (Model 985370, Biospec Products). After sonication (Branson), the lysates were centrifuged at 16,000×*g* for 10 min, and samples of the supernatant containing about 25 μg protein were reduced, alkylated, and digested within S-Trap micro spin columns (Protifi) following the manufacturer’s recommendations. In short, homogenates containing 2% SDS were reduced with DTT and cysteine-alkylated with iodoacetamide. The protein solution was then acidified with phosphoric acid, diluted with 90% MeOH, and loaded into S-Trap micro columns. The captured protein was washed with 90% MeOH to remove SDS and trypsin was added at 1:25 *w*/*w* ratio followed by incubation at 47 °C for 1 h. Tryptic peptides were sequentially eluted with 50 mM triethylamine bicarbonate, 0.2% formic acid, and 50% acetonitrile containing 0.2% formic acid. Eluents were combined, and the solvent removed by centrifugal evaporation.

Nano LC-MS/MS analysis was carried out with a Thermo Scientific Fusion Lumos tribrid mass spectrometer interfaced to a UltiMate3000 RSLCnano HPLC system. One microgram of the tryptic digest was loaded and desalted in an Agilent Zorbax 300 SB-C18 trapping column (0.3 × 5 mm) at 5 μl/min for 5 min. Peptides were then eluted into a 75 μm × 250 mm Thermo Scientific Accalaim PepMap 100 column (3 μm, 100 Å) and chromatographically separated using a binary solvent system consisting of A: 0.1% formic acid and 2.5% acetonitrile and B: 0.1% formic acid and 75% acetonitrile, at a flow rate of 300 nl/min. A gradient was run from 1% B to 42% B over 150 min, followed by a 5-min wash step with 99% B and a 10-min equilibration at 1% B before the next sample was injected. Precursor masses were detected in the Orbitrap at *R* = 120,000 (m/z 200). Fragment masses were detected in the linear ion trap at unit mass resolution. Data-dependent MS/MS was carried out with top of speed setting, cycle time 3 s with dynamic exclusion of 30 s.

Protein identification and relative quantification were carried out using the Proteome Discoverer software package (Thermo Scientific). Raw data was searched against a *Mus musculus* proteome database from Uniprot (2016-05-10) along with a contaminant protein database with both Sequest HT and Mascot search engines. Proteins annotated in Uniprot and/or reported in the literature as functionally important in the skeletal muscle [29] were extracted from the Uniprot database and used to generate a subset skeletal muscle database to assist further analysis. Cysteine carbomidomethylation was set as a fixed modification. Methionine oxidation, asparagine, and glutamine deamidation were set as variable modifications. Peptide mass tolerance was ± 20 ppm for search and later filtered to 5 ppm in consensus report; fragment mass tolerance was ± 0.8 Da. Only proteins with at least two peptides identified were considered for further analysis. For relative quantification, label-free quantification (LFQ) was carried out using the MINORA feature detection followed by precursor intensity quantification of unique and razor peptides. Peptides with variable modifications were excluded from quantification. A protein was not quantified if only detected in one replicate. Normalization between samples was carried out based on total peptide amount. Changes above twofold between WT and ASM^−/−^ samples from the same muscle were considered significant (ANOVA).

### In vivo injury and measurement of isometric torque

In vivo testing of muscle force and susceptibility to injury was performed as previously described [30–33]. Briefly, WT and ASM^−/−^ mice 6–7 weeks old were anesthetized by inhalation anesthesia and placed in a supine position. The animal’s thigh was stabilized and the ankle secured to a lever arm. The axis of the knee was aligned with the axis of a stepper motor, which was attached to a torque sensor. The femoral nerve was stimulated with subcutaneous needle electrodes to activate the quadriceps muscle. Contractile activation, onset of forced knee flexion, and torque data collection were synchronized with the use of custom software. In this system, a reproducible QF injury is produced through forced lengthening superimposed onto maximal isometric contractions, through a 60 deg arc of motion at an angular velocity of 900°, beginning 200 ms after tetanic stimulation. Maximal isometric torque was measured before injury and at selected time points after injury (0 and 2 min). After injury, the animals were removed from the apparatus and returned to the cage for monitoring. For each genotype, seven female mice were analyzed. Controls included sham procedures (contractions without lengthening or passive lengthening without contractions, both with the thigh immobilized) [34].

## Results

### ASM^−/−^ muscle fibers show altered intracellular Ca^2+^ handling in an excitation-contraction coupling assay

**Fig. 1:**
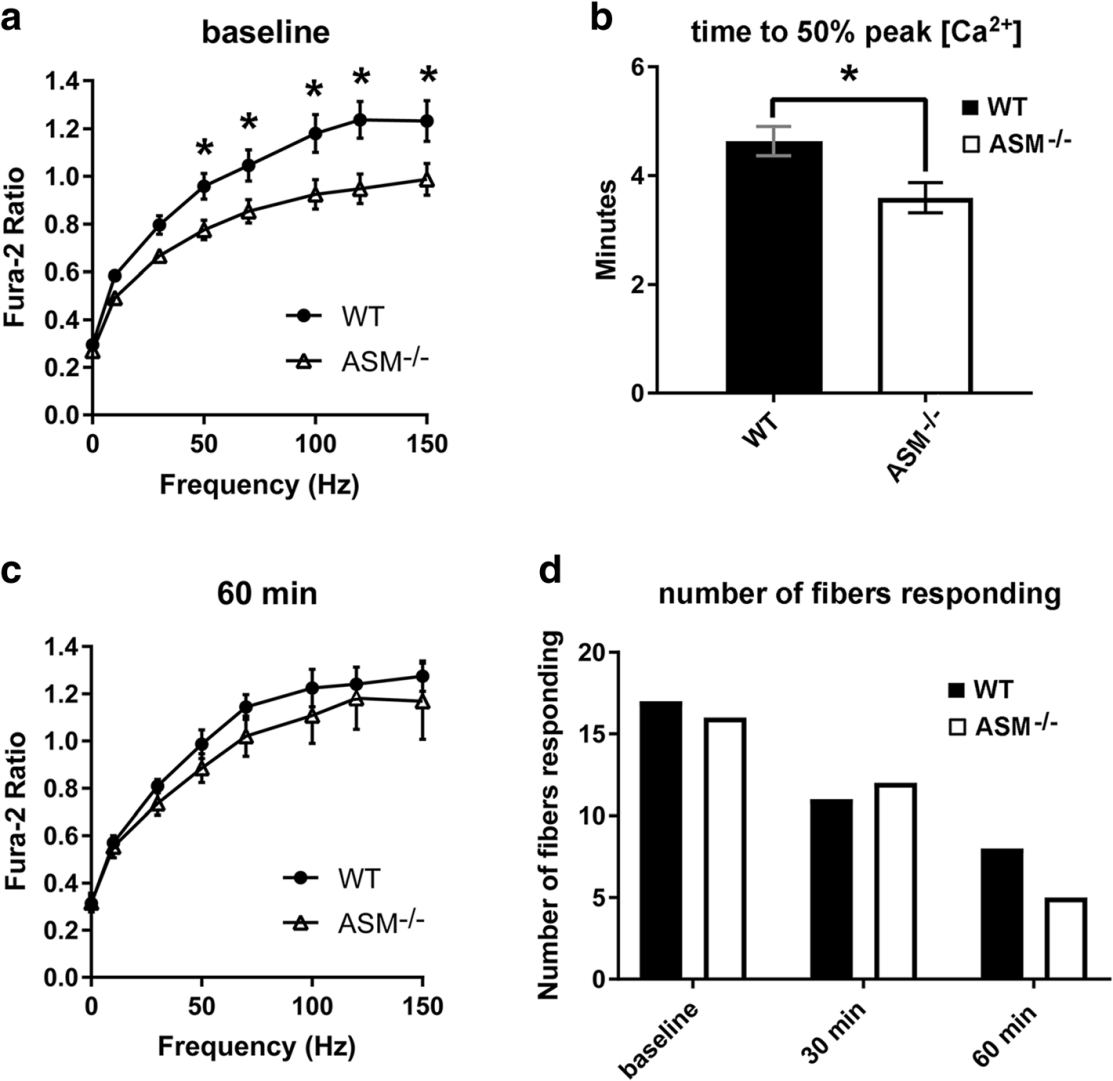
ASM^−/−^ FDB fibers show defects in excitation-contraction coupling and recovery from repeated tetanic contractions. FDB fibers were isolated from six ASM-deficient mice (ASM^−/−^) and six wild type littermates (WT) and subjected to electrical field stimulation to activate Ca^2+^ release and muscle fiber contraction. **a** Peak Fura-2 ratios of single FDB fibers at baseline in response to stimulation across a range of physiological activation frequencies. ASM^−/−^ fibers showed significantly lower peak Fura-2 ratios than WT fibers at baseline. *p = 0.020 at 70 Hz, *p* = 0.001 at 100 Hz, *p* = 0.001 at 120 Hz, *p* = 0.008 at 150 Hz. **b** Average time to 50% of initial peak Fura-2 ratio during repeated 100 Hz tetanic contractions. Significantly lower values were observed in ASM^−/−^ fibers when compared to WT. *p = 0.011. **c** Peak Fura-2 ratios of single FDB fibers during measurements after 60 min of recovery from repeated tetanic contractions. No significant differences were observed at 60 min recovery in peak Fura-2 ratios between ASM^−/−^ and WT fibers. **d** Number of fibers responding to electrical stimuli at baseline and at 30 or 60 min of recovery from repeated tetanic contractions. No significant differences were observed in the total number of responsive fibers between WT or ASM^−/−^

Flexor digitorum brevis (FDB) muscle fibers can be efficiently isolated from mice by dissection and collagenase digestion and, for this reason, have been extensively used to study fundamental aspects of muscle function at the single fiber level. To obtain an initial assessment of the functional impact of ASM deficiency on isolated FDB fibers, we utilized a Ca^2+^ mobilization assay, previously used to characterize excitation-coupling (E-C) defects in various mouse models of muscle disease [26]. Fura-2 was used to detect changes in intracellular Ca^2+^ in response to electrical stimulation of FDB muscle fibers at physiologically relevant stimulation frequencies (i.e., 10–150 Hz). There was a significant reduction in peak Fura-2 ratio at the higher stimulation frequencies in ASM^−/−^ fibers compared to WT fibers (Fig. 1a). Specifically, the peak Fura-2 ratios of ASM^−/−^ fibers were significantly lower compared to WT fibers at 50 Hz (0.959 ± 0.053 vs. 0.775 ± 0.041), 70 Hz (0.853 ± 0.050 vs. 1.046 ± 0.065), 100 Hz (0.924 ± 0.063 vs.1.180 ± 0.080), 120 Hz (0.948 ± 0.062 vs. 1.237 ± 0.077), and 150 Hz (0.988 ± 0.067 vs. 1.233 ± 0.085). These data indicated that peak intracellular Ca^2+^ in response to muscle depolarization was impaired and suggested alterations in excitation-contraction coupling and/or impaired Ca^2+^ mobilization. The observed differences at the highest stimulation frequencies further suggested a deficit in stored intracellular Ca^2+^.

After monitoring the response of FDB fibers to single stimulations across a range of frequencies, the Fura-2 response to repeated tetanic contractions at a high frequency (100 Hz) was assessed in order to determine (i) the ability to sustain repeated contractions and (ii) the ability to recover from a bout of repeated tetanic contractions. The latter was previously shown to be impaired in other muscle disorders [35], as it challenges the ability of muscle fibers to sustain cyclical periods of Ca^2+^ release and uptake. Repeated tetanic stimulation was applied until the peak Fura-2 ratio was reduced to 50% of its initial value [27]. This 50% reduction in peak Ca^2+^ transient level was previously shown to correspond to a 70% decrease in force in isolated fibers subjected to repeated contractions and was defined as the point of fatigue [28]. Recovery was assessed 30 and 60 min after the fibers reached this fatigue point. In response to repeated tetanic contractions, peak Fura-2 was reduced to 50% of the initial levels in WT fibers by 4.64 ± 0.27 min. In ASM^−/−^ FDB fibers, the average time to reach 50% of peak Fura-2 was 3.53 ± 0.26 min, which was significantly different from WT fibers (Fig. 1b). Thus, ASM^−/−^ FDB fibers were not able to sustain intracellular peak Ca^2+^ levels in response to repeated 100 Hz tetanic contractions and thus appeared to reach a point of fatigue faster than WT FDB fibers.

In WT fibers, there were no significant differences between peak Fura-2 ratios at baseline and after 60 min of recovery from repeated tetanic contractions. Interestingly, ASM^−/−^ fibers showed significantly higher peak Fura-2 levels after 60 min of recovery from repeated contractions. Specifically, the Fura-2 ratios of ASM^−/−^ fibers were significantly higher at 60 min after repeated tetanic contractions when compared to baseline at 70 Hz (1.021 ± 0.085 vs. 0.853 ± 0.050) and 120 Hz (1.182 ± 0.133 vs. 0.948 ± 0.062), and trended towards significance at rest (0.317 ± 0.040 vs. 0.269 ± 0.009; *p* = 0.065), 10 Hz (0.553 ± 0.047 vs. 0.492 ± 0.054; *p* = 0.055), 50 Hz (0.885 ± 0.061 vs. 0.775 ± 0.041; *p* = 0.076), and 100 Hz (1.108 ± 0.119 vs. 0.924 ± 0.063; *p* = 0.079) (compare Fig. 1a with Fig. 1c). Thus, despite the reductions in peak Fura-2 ratio in ASM^−/−^ compared to WT fibers observed at baseline (Fig. 1a), we found no significant differences in peak Fura-2 ratio after 60 min of recovery. Furthermore, from a total of 17 WT fibers that responded with an elevation of [Ca^2+^]_i_ at baseline, 11 (64%) also responded to all stimulation frequencies after 30 min of recovery from repeated tetanic contractions, and this number decreased to 8 (47%) after 60 min of recovery. From 16 ASM^−/−^ fibers, 12 (75%) responded after a 30-min recovery, and this number decreased to 5 (31%) after a 60-min recovery from repeated tetanic contractions (Fig. 1d). Thus, a similar number of fibers from both groups responded at 30-min (results not shown) and 60-min post-RTC (Fig. 1d) after repeated tetanic contractions.

These data indicate that FDB fibers from ASM^−/−^ mice are impaired in E-C coupling when compared to WT, resulting in reduced Ca^2+^ release from the sarcoplasmic reticulum (SR). ASM^−/−^ fibers reach a failure point for maintaining [Ca^2+^]_i_ (and thus would fatigue) sooner during repeated bouts of activation and, surprisingly, also show an enhanced peak tetanic Ca^2+^ transient following repetitive activation. These data suggest alterations in intracellular Ca^2+^ handling and storage in the absence of ASM expression.

### WT and ASM^−/−^ muscle fibers express similar levels of most proteins that regulate muscle function

**Fig. 2:**
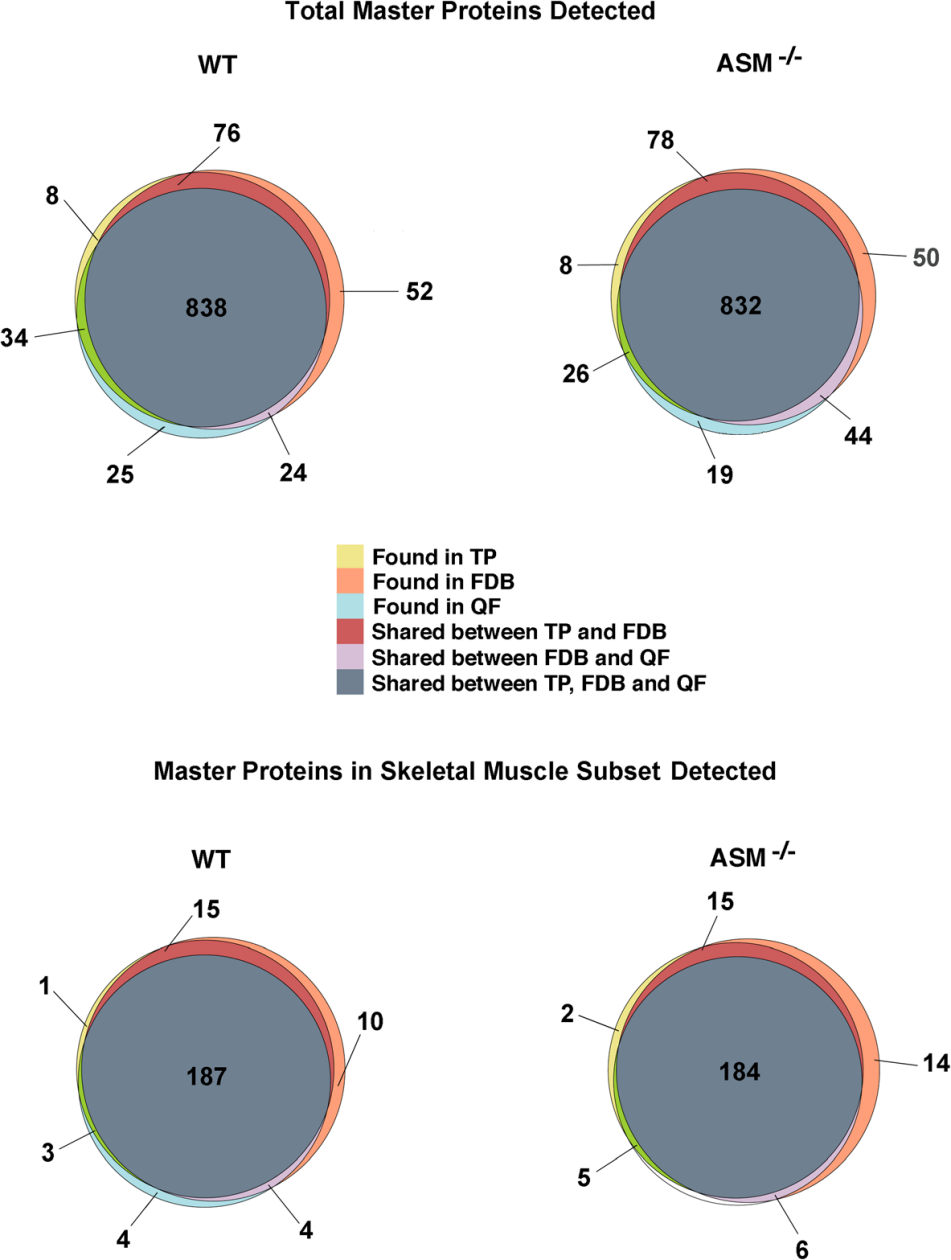
Venn diagrams of the overlap between master proteins detected in TP, FDB, and QF muscle fibers. Top diagrams, overlap between the three muscles of all master proteins detected for WT or ASM^−/−^; bottom diagrams, overlap between the three muscles of proteins from a skeletal muscle subset database assembled using the skeletal muscle annotation in Uniprot (https://www.uniprot.org/), supplemented with functionally important skeletal muscle proteins curated from the literature [29]

**Fig. 3:**
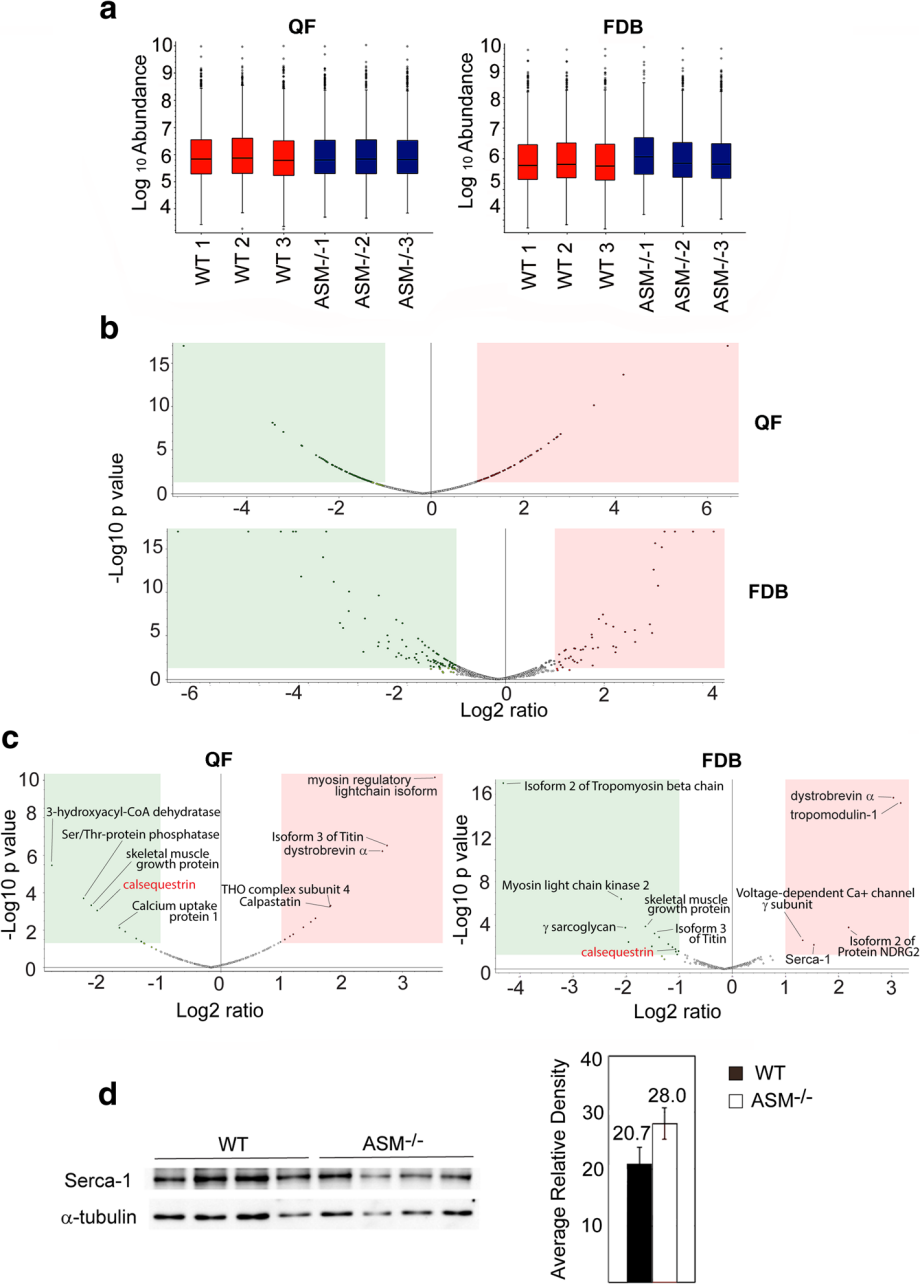
Comparative proteomic analysis of QF and FDB muscle fibers from WT and ASM^−/−^ mice. **a** Protein abundance values for three biological replicates of WT or ASM^−/−^ QF and FDB muscles isolated from WT and ASM^−/−^ mice. **b** Volcano plots indicating statistically significant (*P* < 0.05) differences between WT and ASM^−/−^ samples in the expression of all master proteins identified for QF and FDB muscles. Green box, proteins downregulated more than twofold in ASM^−/−^ relative to WT; pink box, proteins upregulated more than twofold in ASM^−/−^ relative to WT. **c** Volcano plots indicating statistically significant (*P* < 0.05) differences between WT and ASM^−/−^ samples in the expression of master proteins within a subset of functionally important skeletal muscle proteins, in QF and FDB muscles. Green box, proteins downregulated more than twofold in ASM^−/−^ relative to WT; pink box, proteins upregulated more than twofold in ASM^−/−^ relative to WT. **d** FDB fibers isolated from four WT mice and four ASM^−/−^ mice were solubilized and analyzed by Western blot with anti-SERCA1 antibodies (each lane corresponds to fibers isolated from one animal). Antibodies against α-tubulin were used as a loading control. **e** Densitometry values of the data in **d** expressed as SERCA1/α-tubulin ratio, showing no significant differences between the amount of SERCA1 in WT and ASM^−/−^ FDB fibers.

The reduced peak [Ca^2+^]_i_ in response to stimulation of ASM^−/−^ FDB fibers when compared to WT fibers (Fig. 1a) raised the possibility that ASM^−/−^ and WT FDB fibers might express different levels of proteins involved in regulating cytosolic Ca^2+^ levels. To address this possibility, we performed state-of-the-art quantitative proteomic analyses of three skeletal muscles in WT and ASM^−/−^ mice: FDB, quadriceps femoris QF, and TP. Protein profiling by nanoscale liquid chromatography coupled to tandem mass spectrometry (nano LC-MS/MS) and Uniprot database search identified 982 protein groups (herein referred to as master proteins) in QF, 1017 in FDB, and 944 in TP from WT animals and 972 master proteins in QF, 1037 in FDB, and 948 in TP from ASM^−/−^ animals, with an extensive overlap between the three different muscles (Fig. 2). Label-free quantification (LFQ) showed that protein abundance in the three muscles was consistent among all three biological replicates for both WT and ASM^−/−^ (Fig. 3a, Additional file 1: Figure S1A), with > 80% overlap between the three muscles analyzed. By comparing the relative abundance of all protein groups detected in each muscle for both genotypes, we found that significant changes of more than twofold in master proteins were 6.1% (downregulated) and 4.7% (upregulated) in QF, 8% (downregulated) and 3% (upregulated) in FDB, and 5.3% (downregulated) and 4.2% (upregulated) in TP ASM^−/−^ mice, when compared to WT (Fig. 3b, Additional file 1: Figure S1B, Additional file 2: Table S1, Additional file 3: Table S2, Additional file 4: Table S3). We also compared WT and ASM^−/−^ samples for the relative abundance of proteins in a subset database assembled using the skeletal muscle annotation in Uniprot (https://www.uniprot.org/), supplemented with functionally important skeletal muscle proteins curated from the literature [29] (Fig. 2, Additional file 5: Table S4). Of the 227 master proteins in this mouse skeletal muscle protein subset database, 207 were detected in QF, 217-220 in FDB, and 196-199 in TP, with extensive overlap (~ 80%) among the three muscles, for both mouse genotypes (Fig. 2, Additional file 5: Table S4). Thus, despite the variations in fiber type composition among mouse FDB, QF, and TP [36], the overall muscle protein abundance was similar among the muscle types analyzed. No statistically significant differences were observed in more than 94% of the total master proteins in the skeletal muscle database, when we compared WT and ASM^−/−^ QF, FDB, and TP samples (Fig. 3c, Additional file 1: Figure S1C, Additional file 5: Table S4, Additional file 6: Table S5). This common core of similarly expressed and functionally important skeletal muscle proteins includes several proteins involved in Ca^2+^ handling, E-C coupling, and sarcolemma integrity, such as the voltage-sensing dihydropyridine receptor (DHPR), the ryanodine receptor (RYR), the SR Ca^2+^ ATPase (SERCA), dystrophin, desmin, tropomyosin, troponin C, and calpains. Interestingly, among the few proteins from the skeletal muscle subset that were significantly altered in expression level between WT and ASM^−/−^ muscles was calsequestrin, which was downregulated in all three muscles from ASM^−/−^ mice (Fig. 3c, Additional file 1: Figure S1C), a finding confirmed by Western blot analysis (Additional file 1: Figure S1C). Given calsequestrin’s role as a large capacity Ca^2+^-binding protein inside the SR terminal cisterna [37], its reduced expression level may be related, at least in part, to the defect we observed in the EC-coupling Ca^2+^ mobilization assays with ASM^−/−^ FDB fibers (Fig. 1). Dystrobrevin-α was significantly upregulated in ASM^−/−^ FDB and QF samples, but not in TP (Fig. 3c, Additional file 1: Figure S1C). The relevance of this finding is not clear, considering that other components of the dystrophin-associated complex such as dystrophin, dystroglycan, sarcoglycan, and syntrophins were not significantly altered (Additional file 5: Table S4, Additional file 6: Table S5). In FDB samples, but not in QF or TP, the proteomic analyses revealed a borderline significant upregulation of the SR Ca^2+^ ATPase SERCA1 in ASM^−/−^ samples (Fig. 3c). Given the importance of SERCA1 for transferring Ca^2+^ from the cytosol to the lumen of the SR in skeletal muscle [38] and the Ca^2+^ mobilization defect we observed in FDB fibers, we followed up on this observation by examining SERCA1 expression levels in four biological replicates of WT and ASM^−/−^ FDB fibers by Western blot with specific antibodies to SERCA1. Although a slight apparent increase in SERCA1 content (relative to the tubulin amount in the same samples) was observed in ASM^−/−^ FDB, the difference was not statistically significant (Fig. 2d). Taken together, our proteomic analyses indicate that deletion of the gene encoding ASM (*SMPD1*) alters the expression of only a small number of proteins known to be associated with skeletal muscle function. Among these, the downregulation of calsequestrin in ASM^−/−^ samples may be functionally important, since it occurred in the three different muscles analyzed.

### Repair of the sarcolemma after injury is impaired in ASM^−/−^ muscle fibers

**Fig. 4:**
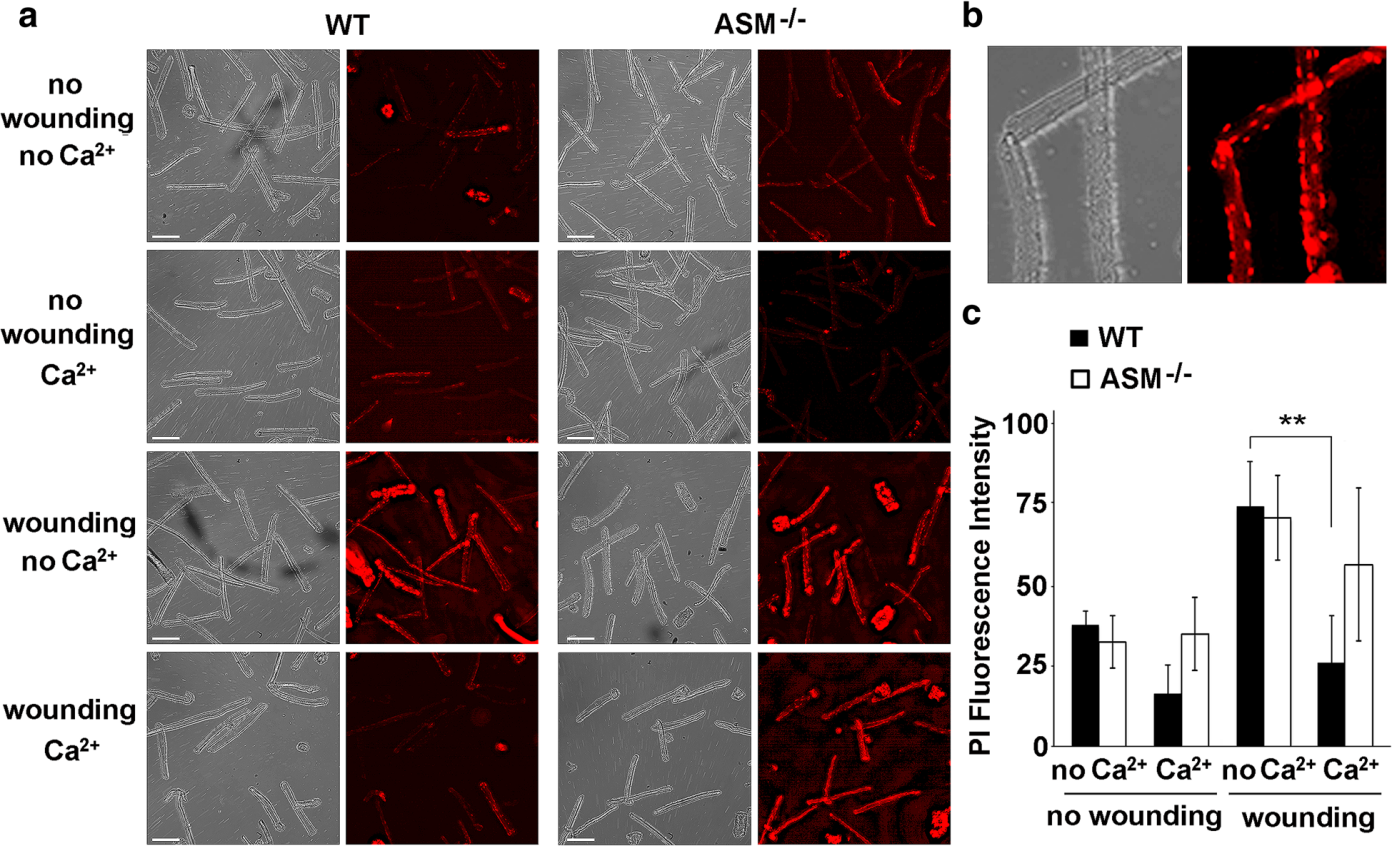
FDB fibers from ASM^−/−^ mice are defective in Ca^2+^-dependent repair of the sarcolemma after mechanical injury. FDB fibers isolated from ASM-deficient mice (ASM^−/−^) and their wild type littermates (WT) were left intact or injured by one passage through a 30-gauge needle. After 5 min at 37 °C, the fibers were exposed to PI, which only enters muscle fibers that sustained a sarcolemmal damage. **a** Brightfield and fluorescent images of PI-stained fibers with or without needle wounding, in the presence (membrane repair permissive condition) or absence of Ca^2+^ (membrane repair non-permissive condition). **b** Enlarged image showing PI staining of peripheral nuclei in wounded fibers. **c** Quantification of PI fluorescence associated with un-contracted fibers (mean ± SE of ~ 200 fibers/animal/condition, for each experiment). The data are representative of three independent experiments. WT fibers showed the expected Ca^2+^-dependent sarcolemma repair (**p = 0.040, WT + Ca^2+^ vs. no Ca^2+^), but no significant resealing was observed in ASM^−/−^ fibers (*p* = 0.668 ASM^−/−^ + Ca^2+^ vs. no Ca^2+^)

Previous studies showed that muscle fibers rapidly reseal wounds in their sarcolemma in a Ca^2+^-dependent manner [25, 39]. However, so far, most studies of the muscle repair process have involved non-physiological forms of wounding such as burning holes on the sarcolemma of individual fibers with lasers, an approach that only allows analysis of small numbers of individual fibers in each assay [40]. To overcome these problems, we developed a population assay where the resealing capacity of hundreds of skeletal muscle fibers could be assessed simultaneously after mechanical injury. We injured isolated mouse FDB muscle fibers in one rapid step, by passage through a 30-gauge needle. In the absence of Ca^2+^, a condition that prevents PM repair [41], passage of muscle fibers through the needle followed by exposure to the membrane impermeant dye propidium iodide (PI) revealed sarcolemma injury in the majority of fibers, detected as PI-stained nuclei. On the other hand, wounded fibers from wild type mice (WT) were able to reseal in the presence of Ca^2+^, as indicated by the significant reduction of PI staining compared to the Ca^2+^ free condition (Fig. 4a, b).

Quantification by image analysis of large numbers of individual fibers (> 200 for each condition) showed that the amount of intracellular PI (fluorescence intensity) in WT and ASM^−/−^ fibers after injury in the absence of Ca^2+^ was similar, indicating that ASM^−/−^ fibers were no more susceptible than WT to mechanical injury (Fig. 4c). In contrast, when the assay was performed in the presence of Ca^2+^ (a condition required for sarcolemma repair), the intracellular PI fluorescence intensity in WT fibers, reflecting sarcolemma permeability, showed baseline levels similar to those seen in fibers not subjected to the wounding procedure. Fibers from ASM^−/−^ mice, in contrast, did not show a significant difference in PI staining in the presence of Ca^2+^, a finding that is consistent with a defect in sarcolemma repair after injury (Fig. 4c).

**Fig. 5:**
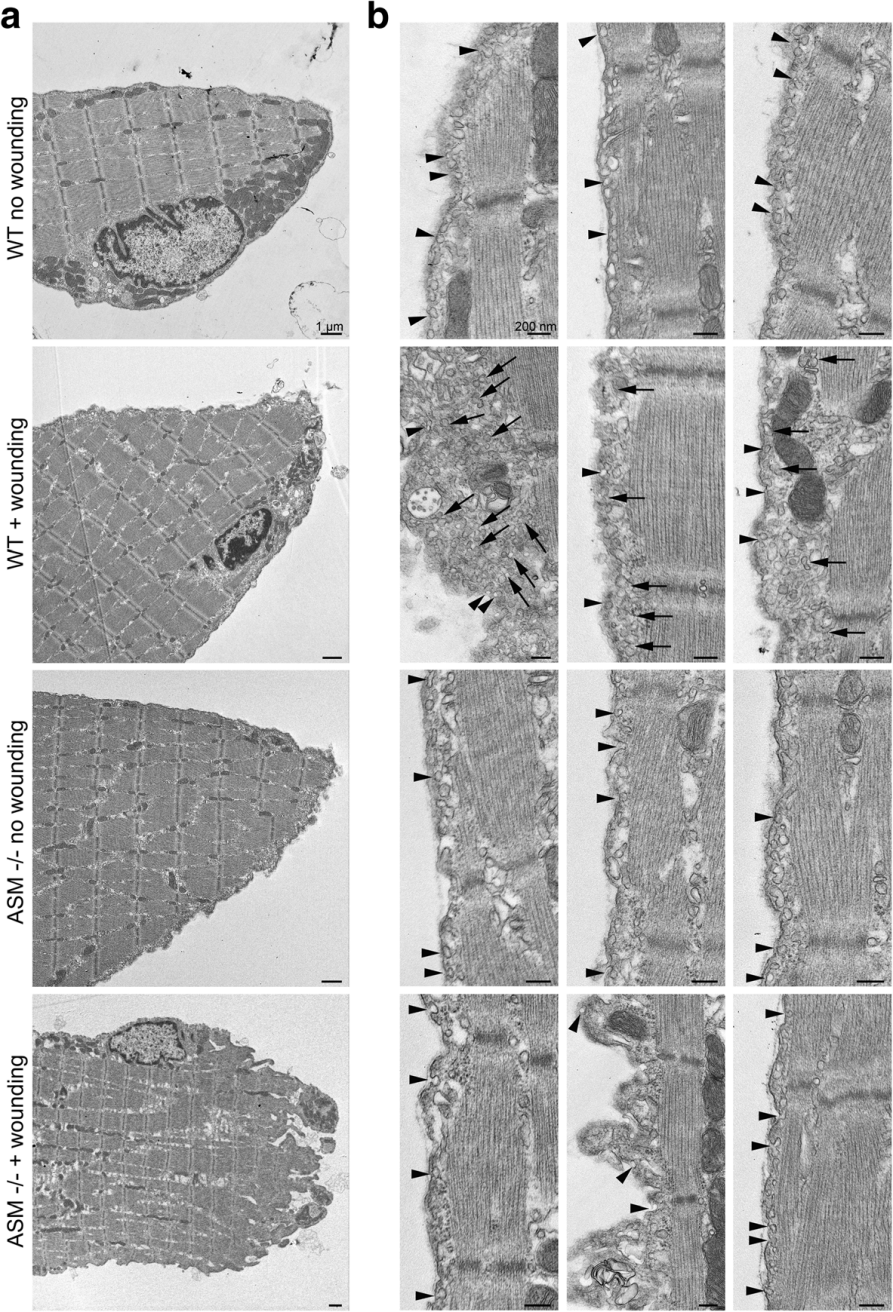
ASM deficiency impairs the rapid intracellular accumulation of caveolar vesicles after injury, resulting in abnormal fiber morphology. FDB fibers isolated from ASM-deficient mice (ASM^−/−^) and their wild type littermates (WT) were left intact or injured by one passage through a 30-gauge needle. After 1 min at 37 °C, the fibers were fixed and processed for TEM. **a** Lower magnification TEM images of the overall ultrastructural morphology of WT or ASM^−/−^ fibers with or without wounding. Bars, 1 μm. **b** TEM images of three independent fibers per condition at higher magnification. The arrowheads point to caveolae on the sarcolemma, and the arrows point to intracellular caveolar vesicles. Bars, 200 nm. The images in **a** and **b** are representative of multiple images acquired from a total of approximately 40 individual fibers

Earlier studies identified a role for sphingomyelinase-mediated caveolar endocytosis in the repair of sarcolemma injury in mouse FDB fibers [25]. To examine if ASM deficiency was associated with impaired sarcolemma repair and reduced caveolar endocytosis, we examined by TEM WT and ASM^−/−^ fibers subjected or not to the needle passage wounding protocol, followed by a brief repair period. The overall morphology of uninjured WT and ASM^−/−^ fibers was similar, with no obvious abnormalities. In contrast, wounded ASM^−/−^ fibers were visibly altered, with markedly irregular borders and an increase in intra-fiber low electron-density material (Fig. 5a). Single rows of the typical < 100 nm PM invaginations known as caveolae were present along the sarcolemma of uninjured WT and ASM^−/−^ fibers (Fig. 5b, arrowheads). As previously described [25], after injury and repair, the sub-sarcolemmal region of WT fibers contained a large number of internalized caveolar vesicles (Fig. 5b, arrows), consistent with their previously proposed role in membrane repair. Strikingly, however, this extensive intracellular accumulation of caveolar vesicles was absent in ASM^−/−^ fibers (Fig. 5b). Thus, ASM deficiency is associated with impaired caveolar endocytosis and defective sarcolemma repair.

### ASM deficiency impairs the ability of skeletal muscle to recover from injury in vivo

Recovery from some types of acute muscle injury can occur without significant levels of myogenic cell proliferation [16], suggesting a role for rapid sarcolemma repair (which is known to occur in less than 1 min [13, 15]). Based on the defective sarcolemmal repair observed after needle-injury, and the impaired ability to withstand repeated tetanic contractions observed in isolated FDB fibers from ASM^−/−^ mice (Figs. 1 and 3), we hypothesized that ASM deficiency might also interfere with the ability of skeletal muscle to recover from an acute large strain injury in vivo.

**Fig. 6:**
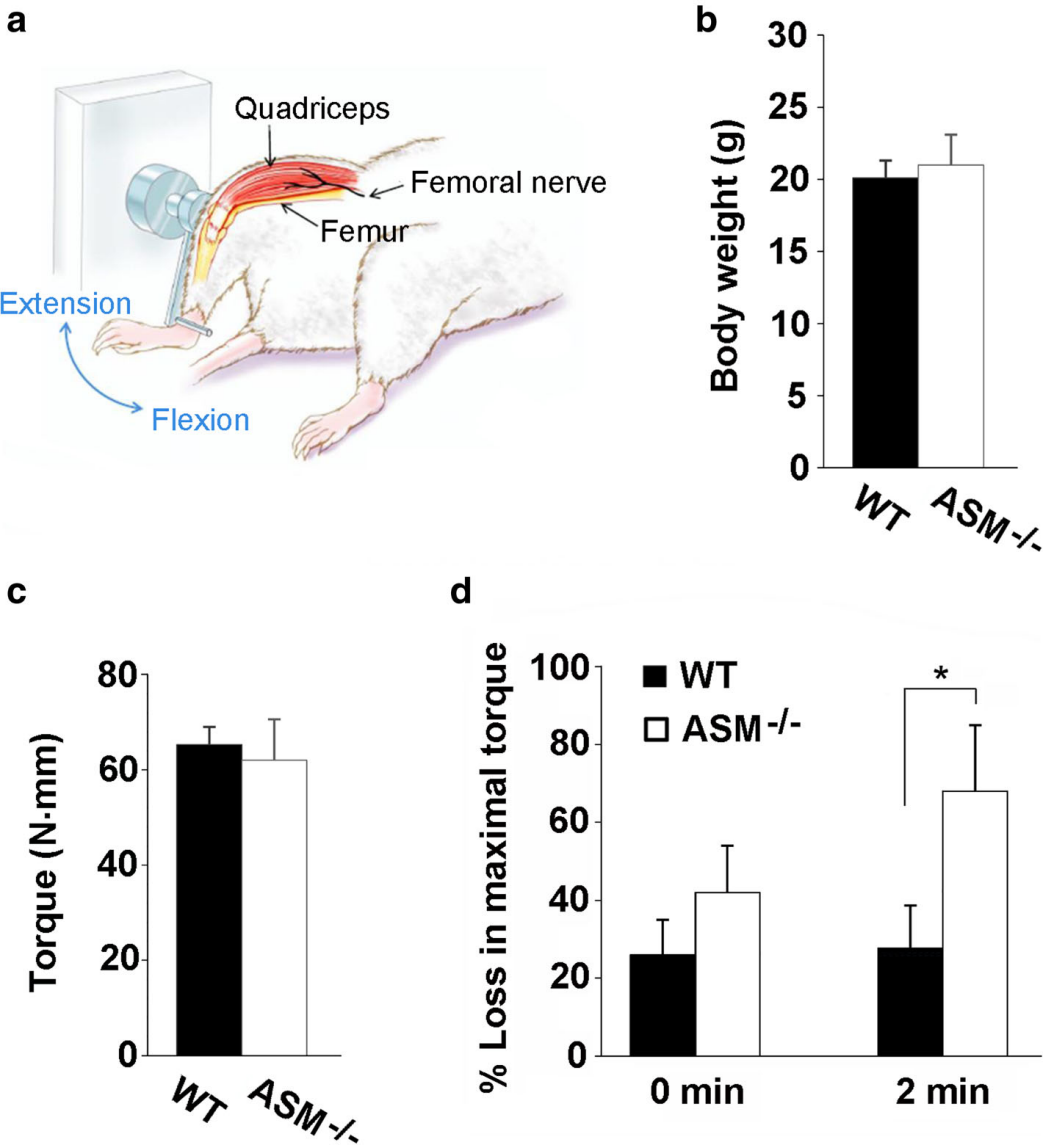
ASM deficiency impairs the ability of skeletal muscle to recover from injury in vivo. **a** Apparatus for muscle injury and isometric torque measurements. The thigh of an anesthetized mouse is stabilized and the leg attached to a motor-driven arm. To assess quadriceps maximal isometric torque, the femoral nerve is stimulated with transcutaneous electrodes, resulting in knee extension (see blue arrow). To induce injury, the lever arm is forced down into flexion while the quadriceps muscle is fully contracted. Image modified from [32], used with permission. **b** Average body weight of WT and ASM^−/−^ littermate mice. No significant differences in body mass were observed between WT and ASM^−/−^ mice. **c** QF absolute strength, as measured by maximal isometric torque before injury. No significant differences were observed between WT and ASM^−/−^ littermate mice. **d** Percent loss of maximal isometric torque in QF muscle immediately after (0 min) or 2 min after a high-force lengthening contraction injury in WT or ASM^−/−^ littermate mice. ASM^−/−^ mice showed a significantly higher loss of maximal isometric torque at 2-min post-injury when compared to WT (*p = 0.0023). All data represent the mean ± SD of seven mice in each group

We tested this hypothesis using an in vivo animal model developed for testing the contractile force of QF muscle (Fig. 6a). This model results in the production of a reliable muscle injury and allows assessment of muscle functional recovery (maximal isometric torque) within the same animal over time [30]. Using this model, we found no significant differences in the maximal isometric torque (with and without normalization for body weight) in the QF of WT and ASM^−/−^ littermate mice prior to injury (Fig. 6b, c). The body weights were not significantly different between WT and ASM^−/−^ mice, suggesting no overall muscle atrophy or hypertrophy (Fig. 6b). There was also no significant difference in the amount of injury induced by forced lengthening contractions, as indicated by the maximal isometric torque measured immediately after (0 min) the large strain eccentric injury (Fig. 6c). However, after 2 min (a time period chosen for consistency with the fast sarcolemma resealing process, and also to avoid secondary effects of inflammation), a significant difference was observed: while the loss in maximal isometric torque of WT mice (28%) was unchanged compared to 0 min, in ASM^−/−^ mice, the loss was increased to 68% (Fig. 6d). These results suggest that QF muscles of ASM^−/−^ mice are impaired in recovery from a large strain injury, possibly as a consequence of defective sarcolemma repair, as observed in FDB fibers (Figs. 4 and 5).

## Discussion

In this study, we examined several aspects of skeletal muscle function in ASM-deficient mice. Over time, these animals develop a phenotype that closely mimics the human diseases NPDA/B, including severe neurodegeneration and a shortened life span [23]. We limited our studies to the first 8 weeks of life of the animals, prior to the onset of neurological symptoms. First, we found that isolated FDB fibers from ASM^−/−^ mice exhibit lower levels of peak intracellular Ca^2+^ after excitation in an E-C coupling assay and have a more rapid decline in peak tetanic Ca^2+^ in response to repeated tetanic contractions. Second, using a quantitative proteomic approach, we detected the downregulation of the SR Ca^2+^-buffering protein calsequestrin in the three different skeletal muscles (FDB, QF, and TP) from ASM-deficient animals, while > 90% of proteins associated with skeletal muscle function were similarly expressed in ASM^−/−^ and WT mice. Third, using an in vitro assay for assessing mechanical wounding and sarcolemma repair in isolated FDB fibers, we found that ASM deficiency impairs the ability of FDB fibers to reseal their sarcolemma after injury. Finally, in vivo measurements of QF absolute strength before and immediately after a lengthening contraction-induced injury revealed no significant differences between WT and ASM^−/−^ animals, suggesting similar susceptibility to injury. However, a significantly larger loss of maximal isometric torque was observed in the QF of ASM^−/−^ mice when the animals were tested 2-min post-injury, a period that is known to be sufficient for full repair of PM wounds [8]. Thus, our study provides direct evidence that ASM deficiency impairs skeletal muscle function, possibly as a consequence of defective sarcolemma repair after injury.

The degree of impairment from contraction-induced injury depends not only on the muscle, but also on the timing of muscle activation to lengthening, the amount of muscle strain, and the velocity of movement [42]. Muscle lengthening (“eccentric”) contractions can result in a significant and long-lasting deficit of force production, but this requires a maximal contraction before and during lengthening, as well as a high number of repetitions. Such protocols show moderate susceptibility to injury in healthy WT mice, but severe susceptibility to damage in animals with muscle diseases, such as *mdx* mice, the murine model for Duchenne muscular dystrophy. Here, we used a model of only 10 repetitions, which induces a mild loss in muscle maximal force. An initial recovery that occurs after 10 repetitions has been described before [43] and precedes the further drop in force that is seen days later [16, 44]. Long-term follow-up after injury was not the focus of our study, as this involves mechanisms beyond immediate sarcolemma resealing, such as cytoskeletal restructuring and a peak of inflammation followed by eventual resolution [45].

ASM deficiency was reported to cause overaccumulation of sphingomyelin in the PM and lysosomes of ASM^−/−^ mouse fibroblasts, a process proposed to contribute to membrane instability [46]. An overabundance of PM sphingomyelin was also reported in spermatozoa of ASM^−/−^ mice, of which only 13.4% showed intact membranes in a permeability assay [47]. However, only a very limited number of studies have evaluated a potential impact of ASM deficiency on skeletal muscle function. McGovern et al. [48] found impaired pulmonary gas exchange in NPDB patients using maximal exercise tolerance testing, and Macauley et al. [49] found that the performance of ASM^−/−^ mice on Rotarod tests deteriorated after 7 weeks of age. In view of our current findings, it is conceivable that these previously observed functional defects are related to impaired muscle function.

Previous studies identified ASM as a critical component of the mechanism of PM repair after injury. ASM is secreted from lysosomes of cultured cells in response to PM injury in the presence of Ca^2+^, and inhibition or RNAi depletion of ASM strongly impairs the ability of cells to repair wounds in their PM, in a process mediated by endocytosis [8]. Similar results were obtained with ASM-deficient fibroblasts derived from NPDA patients, and extracellular addition of purified ASM fully rescued the PM repair defect of these cells [8]. Subsequent studies showed that myotubes and isolated mouse FDB muscle fibers show enhanced internalization of caveolar vesicles after wounding or after extracellular exposure to ASM, raising the possibility that ASM may regulate the repair of sarcolemma wounds by promoting lesion removal by endocytosis [25]. In the present study, we obtained further evidence in support of this hypothesis, by demonstrating that isolated FDB fibers have a significantly reduced ability to repair their sarcolemma after mechanical injury.

ASM-deficient FDB fibers exhibited a significant reduction in the average time to reach 50% of the initial peak Fura-2 ratio during repeated tetanic contractions, a parameter that was previously associated with muscle fatigue [28]. This finding may reflect a reduced ability of the fibers to repair sarcolemma wounds inflicted during repeated contraction-relaxation cycles. We also observed lower peak [Ca^2+^]_i_ in ASM^−/−^ FDB during baseline measurements in the presence of Ca^2+^. This suggests a defect prior to or during Ca^2+^ release from the SR in response to electrical stimulation. Propagation of the action potential across the sarcolemma and the T-tubules is important for activating SR-Ca^2+^ release, through critical interactions between the voltage-sensing receptor DHPR and the SR Ca^2+^ release channel RYR. Membrane depolarization defects are expected to disrupt voltage-sensing by the DHPR, reducing SR-Ca^2+^ release [50]. Thus, changes in T-tubule and/or SR membrane composition as a consequence of ASM deficiency may also be considered as a potential explanation for our observations. However, the lower peak [Ca^2+^]_i_ of ASM^−/−^ FDB during baseline measurements in E-C assays may also be related to a defect in sarcolemma repair, since ASM^−/−^ fibers with unrepaired wounds may have a reduced capacity to propagate an action potential, leading to diminished SR-Ca^2+^ release. Finally, the reduction in calsequestrin detected in our proteomic analyses is likely responsible for the reductions in [Ca^2+^]_i_ at the high stimulation frequencies and may also be responsible for the altered response to repeated tetanic contractions. Previous studies in calsequestrin knockout mice showed that peak intracellular Ca^2+^ levels are reduced during sustained tetanic contractions, due to depletion of SR Ca^2+^ stores [51].

## Conclusions

We report a novel phenotype in a mouse model of NPDA/B that lacks the lysosomal enzyme ASM. Skeletal muscle from ASM^−/−^ mice have an impairment in intracellular Ca^2+^ handling that results in reduced Ca^2+^ mobilization and a more rapid decline in peak Ca^2+^ transients during repeated contraction-relaxation cycles. Isolated skeletal muscle fibers show an impaired ability to repair sarcolemma damage, and this was associated with an exaggerated deficit in force during recovery from an in vivo muscle injury. Our findings in this animal model suggest that specific skeletal muscle phenotypes may have been previously overlooked in NPDA/B patients and deserve further investigation.

## Additional files

Additional file 1: **Figure S1.** Comparative proteomic analysis of tibialis posterior (TP) muscle fibers from WT and ASM^−/−^ mice. (A) Protein abundance values for three biological replicates of WT or ASM^−/−^ TP muscle isolated from WT and ASM^−/−^ mice. (B) Volcano plots indicating statistically significant (*P* < 0.05) differences between WT and ASM^−/−^ samples in the expression of all master proteins identified for TP muscle. Green box, proteins downregulated more than twofold in ASM^−/−^ relative to WT; pink box, proteins upregulated more than twofold in ASM^−/−^ relative to WT. (C) Volcano plots indicating statistically significant (*P* < 0.05) differences between WT and ASM^−/−^ samples in the expression of master proteins within a subset of functionally important skeletal muscle proteins in TP muscle. Green box, proteins downregulated more than twofold in ASM^−/−^ relative to WT; pink box, proteins upregulated more than twofold in ASM^−/−^ relative to WT. (D) QF, FDB, and TP muscles isolated from two WT mice and two ASM^−/−^ mice were solubilized and analyzed by Western blot with anti-calsequestrin antibodies, which detected doublet bands of the predicted size of 50–55 kDa (each lane corresponds to fibers isolated from one animal). Antibodies against actin were used as a loading control. Calsequestrin/actin ratio densitometry values are shown above each lane, validating the reduced expression of calsequestrin in ASM^−/−^ mice. (TIF 638 kb)

Additional file 2: **Table S1.** Total master proteins detected in QF muscle. (DOCX 163 kb)

Additional file 3: **Table S2.** Total master proteins detected in FDB muscle. (DOCX 165 kb)

Additional file 4: **Table S3.** Total master proteins detected in TP muscle. (DOCX 164 kb)

Additional file 5: **Table S4.** Skeletal muscle master protein subset. (DOCX 57 kb)

Additional file 6: **Table S5.** Master proteins from a skeletal muscle subset significantly up- or downregulated in QF, FDB, or TP. (DOCX 23 kb)
