# Supplementary figures and images for: Defects in sarcolemma repair and skeletal muscle function after injury in a mouse model of Niemann-Pick type A/B disease
Source: Skelet Muscle. 2019 Jan 5;9:1. doi: 10.1186/s13395-018-0187-5 (PMC6320626; doi:10.1186/s13395-018-0187-5)

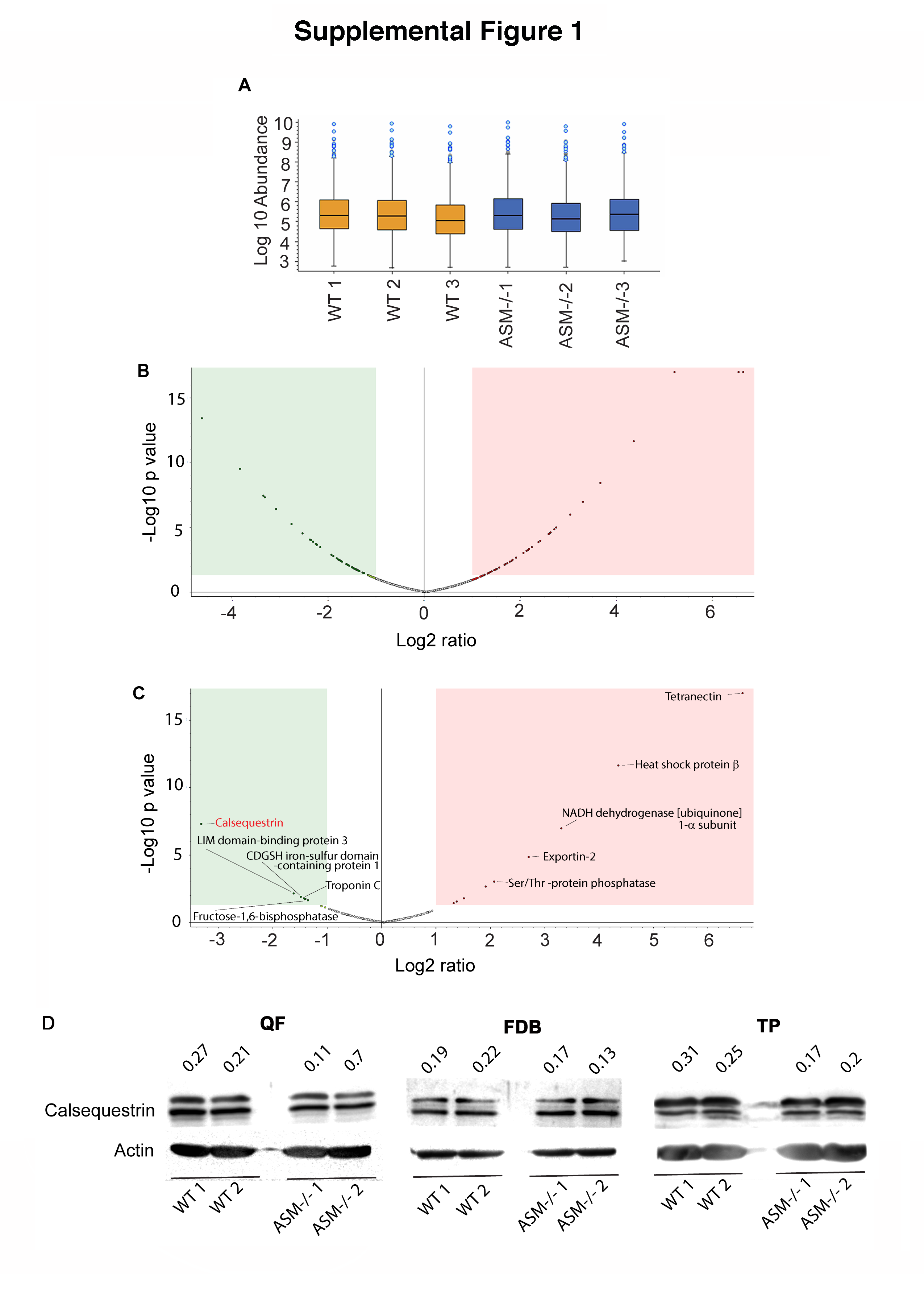

Supplement: Supplementary file 1 — Figure S1. Comparative proteomic analysis of tibialis posterior (TP) muscle fibers from WT and ASM−/− mice. (A) Protein abundance values for three biological replicates of WT or ASM−/− TP muscle isolated from WT and ASM−/− mice. (B) Volcano plots indicating statistically significant (P < 0.05) differences between WT and ASM−/− samples in the expression of all master proteins identified for TP muscle. Green box, proteins downregulated more than twofold in ASM−/− relative to WT; pink box, proteins upregulated more than twofold in ASM−/− relative to WT. (C) Volcano plots indicating statistically significant (P < 0.05) differences between WT and ASM−/− samples in the expression of master proteins within a subset of functionally important skeletal muscle proteins in TP muscle. Green box, proteins downregulated more than twofold in ASM−/− relative to WT; pink box, proteins upregulated more than twofold in ASM−/− relative to WT. (D) QF, FDB, and TP muscles isolated from two WT mice and two ASM−/− mice were solubilized and analyzed by Western blot with anti-calsequestrin antibodies, which detected doublet bands of the predicted size of 50–55 kDa (each lane corresponds to fibers isolated from one animal). Antibodies against actin were used as a loading control. Calsequestrin/actin ratio densitometry values are shown above each lane, validating the reduced expression of calsequestrin in ASM−/− mice. (TIF 638 kb) [file 13395_2018_187_MOESM1_ESM.tif]
