# Supplementary material for: Defects in sarcolemma repair and skeletal muscle function after injury in a mouse model of Niemann-Pick type A/B disease
Source: Skelet Muscle. 2019 Jan 5;9:1. doi: 10.1186/s13395-018-0187-5 (PMC6320626; doi:10.1186/s13395-018-0187-5)
Supplement: Supplementary file 4 — Table S3. Total master proteins detected in TP muscle. (DOCX 164 kb) [file 13395_2018_187_MOESM4_ESM.docx]

**Table S3. Total Master Proteins Detected in TP Muscle.**

Green, master proteins downregulated in ASM^-/-^ relative to WT.

Pink, master proteins upregulated in ASM^-/-^ relative to WT.

| **Uniprot Accession #** | **Description** | **Abundance Ratio: (TP, KO) / (TP, WT)** | **Abundance Ratio P-Value: (TP, KO) / (TP, WT)** |
| --- | --- | --- | --- |
| Q9WVH9 | Fibulin-5 OS=Mus musculus GN=Fbln5 PE=1 SV=1 | 0.041 | 0.000 |
| Q3UBX0 | Transmembrane protein 109 OS=Mus musculus GN=Tmem109 PE=1 SV=2 | 0.07 | 0.000 |
| Q07076 | Annexin A7 OS=Mus musculus GN=Anxa7 PE=1 SV=2 | 0.098 | 0.000 |
| O09161 | Calsequestrin-2 OS=Mus musculus GN=Casq2 PE=1 SV=3 | 0.101 | 0.000 |
| E9PYJ9 | LIM domain-binding protein 3 OS=Mus musculus GN=Ldb3 PE=1 SV=1 | 0.119 | 0.000 |
| Q6PHN9 | Ras-related protein Rab-35 OS=Mus musculus GN=Rab35 PE=1 SV=1 | 0.148 | 0.000 |
| O35639 | Annexin A3 OS=Mus musculus GN=Anxa3 PE=1 SV=4 | 0.174 | 0.000 |
| Q9D1G1 | Ras-related protein Rab-1B OS=Mus musculus GN=Rab1b PE=1 SV=1 | 0.193 | 0.000 |
| O35864 | COP9 signalosome complex subunit 5 OS=Mus musculus GN=Cops5 PE=1 SV=3 | 0.196 | 0.000 |
| D3YZD8 | Mth938 domain-containing protein OS=Mus musculus GN=Aamdc PE=1 SV=1 | 0.202 | 0.000 |
| A0A0R4J036 | Neurofilament 3, medium OS=Mus musculus GN=Nefm PE=1 SV=1 | 0.211 | 0.000 |
| Q8BMK4 | Cytoskeleton-associated protein 4 OS=Mus musculus GN=Ckap4 PE=1 SV=2 | 0.213 | 0.000 |
| A2RSB1 | Nucleosome assembly protein 1-like 4 OS=Mus musculus GN=Nap1l4 PE=1 SV=1 | 0.214 | 0.000 |
| Q63810 | Calcineurin subunit B type 1 OS=Mus musculus GN=Ppp3r1 PE=1 SV=3 | 0.224 | 0.000 |
| Q8R5L1 | Complement component 1 Q subcomponent-binding protein, mitochondrial OS=Mus musculus GN=C1qbp PE=1 SV=1 | 0.263 | 0.001 |
| Q8BLY2 | Probable threonine--tRNA ligase 2, cytoplasmic OS=Mus musculus GN=Tarsl2 PE=1 SV=1 | 0.271 | 0.002 |
| Q9CPU4 | Microsomal glutathione S-transferase 3 OS=Mus musculus GN=Mgst3 PE=1 SV=1 | 0.284 | 0.003 |
| P60335 | Poly(rC)-binding protein 1 OS=Mus musculus GN=Pcbp1 PE=1 SV=1 | 0.291 | 0.003 |
| O09131 | Glutathione S-transferase omega-1 OS=Mus musculus GN=Gsto1 PE=1 SV=2 | 0.293 | 0.003 |
| F6RT34 | Myelin basic protein (Fragment) OS=Mus musculus GN=Mbp PE=1 SV=1 | 0.295 | 0.003 |
| Q60715 | Prolyl 4-hydroxylase subunit alpha-1 OS=Mus musculus GN=P4ha1 PE=1 SV=2 | 0.295 | 0.003 |
| Q3TPZ5 | Dynactin 2 OS=Mus musculus GN=Dctn2 PE=1 SV=1 | 0.299 | 0.004 |
| Q9JMH6 | Thioredoxin reductase 1, cytoplasmic OS=Mus musculus GN=Txnrd1 PE=1 SV=3 | 0.303 | 0.004 |
| Q8BP47 | Asparagine--tRNA ligase, cytoplasmic OS=Mus musculus GN=Nars PE=1 SV=2 | 0.307 | 0.005 |
| P03888 | NADH-ubiquinone oxidoreductase chain 1 OS=Mus musculus GN=Mtnd1 PE=1 SV=3 | 0.307 | 0.005 |
| Q9JKS4-6 | Isoform 6 of LIM domain-binding protein 3 OS=Mus musculus GN=Ldb3 | 0.327 | 0.007 |
| J3QPW1 | Phosphatidylinositol transfer protein alpha isoform OS=Mus musculus GN=Pitpna PE=1 SV=1 | 0.328 | 0.007 |
| Q9DAK9 | 14 kDa phosphohistidine phosphatase OS=Mus musculus GN=Phpt1 PE=1 SV=1 | 0.334 | 0.008 |
| O88543 | COP9 signalosome complex subunit 3 OS=Mus musculus GN=Cops3 PE=1 SV=3 | 0.337 | 0.009 |
| Q7JCY6 | NADH-ubiquinone oxidoreductase chain 4 OS=Mus musculus GN=mt-Nd4 PE=1 SV=1 | 0.34 | 0.009 |
| Q9D8L3 | Signal sequence receptor, delta OS=Mus musculus GN=Ssr4 PE=1 SV=1 | 0.354 | 0.012 |
| E9Q557 | Desmoplakin OS=Mus musculus GN=Dsp PE=1 SV=1 | 0.356 | 0.013 |
| Q91WS0 | CDGSH iron-sulfur domain-containing protein 1 OS=Mus musculus GN=Cisd1 PE=1 SV=1 | 0.358 | 0.013 |
| Q99JZ4 | GTP-binding protein SAR1a OS=Mus musculus GN=Sar1a PE=1 SV=1 | 0.363 | 0.014 |
| Q9CQV8 | 14-3-3 protein beta/alpha OS=Mus musculus GN=Ywhab PE=1 SV=3 | 0.364 | 0.015 |
| P84078 | ADP-ribosylation factor 1 OS=Mus musculus GN=Arf1 PE=1 SV=2 | 0.365 | 0.015 |
| P13020 | Gelsolin OS=Mus musculus GN=Gsn PE=1 SV=3 | 0.366 | 0.015 |
| P99027 | 60S acidic ribosomal protein P2 OS=Mus musculus GN=Rplp2 PE=1 SV=3 | 0.371 | 0.017 |
| P19123 | Troponin C, slow skeletal and cardiac muscles OS=Mus musculus GN=Tnnc1 PE=1 SV=1 | 0.373 | 0.017 |
| P70695 | Fructose-1,6-bisphosphatase isozyme 2 OS=Mus musculus GN=Fbp2 PE=1 SV=2 | 0.375 | 0.018 |
| Q3UGR5 | Haloacid dehalogenase-like hydrolase domain-containing protein 2 OS=Mus musculus GN=Hdhd2 PE=1 SV=2 | 0.378 | 0.019 |
| P58281-2 | Isoform 2 of Dynamin-like 120 kDa protein, mitochondrial OS=Mus musculus GN=Opa1 | 0.38 | 0.019 |
| Q64737 | Trifunctional purine biosynthetic protein adenosine-3 OS=Mus musculus GN=Gart PE=1 SV=3 | 0.381 | 0.020 |
| Q8BGD9 | Eukaryotic translation initiation factor 4B OS=Mus musculus GN=Eif4b PE=1 SV=1 | 0.382 | 0.020 |
| P62196 | 26S protease regulatory subunit 8 OS=Mus musculus GN=Psmc5 PE=1 SV=1 | 0.386 | 0.022 |
| G3UY93 | Valine--tRNA ligase (Fragment) OS=Mus musculus GN=Vars PE=1 SV=1 | 0.388 | 0.022 |
| Q60605 | Myosin light polypeptide 6 OS=Mus musculus GN=Myl6 PE=1 SV=3 | 0.392 | 0.024 |
| P62960 | Nuclease-sensitive element-binding protein 1 OS=Mus musculus GN=Ybx1 PE=1 SV=3 | 0.394 | 0.024 |
| Q922F4 | Tubulin beta-6 chain OS=Mus musculus GN=Tubb6 PE=1 SV=1 | 0.413 | 0.032 |
| E9PWZ3 | Protein Rpl3l OS=Mus musculus GN=Rpl3l PE=1 SV=1 | 0.413 | 0.032 |
| Q8VCF0 | Mitochondrial antiviral-signaling protein OS=Mus musculus GN=Mavs PE=1 SV=1 | 0.417 | 0.034 |
| P00405 | Cytochrome c oxidase subunit 2 OS=Mus musculus GN=Mtco2 PE=1 SV=1 | 0.42 | 0.035 |
| B2RVP5 | Histone H2A OS=Mus musculus GN=H2afv PE=1 SV=1 | 0.42 | 0.036 |
| P26645 | Myristoylated alanine-rich C-kinase substrate OS=Mus musculus GN=Marcks PE=1 SV=2 | 0.422 | 0.036 |
| Q91VB8 | Alpha globin 1 OS=Mus musculus GN=Hba-a1 PE=1 SV=1 | 0.446 | 0.050 |
| A8DUK4 | Beta-globin OS=Mus musculus GN=Hbbt1 PE=1 SV=1 | 0.446 | 0.050 |
| Q921F2 | TAR DNA-binding protein 43 OS=Mus musculus GN=Tardbp PE=1 SV=1 | 0.45 | 0.052 |
| Q00898 | Alpha-1-antitrypsin 1-5 OS=Mus musculus GN=Serpina1e PE=1 SV=1 | 0.451 | 0.053 |
| H3BLC7 | AMP deaminase 1 (Fragment) OS=Mus musculus GN=Ampd1 PE=1 SV=7 | 0.451 | 0.053 |
| Q8R404 | MICOS complex subunit MIC13 OS=Mus musculus GN=Mic13 PE=1 SV=1 | 0.455 | 0.056 |
| A0A0G2JGS4 | Calcium/calmodulin-dependent protein kinase type II subunit delta OS=Mus musculus GN=Camk2d PE=1 SV=1 | 0.463 | 0.061 |
| P04247 | Myoglobin OS=Mus musculus GN=Mb PE=1 SV=3 | 0.465 | 0.062 |
| Q02357-7 | Isoform Mu7 of Ankyrin-1 OS=Mus musculus GN=Ank1 | 0.466 | 0.063 |
| Q922B2 | Aspartate--tRNA ligase, cytoplasmic OS=Mus musculus GN=Dars PE=1 SV=2 | 0.467 | 0.064 |
| E9QPX1 | Collagen alpha-1(XVIII) chain OS=Mus musculus GN=Col18a1 PE=1 SV=1 | 0.467 | 0.064 |
| P28474 | Alcohol dehydrogenase class-3 OS=Mus musculus GN=Adh5 PE=1 SV=3 | 0.473 | 0.068 |
| Q9CYR0 | Single-stranded DNA-binding protein, mitochondrial OS=Mus musculus GN=Ssbp1 PE=1 SV=1 | 0.473 | 0.068 |
| Q9CQJ8 | NADH dehydrogenase [ubiquinone] 1 beta subcomplex subunit 9 OS=Mus musculus GN=Ndufb9 PE=1 SV=3 | 0.475 | 0.069 |
| Q8R5J9 | PRA1 family protein 3 OS=Mus musculus GN=Arl6ip5 PE=1 SV=2 | 0.475 | 0.070 |
| A6H644 | Protein phosphatase 1 regulatory subunit OS=Mus musculus GN=Ppp1r12b PE=1 SV=1 | 0.48 | 0.074 |
| Q3UV17 | Keratin, type II cytoskeletal 2 oral OS=Mus musculus GN=Krt76 PE=1 SV=1 | 0.481 | 0.075 |
| P62281 | 40S ribosomal protein S11 OS=Mus musculus GN=Rps11 PE=1 SV=3 | 0.485 | 0.077 |
| Q3U7R1 | Extended synaptotagmin-1 OS=Mus musculus GN=Esyt1 PE=1 SV=2 | 0.487 | 0.079 |
| P63328 | Serine/threonine-protein phosphatase 2B catalytic subunit alpha isoform OS=Mus musculus GN=Ppp3ca PE=1 SV=1 | 0.487 | 0.079 |
| P32921 | Tryptophan--tRNA ligase, cytoplasmic OS=Mus musculus GN=Wars PE=1 SV=2 | 0.493 | 0.084 |
| Q3TQX5 | ATP-dependent RNA helicase DDX3X OS=Mus musculus GN=Ddx3x PE=1 SV=1 | 0.497 | 0.088 |
| Q9Z1P6 | NADH dehydrogenase [ubiquinone] 1 alpha subcomplex subunit 7 OS=Mus musculus GN=Ndufa7 PE=1 SV=3 | 0.5 | 0.090 |
| Q60598 | Src substrate cortactin OS=Mus musculus GN=Cttn PE=1 SV=2 | 0.504 | 0.094 |
| Q9Z0X1 | Apoptosis-inducing factor 1, mitochondrial OS=Mus musculus GN=Aifm1 PE=1 SV=1 | 0.504 | 0.094 |
| Q6P9R2 | Serine/threonine-protein kinase OSR1 OS=Mus musculus GN=Oxsr1 PE=1 SV=1 | 0.506 | 0.096 |
| P61979-2 | Isoform 2 of Heterogeneous nuclear ribonucleoprotein K OS=Mus musculus GN=Hnrnpk | 0.508 | 0.097 |
| O55234 | Proteasome subunit beta type-5 OS=Mus musculus GN=Psmb5 PE=1 SV=3 | 0.514 | 0.103 |
| Q99LM3 | Smoothelin-like protein 1 OS=Mus musculus GN=Smtnl1 PE=1 SV=1 | 0.514 | 0.104 |
| Q9R059 | Four and a half LIM domains protein 3 OS=Mus musculus GN=Fhl3 PE=1 SV=2 | 0.517 | 0.106 |
| A0A0R4IZW8 | Calpain small subunit 1 OS=Mus musculus GN=Capns1 PE=1 SV=1 | 0.523 | 0.113 |
| Q9D0E1 | Heterogeneous nuclear ribonucleoprotein M OS=Mus musculus GN=Hnrnpm PE=1 SV=3 | 0.528 | 0.118 |
| Q62000 | Mimecan OS=Mus musculus GN=Ogn PE=1 SV=1 | 0.531 | 0.120 |
| Q9CZ44-3 | Isoform 3 of NSFL1 cofactor p47 OS=Mus musculus GN=Nsfl1c | 0.532 | 0.121 |
| Q9JK42 | [Pyruvate dehydrogenase (acetyl-transferring)] kinase isozyme 2, mitochondrial OS=Mus musculus GN=Pdk2 PE=1 SV=2 | 0.532 | 0.122 |
| Q61029 | Lamina-associated polypeptide 2, isoforms beta/delta/epsilon/gamma OS=Mus musculus GN=Tmpo PE=1 SV=4 | 0.532 | 0.122 |
| Q78IK2 | Up-regulated during skeletal muscle growth protein 5 OS=Mus musculus GN=Usmg5 PE=1 SV=1 | 0.533 | 0.123 |
| Q9CQZ5 | NADH dehydrogenase [ubiquinone] 1 alpha subcomplex subunit 6 OS=Mus musculus GN=Ndufa6 PE=1 SV=1 | 0.534 | 0.124 |
| Q8VDN2 | Sodium/potassium-transporting ATPase subunit alpha-1 OS=Mus musculus GN=Atp1a1 PE=1 SV=1 | 0.536 | 0.126 |
| P15105 | Glutamine synthetase OS=Mus musculus GN=Glul PE=1 SV=6 | 0.537 | 0.127 |
| P31001 | Desmin OS=Mus musculus GN=Des PE=1 SV=3 | 0.537 | 0.127 |
| A0A0N4SV63 | Dysferlin OS=Mus musculus GN=Dysf PE=1 SV=1 | 0.539 | 0.129 |
| Q5M8N4 | Epimerase family protein SDR39U1 OS=Mus musculus GN=Sdr39u1 PE=1 SV=1 | 0.539 | 0.129 |
| Q78ZJ8 | MCG22989, isoform CRA_b OS=Mus musculus GN=Rab11b PE=1 SV=1 | 0.542 | 0.132 |
| P43274 | Histone H1.4 OS=Mus musculus GN=Hist1h1e PE=1 SV=2 | 0.542 | 0.132 |
| Q3U6L3 | Glutaredoxin, isoform CRA_a OS=Mus musculus GN=Glrx PE=1 SV=1 | 0.549 | 0.140 |
| P47753 | F-actin-capping protein subunit alpha-1 OS=Mus musculus GN=Capza1 PE=1 SV=4 | 0.549 | 0.141 |
| O88492 | Perilipin-4 OS=Mus musculus GN=Plin4 PE=1 SV=2 | 0.551 | 0.143 |
| Q6GT24 | Peroxiredoxin 6 OS=Mus musculus GN=Prdx6 PE=1 SV=1 | 0.554 | 0.146 |
| E9PZF0 | Nucleoside diphosphate kinase OS=Mus musculus GN=Gm20390 PE=3 SV=1 | 0.555 | 0.147 |
| P17426 | AP-2 complex subunit alpha-1 OS=Mus musculus GN=Ap2a1 PE=1 SV=1 | 0.557 | 0.150 |
| O35367 | Keratocan OS=Mus musculus GN=Kera PE=2 SV=1 | 0.558 | 0.151 |
| Q99PL5 | Ribosome-binding protein 1 OS=Mus musculus GN=Rrbp1 PE=1 SV=2 | 0.566 | 0.160 |
| P18760 | Cofilin-1 OS=Mus musculus GN=Cfl1 PE=1 SV=3 | 0.566 | 0.161 |
| P13541 | Myosin-3 OS=Mus musculus GN=Myh3 PE=2 SV=2 | 0.568 | 0.163 |
| P03921 | NADH-ubiquinone oxidoreductase chain 5 OS=Mus musculus GN=Mtnd5 PE=1 SV=3 | 0.57 | 0.165 |
| E9Q1G8 | Septin-7 OS=Mus musculus GN=Sept7 PE=1 SV=2 | 0.572 | 0.168 |
| P48774 | Glutathione S-transferase Mu 5 OS=Mus musculus GN=Gstm5 PE=1 SV=1 | 0.572 | 0.169 |
| Q91V79 | Fat storage-inducing transmembrane protein 1 OS=Mus musculus GN=Fitm1 PE=1 SV=1 | 0.572 | 0.169 |
| P09542 | Myosin light chain 3 OS=Mus musculus GN=Myl3 PE=1 SV=4 | 0.574 | 0.171 |
| E9Q2T3 | Protein Tnxb OS=Mus musculus GN=Tnxb PE=1 SV=1 | 0.575 | 0.172 |
| P70349 | Histidine triad nucleotide-binding protein 1 OS=Mus musculus GN=Hint1 PE=1 SV=3 | 0.575 | 0.172 |
| O55142 | 60S ribosomal protein L35a OS=Mus musculus GN=Rpl35a PE=1 SV=2 | 0.577 | 0.174 |
| G3X8R0 | Receptor expression-enhancing protein OS=Mus musculus GN=Reep5 PE=1 SV=1 | 0.577 | 0.175 |
| Q99KC8 | von Willebrand factor A domain-containing protein 5A OS=Mus musculus GN=Vwa5a PE=1 SV=2 | 0.578 | 0.176 |
| O35215 | D-dopachrome decarboxylase OS=Mus musculus GN=Ddt PE=1 SV=3 | 0.579 | 0.177 |
| P82350 | Alpha-sarcoglycan OS=Mus musculus GN=Sgca PE=1 SV=1 | 0.58 | 0.178 |
| Q60994 | Adiponectin OS=Mus musculus GN=Adipoq PE=1 SV=2 | 0.581 | 0.180 |
| Q52L67 | Gpsn2 protein OS=Mus musculus GN=Tecr PE=1 SV=1 | 0.583 | 0.183 |
| Q50HX4 | RAB14 protein OS=Mus musculus GN=Rab14 PE=1 SV=1 | 0.585 | 0.185 |
| P16015 | Carbonic anhydrase 3 OS=Mus musculus GN=Ca3 PE=1 SV=3 | 0.591 | 0.193 |
| G5E8T9 | Hydroxyacyl glutathione hydrolase OS=Mus musculus GN=Hagh PE=1 SV=1 | 0.591 | 0.193 |
| P56135 | ATP synthase subunit f, mitochondrial OS=Mus musculus GN=Atp5j2 PE=1 SV=3 | 0.597 | 0.201 |
| Q60597 | 2-oxoglutarate dehydrogenase, mitochondrial OS=Mus musculus GN=Ogdh PE=1 SV=3 | 0.602 | 0.208 |
| Q91V61 | Sideroflexin-3 OS=Mus musculus GN=Sfxn3 PE=1 SV=1 | 0.603 | 0.210 |
| P23116 | Eukaryotic translation initiation factor 3 subunit A OS=Mus musculus GN=Eif3a PE=1 SV=5 | 0.603 | 0.210 |
| Q02013 | Aquaporin-1 OS=Mus musculus GN=Aqp1 PE=1 SV=3 | 0.604 | 0.211 |
| Q3UN19 | Tropomodulin 4 OS=Mus musculus GN=Tmod4 PE=1 SV=1 | 0.605 | 0.212 |
| Q80XB4 | Nebulin-related-anchoring protein OS=Mus musculus GN=Nrap PE=1 SV=3 | 0.605 | 0.213 |
| P80316 | T-complex protein 1 subunit epsilon OS=Mus musculus GN=Cct5 PE=1 SV=1 | 0.608 | 0.217 |
| Q9CPQ8 | ATP synthase subunit g, mitochondrial OS=Mus musculus GN=Atp5l PE=1 SV=1 | 0.608 | 0.217 |
| P67778 | Prohibitin OS=Mus musculus GN=Phb PE=1 SV=1 | 0.608 | 0.217 |
| E9QQ96 | Obscurin OS=Mus musculus GN=Obscn PE=1 SV=2 | 0.61 | 0.220 |
| O35226-2 | Isoform Rpn10B of 26S proteasome non-ATPase regulatory subunit 4 OS=Mus musculus GN=Psmd4 | 0.611 | 0.221 |
| Q3UKW2 | Calmodulin OS=Mus musculus GN=Calm1 PE=1 SV=1 | 0.612 | 0.222 |
| Q8BK84 | Dual specificity phosphatase DUPD1 OS=Mus musculus GN=Dupd1 PE=2 SV=1 | 0.614 | 0.225 |
| P62835 | Ras-related protein Rap-1A OS=Mus musculus GN=Rap1a PE=1 SV=1 | 0.614 | 0.226 |
| P14206 | 40S ribosomal protein SA OS=Mus musculus GN=Rpsa PE=1 SV=4 | 0.616 | 0.229 |
| Q8VCX5-2 | Isoform 2 of Calcium uptake protein 1, mitochondrial OS=Mus musculus GN=Micu1 | 0.619 | 0.233 |
| H3BJQ7 | Peroxiredoxin-5, mitochondrial OS=Mus musculus GN=Prdx5 PE=1 SV=1 | 0.619 | 0.233 |
| P97457 | Myosin regulatory light chain 2, skeletal muscle isoform OS=Mus musculus GN=Mylpf PE=1 SV=3 | 0.621 | 0.236 |
| A2AMM0 | Muscle-related coiled-coil protein OS=Mus musculus GN=Murc PE=1 SV=1 | 0.621 | 0.236 |
| Q8BVQ9 | 26S protease regulatory subunit 7 OS=Mus musculus GN=Psmc2 PE=1 SV=1 | 0.622 | 0.237 |
| P43023 | Cytochrome c oxidase subunit 6A2, mitochondrial OS=Mus musculus GN=Cox6a2 PE=1 SV=2 | 0.625 | 0.242 |
| Q9Z2I8 | Succinyl-CoA ligase [GDP-forming] subunit beta, mitochondrial OS=Mus musculus GN=Suclg2 PE=1 SV=3 | 0.626 | 0.243 |
| Q8K4F5 | Protein ABHD11 OS=Mus musculus GN=Abhd11 PE=1 SV=1 | 0.628 | 0.246 |
| A0A0R4J0Z1 | Protein disulfide-isomerase A4 OS=Mus musculus GN=Pdia4 PE=1 SV=1 | 0.63 | 0.249 |
| Q91WD5 | NADH dehydrogenase [ubiquinone] iron-sulfur protein 2, mitochondrial OS=Mus musculus GN=Ndufs2 PE=1 SV=1 | 0.631 | 0.251 |
| Q99MR9 | Protein phosphatase 1 regulatory subunit 3A OS=Mus musculus GN=Ppp1r3a PE=1 SV=2 | 0.633 | 0.254 |
| P20801 | Troponin C, skeletal muscle OS=Mus musculus GN=Tnnc2 PE=1 SV=2 | 0.633 | 0.254 |
| Q9DCZ4-3 | Isoform 3 of MICOS complex subunit Mic26 OS=Mus musculus GN=Apoo | 0.633 | 0.254 |
| P49817 | Caveolin-1 OS=Mus musculus GN=Cav1 PE=1 SV=1 | 0.634 | 0.255 |
| Q9JII6 | Alcohol dehydrogenase [NADP(+)] OS=Mus musculus GN=Akr1a1 PE=1 SV=3 | 0.637 | 0.260 |
| Q99P72-1 | Isoform 3 of Reticulon-4 OS=Mus musculus GN=Rtn4 | 0.637 | 0.260 |
| Q99JB8 | Protein kinase C and casein kinase II substrate protein 3 OS=Mus musculus GN=Pacsin3 PE=1 SV=1 | 0.638 | 0.261 |
| B2RTM0 | Histone H4 OS=Mus musculus GN=Hist2h4 PE=1 SV=1 | 0.639 | 0.263 |
| Q07417 | Short-chain specific acyl-CoA dehydrogenase, mitochondrial OS=Mus musculus GN=Acads PE=1 SV=2 | 0.639 | 0.264 |
| Q3U2G2 | Heat shock 70 kDa protein 4 OS=Mus musculus GN=Hspa4 PE=1 SV=1 | 0.64 | 0.266 |
| G3UWS1 | Protein Neb (Fragment) OS=Mus musculus GN=Neb PE=1 SV=1 | 0.641 | 0.267 |
| E9QN70 | Laminin subunit beta-1 OS=Mus musculus GN=Lamb1 PE=1 SV=1 | 0.641 | 0.267 |
| Q9CQX8 | 28S ribosomal protein S36, mitochondrial OS=Mus musculus GN=Mrps36 PE=1 SV=1 | 0.642 | 0.268 |
| Q64691 | Calpain-3 OS=Mus musculus GN=Capn3 PE=2 SV=2 | 0.643 | 0.270 |
| Q80X90 | Filamin-B OS=Mus musculus GN=Flnb PE=1 SV=3 | 0.646 | 0.274 |
| E9QPD7 | Pyruvate carboxylase OS=Mus musculus GN=Pcx PE=1 SV=1 | 0.647 | 0.276 |
| O88844 | Isocitrate dehydrogenase [NADP] cytoplasmic OS=Mus musculus GN=Idh1 PE=1 SV=2 | 0.648 | 0.278 |
| E9PW66 | Nucleosome assembly protein 1-like 1 OS=Mus musculus GN=Nap1l1 PE=1 SV=1 | 0.65 | 0.281 |
| P82198 | Transforming growth factor-beta-induced protein ig-h3 OS=Mus musculus GN=Tgfbi PE=1 SV=1 | 0.651 | 0.282 |
| E9QK82 | Myelin protein P0 OS=Mus musculus GN=Mpz PE=1 SV=1 | 0.652 | 0.284 |
| P00920 | Carbonic anhydrase 2 OS=Mus musculus GN=Ca2 PE=1 SV=4 | 0.652 | 0.285 |
| P51885 | Lumican OS=Mus musculus GN=Lum PE=1 SV=2 | 0.655 | 0.289 |
| Q8C266 | Ras-related protein Rab-5C OS=Mus musculus GN=Rab5c PE=1 SV=1 | 0.655 | 0.289 |
| A2AE89 | Glutathione S-transferase Mu 1 OS=Mus musculus GN=Gstm1 PE=1 SV=2 | 0.656 | 0.291 |
| Q9R0P5 | Destrin OS=Mus musculus GN=Dstn PE=1 SV=3 | 0.656 | 0.291 |
| Q8CGK3 | Lon protease homolog, mitochondrial OS=Mus musculus GN=Lonp1 PE=1 SV=2 | 0.657 | 0.292 |
| P43276 | Histone H1.5 OS=Mus musculus GN=Hist1h1b PE=1 SV=2 | 0.658 | 0.295 |
| E9QLJ0 | Cardiomyopathy-associated protein 5 OS=Mus musculus GN=Cmya5 PE=1 SV=1 | 0.66 | 0.297 |
| B2RTL5 | Aldehyde dehydrogenase family 1, subfamily A7 OS=Mus musculus GN=Aldh1a7 PE=1 SV=1 | 0.66 | 0.298 |
| J3QMG5 | Protein Gm5786 OS=Mus musculus GN=Gm5786 PE=3 SV=1 | 0.661 | 0.299 |
| P61161 | Actin-related protein 2 OS=Mus musculus GN=Actr2 PE=1 SV=1 | 0.662 | 0.301 |
| Q8BH80 | Vesicle-associated membrane protein, associated protein B and C OS=Mus musculus GN=Vapb PE=1 SV=1 | 0.663 | 0.303 |
| B1B1A8 | Myosin light chain kinase, smooth muscle OS=Mus musculus GN=Mylk PE=1 SV=1 | 0.665 | 0.305 |
| Q4FK74 | ATP synthase subunit delta, mitochondrial OS=Mus musculus GN=Atp5d PE=1 SV=1 | 0.665 | 0.306 |
| Q8VCT4 | Carboxylesterase 1D OS=Mus musculus GN=Ces1d PE=1 SV=1 | 0.666 | 0.307 |
| P14094 | Sodium/potassium-transporting ATPase subunit beta-1 OS=Mus musculus GN=Atp1b1 PE=1 SV=1 | 0.669 | 0.313 |
| Q5I0W0 | ATP synthase F(0) complex subunit B1, mitochondrial OS=Mus musculus GN=Atp5f1 PE=1 SV=1 | 0.672 | 0.318 |
| Q9Z2I0 | LETM1 and EF-hand domain-containing protein 1, mitochondrial OS=Mus musculus GN=Letm1 PE=1 SV=1 | 0.673 | 0.318 |
| P51667 | Myosin regulatory light chain 2, ventricular/cardiac muscle isoform OS=Mus musculus GN=Myl2 PE=1 SV=3 | 0.673 | 0.319 |
| P53026 | 60S ribosomal protein L10a OS=Mus musculus GN=Rpl10a PE=1 SV=3 | 0.673 | 0.319 |
| D3YVS1 | Smoothelin OS=Mus musculus GN=Smtn PE=1 SV=1 | 0.673 | 0.319 |
| P27546 | Microtubule-associated protein 4 OS=Mus musculus GN=Map4 PE=1 SV=3 | 0.675 | 0.322 |
| Q3UZG4 | Aminoacyl tRNA synthase complex-interacting multifunctional protein 1 OS=Mus musculus GN=Aimp1 PE=1 SV=1 | 0.676 | 0.323 |
| A2AFQ2 | 3-hydroxyacyl-CoA dehydrogenase type-2 OS=Mus musculus GN=Hsd17b10 PE=1 SV=1 | 0.676 | 0.324 |
| E9PZC3 | Flavin reductase (NADPH) OS=Mus musculus GN=Blvrb PE=1 SV=1 | 0.677 | 0.326 |
| Q5SX53 | Mitochondrial 2-oxoglutarate/malate carrier protein OS=Mus musculus GN=Slc25a11 PE=1 SV=1 | 0.677 | 0.326 |
| Q02789 | Voltage-dependent L-type calcium channel subunit alpha-1S OS=Mus musculus GN=Cacna1s PE=1 SV=2 | 0.68 | 0.330 |
| E9QA15 | Protein Cald1 OS=Mus musculus GN=Cald1 PE=1 SV=1 | 0.681 | 0.332 |
| Q8BZA9 | Fructose-2,6-bisphosphatase TIGAR OS=Mus musculus GN=Tigar PE=1 SV=1 | 0.683 | 0.335 |
| Q9Z2Z6 | Mitochondrial carnitine/acylcarnitine carrier protein OS=Mus musculus GN=Slc25a20 PE=1 SV=1 | 0.687 | 0.343 |
| Q08642 | Protein-arginine deiminase type-2 OS=Mus musculus GN=Padi2 PE=1 SV=2 | 0.687 | 0.343 |
| Q9WUZ5 | Troponin I, slow skeletal muscle OS=Mus musculus GN=Tnni1 PE=1 SV=3 | 0.688 | 0.345 |
| Q9D6K8 | FUN14 domain-containing protein 2 OS=Mus musculus GN=Fundc2 PE=1 SV=1 | 0.691 | 0.349 |
| P70333 | Heterogeneous nuclear ribonucleoprotein H2 OS=Mus musculus GN=Hnrnph2 PE=1 SV=1 | 0.693 | 0.353 |
| Q9DBG3-2 | Isoform 2 of AP-2 complex subunit beta OS=Mus musculus GN=Ap2b1 | 0.696 | 0.358 |
| Q9QZQ8 | Core histone macro-H2A.1 OS=Mus musculus GN=H2afy PE=1 SV=3 | 0.696 | 0.358 |
| Q9DBS1 | Transmembrane protein 43 OS=Mus musculus GN=Tmem43 PE=1 SV=1 | 0.696 | 0.359 |
| Q544X6 | Ferrochelatase OS=Mus musculus GN=Fech PE=1 SV=1 | 0.697 | 0.361 |
| O09061 | Proteasome subunit beta type-1 OS=Mus musculus GN=Psmb1 PE=1 SV=1 | 0.701 | 0.367 |
| Q9DB73 | NADH-cytochrome b5 reductase 1 OS=Mus musculus GN=Cyb5r1 PE=1 SV=1 | 0.702 | 0.369 |
| P14733 | Lamin-B1 OS=Mus musculus GN=Lmnb1 PE=1 SV=3 | 0.703 | 0.371 |
| Q91VM9 | Inorganic pyrophosphatase 2, mitochondrial OS=Mus musculus GN=Ppa2 PE=1 SV=1 | 0.703 | 0.372 |
| Q99M71 | Mammalian ependymin-related protein 1 OS=Mus musculus GN=Epdr1 PE=1 SV=1 | 0.705 | 0.374 |
| E9Q800 | MICOS complex subunit MIC60 OS=Mus musculus GN=Immt PE=1 SV=1 | 0.706 | 0.376 |
| Q8BJS4 | SUN domain-containing protein 2 OS=Mus musculus GN=Sun2 PE=1 SV=3 | 0.707 | 0.379 |
| Q6P5E4 | UDP-glucose:glycoprotein glucosyltransferase 1 OS=Mus musculus GN=Uggt1 PE=1 SV=4 | 0.707 | 0.379 |
| Q8BKZ9 | Pyruvate dehydrogenase protein X component, mitochondrial OS=Mus musculus GN=Pdhx PE=1 SV=1 | 0.707 | 0.379 |
| O09165 | Calsequestrin-1 OS=Mus musculus GN=Casq1 PE=1 SV=3 | 0.708 | 0.380 |
| B1AX58 | Plastin-3 OS=Mus musculus GN=Pls3 PE=1 SV=1 | 0.711 | 0.385 |
| P15089 | Mast cell carboxypeptidase A OS=Mus musculus GN=Cpa3 PE=2 SV=1 | 0.712 | 0.388 |
| Q99PT1 | Rho GDP-dissociation inhibitor 1 OS=Mus musculus GN=Arhgdia PE=1 SV=3 | 0.718 | 0.397 |
| Q61781 | Keratin, type I cytoskeletal 14 OS=Mus musculus GN=Krt14 PE=1 SV=2 | 0.719 | 0.400 |
| A2AE45 | Synaptophysin-like protein 2 (Fragment) OS=Mus musculus GN=Sypl2 PE=1 SV=1 | 0.72 | 0.402 |
| Q62507 | Cochlin OS=Mus musculus GN=Coch PE=1 SV=2 | 0.72 | 0.402 |
| Q7TMG8 | Glioblastoma amplified sequence OS=Mus musculus GN=Gbas PE=1 SV=1 | 0.721 | 0.404 |
| P51125 | Calpastatin OS=Mus musculus GN=Cast PE=1 SV=2 | 0.723 | 0.407 |
| Q9DCS9 | NADH dehydrogenase [ubiquinone] 1 beta subcomplex subunit 10 OS=Mus musculus GN=Ndufb10 PE=1 SV=3 | 0.723 | 0.407 |
| Q9D2G2 | Dihydrolipoyllysine-residue succinyltransferase component of 2-oxoglutarate dehydrogenase complex, mitochondrial OS=Mus musculus GN=Dlst PE=1 SV=1 | 0.727 | 0.415 |
| P35385 | Heat shock protein beta-7 OS=Mus musculus GN=Hspb7 PE=1 SV=3 | 0.728 | 0.417 |
| A2AI87 | Phosphorylase b kinase regulatory subunit alpha, skeletal muscle isoform OS=Mus musculus GN=Phka1 PE=1 SV=1 | 0.731 | 0.422 |
| Q3UER8 | Fibrinogen gamma chain OS=Mus musculus GN=Fgg PE=1 SV=1 | 0.732 | 0.424 |
| Q9D023 | Mitochondrial pyruvate carrier 2 OS=Mus musculus GN=Mpc2 PE=1 SV=1 | 0.732 | 0.424 |
| P21614 | Vitamin D-binding protein OS=Mus musculus GN=Gc PE=1 SV=2 | 0.733 | 0.425 |
| E9PV24 | Fibrinogen alpha chain OS=Mus musculus GN=Fga PE=1 SV=1 | 0.733 | 0.426 |
| P84228 | Histone H3.2 OS=Mus musculus GN=Hist1h3b PE=1 SV=2 | 0.734 | 0.427 |
| A6H584 | Collagen alpha-5(VI) chain OS=Mus musculus GN=Col6a5 PE=1 SV=4 | 0.736 | 0.431 |
| Q9Z2Y8 | Proline synthase co-transcribed bacterial homolog protein OS=Mus musculus GN=Prosc PE=1 SV=1 | 0.737 | 0.434 |
| Q3TMP8 | Trimeric intracellular cation channel type A OS=Mus musculus GN=Tmem38a PE=1 SV=2 | 0.738 | 0.434 |
| O08749 | Dihydrolipoyl dehydrogenase, mitochondrial OS=Mus musculus GN=Dld PE=1 SV=2 | 0.739 | 0.438 |
| P60766 | Cell division control protein 42 homolog OS=Mus musculus GN=Cdc42 PE=1 SV=2 | 0.74 | 0.439 |
| A0A0R4IZY0 | Thimet oligopeptidase OS=Mus musculus GN=Thop1 PE=1 SV=1 | 0.742 | 0.442 |
| Q61768 | Kinesin-1 heavy chain OS=Mus musculus GN=Kif5b PE=1 SV=3 | 0.743 | 0.444 |
| Q99LC3 | NADH dehydrogenase [ubiquinone] 1 alpha subcomplex subunit 10, mitochondrial OS=Mus musculus GN=Ndufa10 PE=1 SV=1 | 0.744 | 0.445 |
| Q60847 | Collagen alpha-1(XII) chain OS=Mus musculus GN=Col12a1 PE=2 SV=3 | 0.746 | 0.450 |
| P56399 | Ubiquitin carboxyl-terminal hydrolase 5 OS=Mus musculus GN=Usp5 PE=1 SV=1 | 0.746 | 0.450 |
| Q9CZ30 | Obg-like ATPase 1 OS=Mus musculus GN=Ola1 PE=1 SV=1 | 0.748 | 0.454 |
| Q8BGH2 | Sorting and assembly machinery component 50 homolog OS=Mus musculus GN=Samm50 PE=1 SV=1 | 0.749 | 0.456 |
| Q9CQC7 | NADH dehydrogenase [ubiquinone] 1 beta subcomplex subunit 4 OS=Mus musculus GN=Ndufb4 PE=1 SV=3 | 0.75 | 0.457 |
| Q64310 | Surfeit locus protein 4 OS=Mus musculus GN=Surf4 PE=1 SV=1 | 0.752 | 0.461 |
| E9QPE7 | Myosin-11 OS=Mus musculus GN=Myh11 PE=1 SV=1 | 0.754 | 0.466 |
| P42125 | Enoyl-CoA delta isomerase 1, mitochondrial OS=Mus musculus GN=Eci1 PE=1 SV=2 | 0.755 | 0.467 |
| P52825 | Carnitine O-palmitoyltransferase 2, mitochondrial OS=Mus musculus GN=Cpt2 PE=1 SV=2 | 0.762 | 0.480 |
| O70251 | Elongation factor 1-beta OS=Mus musculus GN=Eef1b PE=1 SV=5 | 0.763 | 0.482 |
| Q8VDQ1 | Prostaglandin reductase 2 OS=Mus musculus GN=Ptgr2 PE=1 SV=2 | 0.764 | 0.485 |
| O70622-2 | Isoform 2 of Reticulon-2 OS=Mus musculus GN=Rtn2 | 0.769 | 0.494 |
| Q497E9 | 40S ribosomal protein S8 OS=Mus musculus GN=Rps8 PE=1 SV=1 | 0.769 | 0.494 |
| Q8BH64 | EH domain-containing protein 2 OS=Mus musculus GN=Ehd2 PE=1 SV=1 | 0.772 | 0.499 |
| Q3V100 | Mannose-6-phosphate isomerase OS=Mus musculus GN=Mpi PE=1 SV=1 | 0.772 | 0.500 |
| P50396 | Rab GDP dissociation inhibitor alpha OS=Mus musculus GN=Gdi1 PE=1 SV=3 | 0.774 | 0.503 |
| A2AUC9 | Kelch-like protein 41 OS=Mus musculus GN=Klhl41 PE=1 SV=1 | 0.775 | 0.505 |
| O55026 | Ectonucleoside triphosphate diphosphohydrolase 2 OS=Mus musculus GN=Entpd2 PE=1 SV=2 | 0.78 | 0.515 |
| P54822 | Adenylosuccinate lyase OS=Mus musculus GN=Adsl PE=1 SV=2 | 0.782 | 0.519 |
| Q4KL26 | Voltage-dependent calcium channel gamma subunit OS=Mus musculus GN=Cacng1 PE=2 SV=1 | 0.784 | 0.522 |
| Q8BH61 | Coagulation factor XIII A chain OS=Mus musculus GN=F13a1 PE=1 SV=3 | 0.784 | 0.523 |
| Q60932 | Voltage-dependent anion-selective channel protein 1 OS=Mus musculus GN=Vdac1 PE=1 SV=3 | 0.786 | 0.526 |
| O70400 | PDZ and LIM domain protein 1 OS=Mus musculus GN=Pdlim1 PE=1 SV=4 | 0.787 | 0.529 |
| A1BN54 | Alpha actinin 1a OS=Mus musculus GN=Actn1 PE=1 SV=1 | 0.787 | 0.529 |
| O70318 | Band 4.1-like protein 2 OS=Mus musculus GN=Epb41l2 PE=1 SV=2 | 0.789 | 0.534 |
| Q9CQ69 | Cytochrome b-c1 complex subunit 8 OS=Mus musculus GN=Uqcrq PE=1 SV=3 | 0.79 | 0.534 |
| Q8K1M3 | Protein kinase, cAMP dependent regulatory, type II alpha OS=Mus musculus GN=Prkar2a PE=1 SV=1 | 0.794 | 0.542 |
| Q8BZ71 | SH3 and cysteine-rich domain-containing protein 3 OS=Mus musculus GN=Stac3 PE=2 SV=1 | 0.794 | 0.542 |
| P10639 | Thioredoxin OS=Mus musculus GN=Txn PE=1 SV=3 | 0.795 | 0.543 |
| B2RWH3 | Histone H2A OS=Mus musculus GN=Hist2h2aa1 PE=2 SV=1 | 0.795 | 0.545 |
| Q9CPP6 | NADH dehydrogenase [ubiquinone] 1 alpha subcomplex subunit 5 OS=Mus musculus GN=Ndufa5 PE=1 SV=3 | 0.798 | 0.550 |
| E9PZD8 | Ceruloplasmin OS=Mus musculus GN=Cp PE=1 SV=1 | 0.799 | 0.551 |
| P28653 | Biglycan OS=Mus musculus GN=Bgn PE=1 SV=1 | 0.799 | 0.552 |
| Q8BMF4 | Dihydrolipoyllysine-residue acetyltransferase component of pyruvate dehydrogenase complex, mitochondrial OS=Mus musculus GN=Dlat PE=1 SV=2 | 0.799 | 0.552 |
| Q9CYT6 | Adenylyl cyclase-associated protein 2 OS=Mus musculus GN=Cap2 PE=1 SV=1 | 0.799 | 0.552 |
| Q921T2 | Torsin-1A-interacting protein 1 OS=Mus musculus GN=Tor1aip1 PE=1 SV=3 | 0.8 | 0.554 |
| P16125 | L-lactate dehydrogenase B chain OS=Mus musculus GN=Ldhb PE=1 SV=2 | 0.804 | 0.563 |
| Q6NZJ6 | Eukaryotic translation initiation factor 4 gamma 1 OS=Mus musculus GN=Eif4g1 PE=1 SV=1 | 0.805 | 0.564 |
| Q9JLV1 | BAG family molecular chaperone regulator 3 OS=Mus musculus GN=Bag3 PE=1 SV=2 | 0.806 | 0.566 |
| Q58EW0 | 60S ribosomal protein L18 OS=Mus musculus GN=Rpl18 PE=1 SV=1 | 0.806 | 0.566 |
| P42669 | Transcriptional activator protein Pur-alpha OS=Mus musculus GN=Pura PE=1 SV=1 | 0.806 | 0.566 |
| Q9CRB6 | Tubulin polymerization-promoting protein family member 3 OS=Mus musculus GN=Tppp3 PE=1 SV=1 | 0.807 | 0.568 |
| Q5BL18 | Matrin 3 OS=Mus musculus GN=Matr3 PE=1 SV=1 | 0.807 | 0.568 |
| Q61990 | Poly(rC)-binding protein 2 OS=Mus musculus GN=Pcbp2 PE=1 SV=1 | 0.807 | 0.568 |
| P07934 | Phosphorylase b kinase gamma catalytic chain, skeletal muscle/heart isoform OS=Mus musculus GN=Phkg1 PE=1 SV=3 | 0.809 | 0.571 |
| Q9QUM9 | Proteasome subunit alpha type-6 OS=Mus musculus GN=Psma6 PE=1 SV=1 | 0.81 | 0.573 |
| D3YU50 | Protein Mybpc1 OS=Mus musculus GN=Mybpc1 PE=1 SV=1 | 0.814 | 0.581 |
| P45376 | Aldose reductase OS=Mus musculus GN=Akr1b1 PE=1 SV=3 | 0.814 | 0.583 |
| P47738 | Aldehyde dehydrogenase, mitochondrial OS=Mus musculus GN=Aldh2 PE=1 SV=1 | 0.818 | 0.589 |
| Q3TVI8 | Pre-B-cell leukemia transcription factor-interacting protein 1 OS=Mus musculus GN=Pbxip1 PE=1 SV=2 | 0.818 | 0.591 |
| Q9WV55 | Vesicle-associated membrane protein-associated protein A OS=Mus musculus GN=Vapa PE=1 SV=2 | 0.819 | 0.592 |
| Q9D172 | ES1 protein homolog, mitochondrial OS=Mus musculus GN=D10Jhu81e PE=1 SV=1 | 0.821 | 0.595 |
| A0A087WS16 | Protein Col6a3 OS=Mus musculus GN=Col6a3 PE=1 SV=1 | 0.821 | 0.595 |
| Q8BMS1 | Trifunctional enzyme subunit alpha, mitochondrial OS=Mus musculus GN=Hadha PE=1 SV=1 | 0.821 | 0.597 |
| A2CEK3 | Phosphoglucomutase-2 OS=Mus musculus GN=Pgm2 PE=1 SV=1 | 0.822 | 0.598 |
| P26883 | Peptidyl-prolyl cis-trans isomerase FKBP1A OS=Mus musculus GN=Fkbp1a PE=1 SV=2 | 0.823 | 0.599 |
| Q61598 | Rab GDP dissociation inhibitor beta OS=Mus musculus GN=Gdi2 PE=1 SV=1 | 0.823 | 0.599 |
| Q8K1Z0 | Ubiquinone biosynthesis protein COQ9, mitochondrial OS=Mus musculus GN=Coq9 PE=1 SV=1 | 0.823 | 0.599 |
| K3W4S6 | Glycogenin-1 OS=Mus musculus GN=Gyg PE=1 SV=1 | 0.823 | 0.600 |
| P62852 | 40S ribosomal protein S25 OS=Mus musculus GN=Rps25 PE=1 SV=1 | 0.824 | 0.601 |
| P23953 | Carboxylesterase 1C OS=Mus musculus GN=Ces1c PE=1 SV=4 | 0.826 | 0.605 |
| O55143 | Sarcoplasmic/endoplasmic reticulum calcium ATPase 2 OS=Mus musculus GN=Atp2a2 PE=1 SV=2 | 0.826 | 0.606 |
| Q8BTM8 | Filamin-A OS=Mus musculus GN=Flna PE=1 SV=5 | 0.827 | 0.607 |
| Q9CQM9 | Glutaredoxin-3 OS=Mus musculus GN=Glrx3 PE=1 SV=1 | 0.827 | 0.607 |
| O08911 | Mitogen-activated protein kinase 12 OS=Mus musculus GN=Mapk12 PE=1 SV=1 | 0.827 | 0.608 |
| Q542X7 | Chaperonin subunit 2 (Beta), isoform CRA_a OS=Mus musculus GN=Cct2 PE=1 SV=1 | 0.828 | 0.609 |
| Q8BXC0 | Prostacyclin synthase OS=Mus musculus GN=Ptgis PE=1 SV=1 | 0.828 | 0.609 |
| Q9Z2U1 | Proteasome subunit alpha type-5 OS=Mus musculus GN=Psma5 PE=1 SV=1 | 0.828 | 0.610 |
| Q9CZD3 | Glycine--tRNA ligase OS=Mus musculus GN=Gars PE=1 SV=1 | 0.829 | 0.611 |
| Q9JI91 | Alpha-actinin-2 OS=Mus musculus GN=Actn2 PE=1 SV=2 | 0.83 | 0.614 |
| P12970 | 60S ribosomal protein L7a OS=Mus musculus GN=Rpl7a PE=1 SV=2 | 0.831 | 0.615 |
| G5E8J6 | Histidine rich calcium binding protein, isoform CRA_a OS=Mus musculus GN=Hrc PE=1 SV=1 | 0.833 | 0.619 |
| Q9WV35 | C->U-editing enzyme APOBEC-2 OS=Mus musculus GN=Apobec2 PE=1 SV=1 | 0.836 | 0.626 |
| Q7TQ48 | Sarcalumenin OS=Mus musculus GN=Srl PE=1 SV=1 | 0.837 | 0.627 |
| Q0VGU0 | GTP-binding protein SAR1b OS=Mus musculus GN=Sar1b PE=1 SV=1 | 0.837 | 0.628 |
| Q99MR8 | Methylcrotonoyl-CoA carboxylase subunit alpha, mitochondrial OS=Mus musculus GN=Mccc1 PE=1 SV=2 | 0.839 | 0.631 |
| Q3ULU3 | Branched-chain-amino-acid aminotransferase OS=Mus musculus GN=Bcat2 PE=1 SV=1 | 0.84 | 0.633 |
| Q9DBL1 | Short/branched chain specific acyl-CoA dehydrogenase, mitochondrial OS=Mus musculus GN=Acadsb PE=1 SV=1 | 0.841 | 0.636 |
| P61202 | COP9 signalosome complex subunit 2 OS=Mus musculus GN=Cops2 PE=1 SV=1 | 0.842 | 0.637 |
| Q99JY9 | Actin-related protein 3 OS=Mus musculus GN=Actr3 PE=1 SV=3 | 0.843 | 0.639 |
| P07356 | Annexin A2 OS=Mus musculus GN=Anxa2 PE=1 SV=2 | 0.843 | 0.640 |
| Q8BH59 | Calcium-binding mitochondrial carrier protein Aralar1 OS=Mus musculus GN=Slc25a12 PE=1 SV=1 | 0.844 | 0.640 |
| Q5SQB7 | MCG68069 OS=Mus musculus GN=Npm1 PE=1 SV=1 | 0.845 | 0.644 |
| Q9QYJ0 | DnaJ homolog subfamily A member 2 OS=Mus musculus GN=Dnaja2 PE=1 SV=1 | 0.845 | 0.644 |
| P10107 | Annexin A1 OS=Mus musculus GN=Anxa1 PE=1 SV=2 | 0.846 | 0.645 |
| Q3ULT2 | Actinin alpha 4 OS=Mus musculus GN=Actn4 PE=1 SV=1 | 0.846 | 0.645 |
| P48758 | Carbonyl reductase [NADPH] 1 OS=Mus musculus GN=Cbr1 PE=1 SV=3 | 0.851 | 0.654 |
| Q6PB66 | Leucine-rich PPR motif-containing protein, mitochondrial OS=Mus musculus GN=Lrpprc PE=1 SV=2 | 0.851 | 0.654 |
| Q62433 | Protein NDRG1 OS=Mus musculus GN=Ndrg1 PE=1 SV=1 | 0.852 | 0.657 |
| Q9QYG0-2 | Isoform 2 of Protein NDRG2 OS=Mus musculus GN=Ndrg2 | 0.852 | 0.657 |
| G5E924 | Heterogeneous nuclear ribonucleoprotein L (Fragment) OS=Mus musculus GN=Hnrnpl PE=1 SV=1 | 0.853 | 0.658 |
| Q9CR68 | Cytochrome b-c1 complex subunit Rieske, mitochondrial OS=Mus musculus GN=Uqcrfs1 PE=1 SV=1 | 0.853 | 0.660 |
| P10922 | Histone H1.0 OS=Mus musculus GN=H1f0 PE=2 SV=4 | 0.855 | 0.663 |
| E9Q3H6 | Protein Coq10a OS=Mus musculus GN=Coq10a PE=1 SV=1 | 0.856 | 0.665 |
| Q61838 | Pregnancy zone protein OS=Mus musculus GN=Pzp PE=1 SV=3 | 0.856 | 0.666 |
| Q62425 | Cytochrome c oxidase subunit NDUFA4 OS=Mus musculus GN=Ndufa4 PE=1 SV=2 | 0.857 | 0.666 |
| P05201 | Aspartate aminotransferase, cytoplasmic OS=Mus musculus GN=Got1 PE=1 SV=3 | 0.857 | 0.668 |
| Q9R1P1 | Proteasome subunit beta type-3 OS=Mus musculus GN=Psmb3 PE=1 SV=1 | 0.858 | 0.669 |
| Q8BH95 | Enoyl-CoA hydratase, mitochondrial OS=Mus musculus GN=Echs1 PE=1 SV=1 | 0.858 | 0.669 |
| G3UW82 | MCG140437, isoform CRA_d OS=Mus musculus GN=Myh2 PE=1 SV=1 | 0.86 | 0.672 |
| P49722 | Proteasome subunit alpha type-2 OS=Mus musculus GN=Psma2 PE=1 SV=3 | 0.862 | 0.677 |
| Q9DBB8 | Trans-1,2-dihydrobenzene-1,2-diol dehydrogenase OS=Mus musculus GN=Dhdh PE=1 SV=1 | 0.862 | 0.678 |
| Q1XH17 | Tripartite motif-containing protein 72 OS=Mus musculus GN=Trim72 PE=1 SV=1 | 0.865 | 0.684 |
| B9EHN0 | Ubiquitin-activating enzyme E1, Chr X OS=Mus musculus GN=Uba1 PE=1 SV=1 | 0.867 | 0.686 |
| Q5EBG6 | Heat shock protein beta-6 OS=Mus musculus GN=Hspb6 PE=1 SV=1 | 0.867 | 0.686 |
| E9PZQ0 | Ryanodine receptor 1 OS=Mus musculus GN=Ryr1 PE=1 SV=1 | 0.867 | 0.688 |
| J3QMG3 | Voltage-dependent anion-selective channel protein 3 OS=Mus musculus GN=Vdac3 PE=1 SV=1 | 0.87 | 0.692 |
| P12787 | Cytochrome c oxidase subunit 5A, mitochondrial OS=Mus musculus GN=Cox5a PE=1 SV=2 | 0.871 | 0.695 |
| P80313 | T-complex protein 1 subunit eta OS=Mus musculus GN=Cct7 PE=1 SV=1 | 0.872 | 0.698 |
| O08532 | Voltage-dependent calcium channel subunit alpha-2/delta-1 OS=Mus musculus GN=Cacna2d1 PE=1 SV=1 | 0.873 | 0.699 |
| P35293 | Ras-related protein Rab-18 OS=Mus musculus GN=Rab18 PE=1 SV=2 | 0.875 | 0.703 |
| Q9R0Q3 | Transmembrane emp24 domain-containing protein 2 OS=Mus musculus GN=Tmed2 PE=1 SV=1 | 0.875 | 0.703 |
| A2AAJ9 | Obscurin OS=Mus musculus GN=Obscn PE=1 SV=2 | 0.875 | 0.704 |
| Q5SX40 | Myosin-1 OS=Mus musculus GN=Myh1 PE=1 SV=1 | 0.877 | 0.706 |
| E9Q3X0 | Major vault protein OS=Mus musculus GN=Mvp PE=1 SV=1 | 0.877 | 0.706 |
| K3W4M4 | ATP-dependent (S)-NAD(P)H-hydrate dehydratase OS=Mus musculus GN=Carkd PE=1 SV=1 | 0.877 | 0.707 |
| P17563 | Selenium-binding protein 1 OS=Mus musculus GN=Selenbp1 PE=1 SV=2 | 0.877 | 0.707 |
| Q99L13 | 3-hydroxyisobutyrate dehydrogenase, mitochondrial OS=Mus musculus GN=Hibadh PE=1 SV=1 | 0.878 | 0.709 |
| Q8C0M9 | Isoaspartyl peptidase/L-asparaginase OS=Mus musculus GN=Asrgl1 PE=1 SV=1 | 0.879 | 0.711 |
| Q8VDD5 | Myosin-9 OS=Mus musculus GN=Myh9 PE=1 SV=4 | 0.88 | 0.712 |
| Q7TMK9 | Heterogeneous nuclear ribonucleoprotein Q OS=Mus musculus GN=Syncrip PE=1 SV=2 | 0.881 | 0.714 |
| A2AL78 | Aspartyl/asparaginyl beta-hydroxylase OS=Mus musculus GN=Asph PE=1 SV=1 | 0.881 | 0.714 |
| Q3UN88 | Mast cell protease 4 OS=Mus musculus GN=Mcpt4 PE=1 SV=1 | 0.881 | 0.714 |
| P63082 | V-type proton ATPase 16 kDa proteolipid subunit OS=Mus musculus GN=Atp6v0c PE=1 SV=1 | 0.881 | 0.715 |
| Q64152 | Transcription factor BTF3 OS=Mus musculus GN=Btf3 PE=1 SV=3 | 0.882 | 0.717 |
| Q99MN9 | Propionyl-CoA carboxylase beta chain, mitochondrial OS=Mus musculus GN=Pccb PE=1 SV=2 | 0.883 | 0.718 |
| Q9D2M8 | Ubiquitin-conjugating enzyme E2 variant 2 OS=Mus musculus GN=Ube2v2 PE=1 SV=4 | 0.884 | 0.720 |
| Q9QYG0 | Protein NDRG2 OS=Mus musculus GN=Ndrg2 PE=1 SV=1 | 0.884 | 0.720 |
| P63038 | 60 kDa heat shock protein, mitochondrial OS=Mus musculus GN=Hspd1 PE=1 SV=1 | 0.885 | 0.723 |
| P06801 | NADP-dependent malic enzyme OS=Mus musculus GN=Me1 PE=1 SV=2 | 0.887 | 0.726 |
| P43275 | Histone H1.1 OS=Mus musculus GN=Hist1h1a PE=1 SV=2 | 0.887 | 0.727 |
| Q924X2 | Carnitine O-palmitoyltransferase 1, muscle isoform OS=Mus musculus GN=Cpt1b PE=1 SV=1 | 0.888 | 0.729 |
| Q9WVK4 | EH domain-containing protein 1 OS=Mus musculus GN=Ehd1 PE=1 SV=1 | 0.889 | 0.730 |
| Q91X72 | Hemopexin OS=Mus musculus GN=Hpx PE=1 SV=2 | 0.89 | 0.733 |
| Q543S0 | Prolargin OS=Mus musculus GN=Prelp PE=1 SV=1 | 0.891 | 0.734 |
| O35350 | Calpain-1 catalytic subunit OS=Mus musculus GN=Capn1 PE=1 SV=1 | 0.892 | 0.736 |
| H3BKH6 | S-formylglutathione hydrolase OS=Mus musculus GN=Esd PE=1 SV=1 | 0.892 | 0.737 |
| Q60692 | Proteasome subunit beta type-6 OS=Mus musculus GN=Psmb6 PE=1 SV=3 | 0.892 | 0.737 |
| O09167 | 60S ribosomal protein L21 OS=Mus musculus GN=Rpl21 PE=1 SV=3 | 0.892 | 0.737 |
| G3XA17 | Eukaryotic translation initiation factor 4 gamma 2 OS=Mus musculus GN=Eif4g2 PE=1 SV=1 | 0.893 | 0.739 |
| Q8BG32 | 26S proteasome non-ATPase regulatory subunit 11 OS=Mus musculus GN=Psmd11 PE=1 SV=3 | 0.893 | 0.740 |
| B2RTK3 | Histone H2B OS=Mus musculus GN=Hist1h2bm PE=1 SV=1 | 0.894 | 0.740 |
| P62264 | 40S ribosomal protein S14 OS=Mus musculus GN=Rps14 PE=1 SV=3 | 0.895 | 0.743 |
| Q9R1P0 | Proteasome subunit alpha type-4 OS=Mus musculus GN=Psma4 PE=1 SV=1 | 0.897 | 0.746 |
| Q9D0I9 | Arginine--tRNA ligase, cytoplasmic OS=Mus musculus GN=Rars PE=1 SV=2 | 0.898 | 0.748 |
| Q5M9M5 | 60S ribosomal protein L23a OS=Mus musculus GN=Rpl23a PE=1 SV=1 | 0.898 | 0.749 |
| Q91WU5 | Arsenite methyltransferase OS=Mus musculus GN=As3mt PE=1 SV=2 | 0.899 | 0.750 |
| P61222 | ATP-binding cassette sub-family E member 1 OS=Mus musculus GN=Abce1 PE=1 SV=1 | 0.9 | 0.752 |
| P35564 | Calnexin OS=Mus musculus GN=Canx PE=1 SV=1 | 0.901 | 0.755 |
| E9Q035 | Protein Gm20425 OS=Mus musculus GN=Gm20425 PE=4 SV=1 | 0.903 | 0.758 |
| P61164 | Alpha-centractin OS=Mus musculus GN=Actr1a PE=1 SV=1 | 0.904 | 0.760 |
| E9Q070 | 60S acidic ribosomal protein P0 OS=Mus musculus GN=Gm8730 PE=1 SV=1 | 0.905 | 0.763 |
| P61089 | Ubiquitin-conjugating enzyme E2 N OS=Mus musculus GN=Ube2n PE=1 SV=1 | 0.906 | 0.764 |
| Q3UKA4 | Alcohol dehydrogenase 1 OS=Mus musculus GN=Adh1 PE=1 SV=1 | 0.906 | 0.765 |
| P70670 | Nascent polypeptide-associated complex subunit alpha, muscle-specific form OS=Mus musculus GN=Naca PE=1 SV=2 | 0.907 | 0.766 |
| Q5DTJ9 | Myopalladin OS=Mus musculus GN=Mypn PE=1 SV=2 | 0.907 | 0.766 |
| Q61335 | B-cell receptor-associated protein 31 OS=Mus musculus GN=Bcap31 PE=1 SV=4 | 0.908 | 0.768 |
| Q3TEA8 | Heterochromatin protein 1-binding protein 3 OS=Mus musculus GN=Hp1bp3 PE=1 SV=1 | 0.909 | 0.771 |
| P05202 | Aspartate aminotransferase, mitochondrial OS=Mus musculus GN=Got2 PE=1 SV=1 | 0.91 | 0.771 |
| P50516 | V-type proton ATPase catalytic subunit A OS=Mus musculus GN=Atp6v1a PE=1 SV=2 | 0.912 | 0.776 |
| A6H6K1 | Aspn protein OS=Mus musculus GN=Aspn PE=1 SV=1 | 0.913 | 0.778 |
| Q58E64 | Elongation factor 1-alpha OS=Mus musculus GN=Eef1a1 PE=1 SV=1 | 0.914 | 0.780 |
| Q61425 | Hydroxyacyl-coenzyme A dehydrogenase, mitochondrial OS=Mus musculus GN=Hadh PE=1 SV=2 | 0.915 | 0.783 |
| Q3THE2 | Myosin regulatory light chain 12B OS=Mus musculus GN=Myl12b PE=1 SV=2 | 0.916 | 0.784 |
| Q3TG75 | Ornithine aminotransferase, isoform CRA_b OS=Mus musculus GN=Oat PE=1 SV=1 | 0.918 | 0.787 |
| Q9CQN1 | Heat shock protein 75 kDa, mitochondrial OS=Mus musculus GN=Trap1 PE=1 SV=1 | 0.918 | 0.788 |
| A0A0R4J1B0 | Troponin T, fast skeletal muscle OS=Mus musculus GN=Tnnt3 PE=1 SV=1 | 0.918 | 0.788 |
| E9QNP0 | KxDL motif-containing protein 1 OS=Mus musculus GN=Kxd1 PE=1 SV=1 | 0.919 | 0.790 |
| Q8QZT1 | Acetyl-CoA acetyltransferase, mitochondrial OS=Mus musculus GN=Acat1 PE=1 SV=1 | 0.92 | 0.792 |
| Q9WVA4 | Transgelin-2 OS=Mus musculus GN=Tagln2 PE=1 SV=4 | 0.924 | 0.799 |
| P02301 | Histone H3.3C OS=Mus musculus GN=H3f3c PE=3 SV=3 | 0.924 | 0.799 |
| Q9WUZ7 | SH3 domain-binding glutamic acid-rich protein OS=Mus musculus GN=Sh3bgr PE=1 SV=1 | 0.928 | 0.807 |
| Q8BML9 | Glutaminyl-tRNA synthetase OS=Mus musculus GN=Qars PE=1 SV=1 | 0.928 | 0.808 |
| Q99LC5 | Electron transfer flavoprotein subunit alpha, mitochondrial OS=Mus musculus GN=Etfa PE=1 SV=2 | 0.928 | 0.808 |
| P56480 | ATP synthase subunit beta, mitochondrial OS=Mus musculus GN=Atp5b PE=1 SV=2 | 0.929 | 0.810 |
| P26443 | Glutamate dehydrogenase 1, mitochondrial OS=Mus musculus GN=Glud1 PE=1 SV=1 | 0.931 | 0.814 |
| Q564D0 | Fusion, derived from t(12;16) malignant liposarcoma (Human), isoform CRA_a OS=Mus musculus GN=Fus PE=1 SV=1 | 0.932 | 0.815 |
| P37040 | NADPH--cytochrome P450 reductase OS=Mus musculus GN=Por PE=1 SV=2 | 0.932 | 0.816 |
| Q6P8J7 | Creatine kinase S-type, mitochondrial OS=Mus musculus GN=Ckmt2 PE=1 SV=1 | 0.934 | 0.819 |
| Q5SZA3 | Histone H1.2 OS=Mus musculus GN=Hist1h1c PE=1 SV=1 | 0.935 | 0.821 |
| Q4FJL0 | RAB10, member RAS oncogene family OS=Mus musculus GN=Rab10 PE=1 SV=1 | 0.935 | 0.821 |
| Q9CPU0 | Lactoylglutathione lyase OS=Mus musculus GN=Glo1 PE=1 SV=3 | 0.935 | 0.822 |
| Q91YQ5 | Dolichyl-diphosphooligosaccharide--protein glycosyltransferase subunit 1 OS=Mus musculus GN=Rpn1 PE=1 SV=1 | 0.935 | 0.822 |
| Q9DCJ5 | NADH dehydrogenase [ubiquinone] 1 alpha subcomplex subunit 8 OS=Mus musculus GN=Ndufa8 PE=1 SV=3 | 0.936 | 0.823 |
| Q8BGQ7 | Alanine--tRNA ligase, cytoplasmic OS=Mus musculus GN=Aars PE=1 SV=1 | 0.936 | 0.824 |
| Q62165 | Dystroglycan OS=Mus musculus GN=Dag1 PE=1 SV=4 | 0.937 | 0.824 |
| P35700 | Peroxiredoxin-1 OS=Mus musculus GN=Prdx1 PE=1 SV=1 | 0.937 | 0.825 |
| F8VQJ3 | Laminin subunit gamma-1 OS=Mus musculus GN=Lamc1 PE=1 SV=1 | 0.938 | 0.828 |
| E9PZ16 | Basement membrane-specific heparan sulfate proteoglycan core protein OS=Mus musculus GN=Hspg2 PE=1 SV=1 | 0.939 | 0.829 |
| P26516 | 26S proteasome non-ATPase regulatory subunit 7 OS=Mus musculus GN=Psmd7 PE=1 SV=2 | 0.94 | 0.832 |
| Q99LF4 | tRNA-splicing ligase RtcB homolog OS=Mus musculus GN=Rtcb PE=1 SV=1 | 0.943 | 0.836 |
| P15532 | Nucleoside diphosphate kinase A OS=Mus musculus GN=Nme1 PE=1 SV=1 | 0.944 | 0.838 |
| F8WJ05 | Inter-alpha-trypsin inhibitor heavy chain H1 OS=Mus musculus GN=Itih1 PE=1 SV=1 | 0.945 | 0.840 |
| Q62234-2 | Isoform 2 of Myomesin-1 OS=Mus musculus GN=Myom1 | 0.945 | 0.841 |
| Q3TXS7 | 26S proteasome non-ATPase regulatory subunit 1 OS=Mus musculus GN=Psmd1 PE=1 SV=1 | 0.948 | 0.846 |
| Q9CZU6 | Citrate synthase, mitochondrial OS=Mus musculus GN=Cs PE=1 SV=1 | 0.949 | 0.849 |
| Q8BWY3 | Eukaryotic peptide chain release factor subunit 1 OS=Mus musculus GN=Etf1 PE=1 SV=4 | 0.949 | 0.849 |
| Q921M7 | Protein FAM49B OS=Mus musculus GN=Fam49b PE=1 SV=1 | 0.95 | 0.850 |
| Q9WUR2 | Enoyl-CoA delta isomerase 2, mitochondrial OS=Mus musculus GN=Eci2 PE=1 SV=2 | 0.95 | 0.850 |
| P35486 | Pyruvate dehydrogenase E1 component subunit alpha, somatic form, mitochondrial OS=Mus musculus GN=Pdha1 PE=1 SV=1 | 0.95 | 0.850 |
| Q91VA7 | Isocitrate dehydrogenase [NAD] subunit, mitochondrial OS=Mus musculus GN=Idh3b PE=1 SV=1 | 0.95 | 0.851 |
| P51150 | Ras-related protein Rab-7a OS=Mus musculus GN=Rab7a PE=1 SV=2 | 0.951 | 0.853 |
| Q64727 | Vinculin OS=Mus musculus GN=Vcl PE=1 SV=4 | 0.953 | 0.856 |
| Q8CI43 | Myosin light chain 6B OS=Mus musculus GN=Myl6b PE=1 SV=1 | 0.954 | 0.857 |
| G5E8N5 | L-lactate dehydrogenase OS=Mus musculus GN=Ldha PE=1 SV=1 | 0.956 | 0.861 |
| P60843 | Eukaryotic initiation factor 4A-I OS=Mus musculus GN=Eif4a1 PE=1 SV=1 | 0.956 | 0.862 |
| P04117 | Fatty acid-binding protein, adipocyte OS=Mus musculus GN=Fabp4 PE=1 SV=3 | 0.956 | 0.862 |
| P23927 | Alpha-crystallin B chain OS=Mus musculus GN=Cryab PE=1 SV=2 | 0.957 | 0.864 |
| P28481 | Collagen alpha-1(II) chain OS=Mus musculus GN=Col2a1 PE=1 SV=2 | 0.96 | 0.870 |
| E9QJU4 | Myozenin-3 OS=Mus musculus GN=Myoz3 PE=1 SV=1 | 0.96 | 0.871 |
| P47857-3 | Isoform 3 of ATP-dependent 6-phosphofructokinase, muscle type OS=Mus musculus GN=Pfkm | 0.961 | 0.871 |
| P22599 | Alpha-1-antitrypsin 1-2 OS=Mus musculus GN=Serpina1b PE=1 SV=2 | 0.963 | 0.876 |
| D3Z041 | Long-chain-fatty-acid--CoA ligase 1 OS=Mus musculus GN=Acsl1 PE=1 SV=1 | 0.966 | 0.881 |
| Q9QZZ6 | Dermatopontin OS=Mus musculus GN=Dpt PE=1 SV=1 | 0.968 | 0.884 |
| P11404 | Fatty acid-binding protein, heart OS=Mus musculus GN=Fabp3 PE=1 SV=5 | 0.968 | 0.884 |
| O88322 | Nidogen-2 OS=Mus musculus GN=Nid2 PE=1 SV=2 | 0.969 | 0.887 |
| Q6ZWX6 | Eukaryotic translation initiation factor 2 subunit 1 OS=Mus musculus GN=Eif2s1 PE=1 SV=3 | 0.97 | 0.889 |
| F6RQN3 | Sarcoplasmic/endoplasmic reticulum calcium ATPase 1 (Fragment) OS=Mus musculus GN=Atp2a1 PE=1 SV=1 | 0.971 | 0.891 |
| Q9DBJ1 | Phosphoglycerate mutase 1 OS=Mus musculus GN=Pgam1 PE=1 SV=3 | 0.971 | 0.891 |
| Q56A15 | Cytochrome c OS=Mus musculus GN=Cycs PE=1 SV=1 | 0.972 | 0.893 |
| Q4VAG4 | 60S ribosomal protein L22 OS=Mus musculus GN=Rpl22 PE=1 SV=1 | 0.973 | 0.894 |
| Q923T9 | Calcium/calmodulin-dependent protein kinase type II subunit gamma OS=Mus musculus GN=Camk2g PE=1 SV=1 | 0.974 | 0.896 |
| G3X981 | Peripherin OS=Mus musculus GN=Prph PE=1 SV=1 | 0.975 | 0.899 |
| O35459 | Delta(3,5)-Delta(2,4)-dienoyl-CoA isomerase, mitochondrial OS=Mus musculus GN=Ech1 PE=1 SV=1 | 0.976 | 0.900 |
| P09405 | Nucleolin OS=Mus musculus GN=Ncl PE=1 SV=2 | 0.977 | 0.902 |
| O88990 | Alpha-actinin-3 OS=Mus musculus GN=Actn3 PE=2 SV=1 | 0.979 | 0.906 |
| Q3UBU9 | Peptidyl-prolyl cis-trans isomerase OS=Mus musculus GN=Fkbp3 PE=1 SV=1 | 0.983 | 0.913 |
| B7ZCF1 | 26S protease regulatory subunit 6A OS=Mus musculus GN=Psmc3 PE=1 SV=1 | 0.983 | 0.914 |
| Q99LY9 | NADH dehydrogenase [ubiquinone] iron-sulfur protein 5 OS=Mus musculus GN=Ndufs5 PE=1 SV=3 | 0.983 | 0.914 |
| P28650 | Adenylosuccinate synthetase isozyme 1 OS=Mus musculus GN=Adssl1 PE=1 SV=2 | 0.983 | 0.914 |
| Q9CZS1 | Aldehyde dehydrogenase X, mitochondrial OS=Mus musculus GN=Aldh1b1 PE=1 SV=1 | 0.984 | 0.915 |
| Q6R891 | Neurabin-2 OS=Mus musculus GN=Ppp1r9b PE=1 SV=1 | 0.984 | 0.915 |
| P68369 | Tubulin alpha-1A chain OS=Mus musculus GN=Tuba1a PE=1 SV=1 | 0.985 | 0.918 |
| Q9WU78-3 | Isoform 3 of Programmed cell death 6-interacting protein OS=Mus musculus GN=Pdcd6ip | 0.985 | 0.918 |
| Q8K1M6 | Dynamin-1-like protein OS=Mus musculus GN=Dnm1l PE=1 SV=2 | 0.989 | 0.926 |
| Q921G7 | Electron transfer flavoprotein-ubiquinone oxidoreductase, mitochondrial OS=Mus musculus GN=Etfdh PE=1 SV=1 | 0.99 | 0.926 |
| Q11011 | Puromycin-sensitive aminopeptidase OS=Mus musculus GN=Npepps PE=1 SV=2 | 0.99 | 0.928 |
| P63028 | Translationally-controlled tumor protein OS=Mus musculus GN=Tpt1 PE=1 SV=1 | 0.992 | 0.931 |
| Q9D0M3 | Cytochrome c1, heme protein, mitochondrial OS=Mus musculus GN=Cyc1 PE=1 SV=1 | 0.993 | 0.932 |
| P70404 | Isocitrate dehydrogenase [NAD] subunit gamma 1, mitochondrial OS=Mus musculus GN=Idh3g PE=1 SV=1 | 0.993 | 0.932 |
| A6ZI47 | Fructose-bisphosphate aldolase OS=Mus musculus GN=Aldoart2 PE=1 SV=1 | 0.994 | 0.934 |
| Q0PD65 | RAB2, member RAS oncogene family OS=Mus musculus GN=Rab2a PE=1 SV=1 | 0.994 | 0.935 |
| Q3UHK5 | Sodium/potassium-transporting ATPase subunit alpha OS=Mus musculus GN=Atp1a2 PE=1 SV=1 | 0.996 | 0.938 |
| Q99PR8 | Heat shock protein beta-2 OS=Mus musculus GN=Hspb2 PE=1 SV=2 | 0.997 | 0.941 |
| Q8R4N0 | Citrate lyase subunit beta-like protein, mitochondrial OS=Mus musculus GN=Clybl PE=1 SV=2 | 0.998 | 0.941 |
| Q9R112 | Sulfide:quinone oxidoreductase, mitochondrial OS=Mus musculus GN=Sqrdl PE=1 SV=3 | 0.998 | 0.941 |
| O35206 | Collagen alpha-1(XV) chain OS=Mus musculus GN=Col15a1 PE=1 SV=2 | 0.998 | 0.942 |
| Q8R1S0 | Ubiquinone biosynthesis monooxygenase COQ6, mitochondrial OS=Mus musculus GN=Coq6 PE=1 SV=3 | 1 | 0.946 |
| Q4VA32 | Acyl-coenzyme A thioesterase 13 OS=Mus musculus GN=Acot13 PE=1 SV=1 | 1 | 0.946 |
| Q7TMF3 | NADH dehydrogenase [ubiquinone] 1 alpha subcomplex subunit 12 OS=Mus musculus GN=Ndufa12 PE=1 SV=2 | 1 | 0.946 |
| A0A0R4J0X5 | Alpha-1-antitrypsin 1-3 OS=Mus musculus GN=Serpina1c PE=1 SV=1 | 1.002 | 0.950 |
| Q5FWB7 | Fructose-bisphosphate aldolase OS=Mus musculus GN=Aldoa PE=1 SV=1 | 1.003 | 0.951 |
| O70435 | Proteasome subunit alpha type-3 OS=Mus musculus GN=Psma3 PE=1 SV=3 | 1.003 | 0.952 |
| Q62261 | Spectrin beta chain, non-erythrocytic 1 OS=Mus musculus GN=Sptbn1 PE=1 SV=2 | 1.004 | 0.954 |
| F8WIT2 | Annexin OS=Mus musculus GN=Anxa6 PE=1 SV=1 | 1.005 | 0.954 |
| P19157 | Glutathione S-transferase P 1 OS=Mus musculus GN=Gstp1 PE=1 SV=2 | 1.005 | 0.956 |
| Q9D031 | Ras suppressor protein 1 OS=Mus musculus GN=Rsu1 PE=1 SV=1 | 1.008 | 0.961 |
| P17751 | Triosephosphate isomerase OS=Mus musculus GN=Tpi1 PE=1 SV=4 | 1.01 | 0.965 |
| B2RSC8 | E3 ubiquitin-protein ligase NEDD4 OS=Mus musculus GN=Nedd4 PE=1 SV=1 | 1.011 | 0.965 |
| Q04447 | Creatine kinase B-type OS=Mus musculus GN=Ckb PE=1 SV=1 | 1.011 | 0.966 |
| Q3TF14 | Adenosylhomocysteinase OS=Mus musculus GN=Ahcy PE=1 SV=1 | 1.013 | 0.970 |
| P51881 | ADP/ATP translocase 2 OS=Mus musculus GN=Slc25a5 PE=1 SV=3 | 1.013 | 0.970 |
| Q61584 | Fragile X mental retardation syndrome-related protein 1 OS=Mus musculus GN=Fxr1 PE=1 SV=2 | 1.014 | 0.971 |
| Q7TSQ8 | Pyruvate dehydrogenase phosphatase regulatory subunit, mitochondrial OS=Mus musculus GN=Pdpr PE=1 SV=1 | 1.015 | 0.974 |
| Q64521 | Glycerol-3-phosphate dehydrogenase, mitochondrial OS=Mus musculus GN=Gpd2 PE=1 SV=2 | 1.019 | 0.981 |
| P01027 | Complement C3 OS=Mus musculus GN=C3 PE=1 SV=3 | 1.02 | 0.982 |
| Q9JMA1 | Ubiquitin carboxyl-terminal hydrolase 14 OS=Mus musculus GN=Usp14 PE=1 SV=3 | 1.02 | 0.983 |
| Q91VK2 | Eef1d protein OS=Mus musculus GN=Eef1d PE=1 SV=1 | 1.021 | 0.984 |
| P63005 | Platelet-activating factor acetylhydrolase IB subunit alpha OS=Mus musculus GN=Pafah1b1 PE=1 SV=2 | 1.023 | 0.988 |
| Q5I0T8 | Ribosomal protein L19 OS=Mus musculus GN=Rpl19 PE=1 SV=1 | 1.023 | 0.989 |
| Q60737 | Casein kinase II subunit alpha OS=Mus musculus GN=Csnk2a1 PE=1 SV=2 | 1.024 | 0.990 |
| P54071 | Isocitrate dehydrogenase [NADP], mitochondrial OS=Mus musculus GN=Idh2 PE=1 SV=3 | 1.025 | 0.991 |
| Q4FZE6 | 40S ribosomal protein S7 OS=Mus musculus GN=Rps7 PE=1 SV=1 | 1.025 | 0.992 |
| Q8R1Q3 | Angiopoietin-related protein 7 OS=Mus musculus GN=Angptl7 PE=2 SV=1 | 1.026 | 0.993 |
| P08121 | Collagen alpha-1(III) chain OS=Mus musculus GN=Col3a1 PE=1 SV=4 | 1.027 | 0.995 |
| Q8VCR8 | Myosin light chain kinase 2, skeletal/cardiac muscle OS=Mus musculus GN=Mylk2 PE=1 SV=2 | 1.027 | 0.996 |
| Q9DB20 | ATP synthase subunit O, mitochondrial OS=Mus musculus GN=Atp5o PE=1 SV=1 | 1.028 | 0.998 |
| P47757 | F-actin-capping protein subunit beta OS=Mus musculus GN=Capzb PE=1 SV=3 | 1.03 | 0.999 |
| Q9ET78 | Junctophilin-2 OS=Mus musculus GN=Jph2 PE=1 SV=2 | 1.03 | 1.000 |
| P62737 | Actin, aortic smooth muscle OS=Mus musculus GN=Acta2 PE=1 SV=1 | 1.031 | 0.997 |
| Q9D1D4 | Transmembrane emp24 domain-containing protein 10 OS=Mus musculus GN=Tmed10 PE=1 SV=1 | 1.032 | 0.995 |
| Q9D358 | Low molecular weight phosphotyrosine protein phosphatase OS=Mus musculus GN=Acp1 PE=1 SV=3 | 1.032 | 0.996 |
| F8WIS9 | Calcium/calmodulin-dependent protein kinase type II subunit alpha OS=Mus musculus GN=Camk2a PE=1 SV=1 | 1.033 | 0.993 |
| Q8C494 | Proline-rich protein 33 OS=Mus musculus GN=Prr33 PE=2 SV=1 | 1.034 | 0.992 |
| Q8CGC7 | Bifunctional glutamate/proline--tRNA ligase OS=Mus musculus GN=Eprs PE=1 SV=4 | 1.036 | 0.988 |
| Q922B1 | O-acetyl-ADP-ribose deacetylase MACROD1 OS=Mus musculus GN=Macrod1 PE=1 SV=2 | 1.036 | 0.988 |
| Q8K3J1 | NADH dehydrogenase [ubiquinone] iron-sulfur protein 8, mitochondrial OS=Mus musculus GN=Ndufs8 PE=1 SV=1 | 1.036 | 0.988 |
| O55226 | Chondroadherin OS=Mus musculus GN=Chad PE=2 SV=1 | 1.036 | 0.989 |
| P26043 | Radixin OS=Mus musculus GN=Rdx PE=1 SV=3 | 1.037 | 0.987 |
| Q5EBQ6 | 60S ribosomal protein L9 OS=Mus musculus GN=Rpl9 PE=1 SV=1 | 1.038 | 0.984 |
| Q791V5 | Mitochondrial carrier homolog 2 OS=Mus musculus GN=Mtch2 PE=1 SV=1 | 1.038 | 0.985 |
| Q3UGX2 | Spectrin beta 1 OS=Mus musculus GN=Sptb PE=1 SV=1 | 1.039 | 0.983 |
| Q6R0H7 | Guanine nucleotide-binding protein G(s) subunit alpha isoforms XLas OS=Mus musculus GN=Gnas PE=1 SV=1 | 1.041 | 0.979 |
| Q59IW6 | Protein Abi3bp OS=Mus musculus GN=Abi3bp PE=1 SV=1 | 1.041 | 0.979 |
| Q8VBT1 | Beta-taxilin OS=Mus musculus GN=Txlnb PE=1 SV=2 | 1.042 | 0.978 |
| O08529 | Calpain-2 catalytic subunit OS=Mus musculus GN=Capn2 PE=1 SV=4 | 1.043 | 0.976 |
| P11499 | Heat shock protein HSP 90-beta OS=Mus musculus GN=Hsp90ab1 PE=1 SV=3 | 1.044 | 0.973 |
| P20152 | Vimentin OS=Mus musculus GN=Vim PE=1 SV=3 | 1.044 | 0.973 |
| Q9DCN2 | NADH-cytochrome b5 reductase 3 OS=Mus musculus GN=Cyb5r3 PE=1 SV=3 | 1.046 | 0.970 |
| A0A0A0MQA5 | Tubulin alpha-4A chain (Fragment) OS=Mus musculus GN=Tuba4a PE=1 SV=1 | 1.046 | 0.971 |
| P11352 | Glutathione peroxidase 1 OS=Mus musculus GN=Gpx1 PE=1 SV=2 | 1.047 | 0.968 |
| Q9JJV2 | Profilin-2 OS=Mus musculus GN=Pfn2 PE=1 SV=3 | 1.049 | 0.964 |
| P68134 | Actin, alpha skeletal muscle OS=Mus musculus GN=Acta1 PE=1 SV=1 | 1.049 | 0.965 |
| Q5DQJ3 | Capping protein (Actin filament) muscle Z-line, alpha 2, isoform CRA_c OS=Mus musculus GN=Capza2 PE=1 SV=1 | 1.053 | 0.958 |
| Q02257 | Junction plakoglobin OS=Mus musculus GN=Jup PE=1 SV=3 | 1.055 | 0.954 |
| P61971 | Nuclear transport factor 2 OS=Mus musculus GN=Nutf2 PE=1 SV=1 | 1.055 | 0.955 |
| Q9CQB4 | Cytochrome b-c1 complex subunit 7 OS=Mus musculus GN=Uqcrb PE=1 SV=1 | 1.056 | 0.952 |
| P62717 | 60S ribosomal protein L18a OS=Mus musculus GN=Rpl18a PE=1 SV=1 | 1.057 | 0.951 |
| P80317 | T-complex protein 1 subunit zeta OS=Mus musculus GN=Cct6a PE=1 SV=3 | 1.057 | 0.951 |
| O55023 | Inositol monophosphatase 1 OS=Mus musculus GN=Impa1 PE=1 SV=1 | 1.058 | 0.949 |
| Q20BD0 | Heterogeneous nuclear ribonucleoprotein A/B OS=Mus musculus GN=Hnrnpab PE=1 SV=1 | 1.06 | 0.946 |
| Q3UGC8 | Propionyl-CoA carboxylase alpha chain, mitochondrial OS=Mus musculus GN=Pcca PE=1 SV=1 | 1.06 | 0.946 |
| Q9JMC3 | DnaJ homolog subfamily A member 4 OS=Mus musculus GN=Dnaja4 PE=1 SV=1 | 1.061 | 0.943 |
| P14115 | 60S ribosomal protein L27a OS=Mus musculus GN=Rpl27a PE=1 SV=5 | 1.062 | 0.941 |
| Q6ZWZ7 | 60S ribosomal protein L17 OS=Mus musculus GN=Rpl17 PE=1 SV=1 | 1.063 | 0.940 |
| Q9DCL9 | Multifunctional protein ADE2 OS=Mus musculus GN=Paics PE=1 SV=4 | 1.063 | 0.940 |
| Q5EBQ2 | MCG7941, isoform CRA_f OS=Mus musculus GN=Pebp1 PE=1 SV=1 | 1.064 | 0.938 |
| A2AKU9 | ATP synthase subunit gamma OS=Mus musculus GN=Atp5c1 PE=1 SV=1 | 1.064 | 0.938 |
| P24527 | Leukotriene A-4 hydrolase OS=Mus musculus GN=Lta4h PE=1 SV=4 | 1.067 | 0.933 |
| A2RSV8 | Cytochrome c oxidase subunit 4 isoform 1, mitochondrial OS=Mus musculus GN=Cox4i1 PE=1 SV=1 | 1.067 | 0.933 |
| P50544 | Very long-chain specific acyl-CoA dehydrogenase, mitochondrial OS=Mus musculus GN=Acadvl PE=1 SV=3 | 1.067 | 0.933 |
| Q9WTI7 | Unconventional myosin-Ic OS=Mus musculus GN=Myo1c PE=1 SV=2 | 1.068 | 0.932 |
| Q9R0G6 | Cartilage oligomeric matrix protein OS=Mus musculus GN=Comp PE=1 SV=2 | 1.07 | 0.928 |
| Q9EQ20 | Methylmalonate-semialdehyde dehydrogenase [acylating], mitochondrial OS=Mus musculus GN=Aldh6a1 PE=1 SV=1 | 1.07 | 0.928 |
| Q60930 | Voltage-dependent anion-selective channel protein 2 OS=Mus musculus GN=Vdac2 PE=1 SV=2 | 1.071 | 0.926 |
| P49813 | Tropomodulin-1 OS=Mus musculus GN=Tmod1 PE=1 SV=2 | 1.072 | 0.925 |
| P11531 | Dystrophin OS=Mus musculus GN=Dmd PE=1 SV=3 | 1.073 | 0.922 |
| Q9CZ13 | Cytochrome b-c1 complex subunit 1, mitochondrial OS=Mus musculus GN=Uqcrc1 PE=1 SV=2 | 1.074 | 0.921 |
| Q9JKS4-4 | Isoform 4 of LIM domain-binding protein 3 OS=Mus musculus GN=Ldb3 | 1.075 | 0.919 |
| P61255 | 60S ribosomal protein L26 OS=Mus musculus GN=Rpl26 PE=1 SV=1 | 1.075 | 0.920 |
| P47962 | 60S ribosomal protein L5 OS=Mus musculus GN=Rpl5 PE=1 SV=3 | 1.076 | 0.918 |
| Q9Z2I9 | Succinyl-CoA ligase [ADP-forming] subunit beta, mitochondrial OS=Mus musculus GN=Sucla2 PE=1 SV=2 | 1.076 | 0.918 |
| Q561N4 | MCG1032217 OS=Mus musculus GN=Ube2l3 PE=1 SV=1 | 1.076 | 0.918 |
| O88342 | WD repeat-containing protein 1 OS=Mus musculus GN=Wdr1 PE=1 SV=3 | 1.076 | 0.918 |
| Q91VD9 | NADH-ubiquinone oxidoreductase 75 kDa subunit, mitochondrial OS=Mus musculus GN=Ndufs1 PE=1 SV=2 | 1.078 | 0.913 |
| Q58EV5 | High mobility group box 1 OS=Mus musculus GN=Hmgb1 PE=1 SV=1 | 1.078 | 0.914 |
| Q9D6J6 | NADH dehydrogenase [ubiquinone] flavoprotein 2, mitochondrial OS=Mus musculus GN=Ndufv2 PE=1 SV=2 | 1.078 | 0.915 |
| E9Q9K5 | Triadin OS=Mus musculus GN=Trdn PE=1 SV=1 | 1.08 | 0.910 |
| Q8C7E7 | Starch-binding domain-containing protein 1 OS=Mus musculus GN=Stbd1 PE=1 SV=1 | 1.081 | 0.908 |
| P82347 | Delta-sarcoglycan OS=Mus musculus GN=Sgcd PE=1 SV=1 | 1.082 | 0.907 |
| B1AQF4 | Dual-specificity protein phosphatase 3 OS=Mus musculus GN=Dusp3 PE=1 SV=1 | 1.082 | 0.907 |
| E9QQ57 | Periaxin OS=Mus musculus GN=Prx PE=1 SV=1 | 1.083 | 0.906 |
| A0A087WNT1 | Transcription elongation factor B polypeptide 1 OS=Mus musculus GN=Tceb1 PE=1 SV=1 | 1.083 | 0.906 |
| Q9DCX2 | ATP synthase subunit d, mitochondrial OS=Mus musculus GN=Atp5h PE=1 SV=3 | 1.084 | 0.904 |
| Q9DC70 | NADH dehydrogenase [ubiquinone] iron-sulfur protein 7, mitochondrial OS=Mus musculus GN=Ndufs7 PE=1 SV=1 | 1.084 | 0.904 |
| E9Q0S6 | Protein Tns1 OS=Mus musculus GN=Tns1 PE=1 SV=1 | 1.085 | 0.902 |
| Q9Z1E4 | Glycogen [starch] synthase, muscle OS=Mus musculus GN=Gys1 PE=1 SV=2 | 1.085 | 0.902 |
| E9PYB0 | Protein Ahnak2 (Fragment) OS=Mus musculus GN=Ahnak2 PE=1 SV=7 | 1.085 | 0.902 |
| A0A0U1RP93 | Myosin regulatory light chain 2, skeletal muscle isoform OS=Mus musculus GN=Mylpf PE=1 SV=1 | 1.086 | 0.900 |
| Q9D1G3 | Protein-cysteine N-palmitoyltransferase HHAT-like protein OS=Mus musculus GN=Hhatl PE=1 SV=2 | 1.088 | 0.898 |
| E9PYL9 | Protein Gm10036 OS=Mus musculus GN=Gm10036 PE=3 SV=1 | 1.09 | 0.893 |
| Q4FJK0 | 2,4-dienoyl-CoA reductase, mitochondrial OS=Mus musculus GN=Decr1 PE=1 SV=1 | 1.09 | 0.893 |
| O08553 | Dihydropyrimidinase-related protein 2 OS=Mus musculus GN=Dpysl2 PE=1 SV=2 | 1.09 | 0.893 |
| Q9ET80 | Junctophilin-1 OS=Mus musculus GN=Jph1 PE=1 SV=1 | 1.091 | 0.892 |
| Q9WUM4 | Coronin-1C OS=Mus musculus GN=Coro1c PE=1 SV=2 | 1.092 | 0.890 |
| Q6ZQ73 | Cullin-associated NEDD8-dissociated protein 2 OS=Mus musculus GN=Cand2 PE=1 SV=2 | 1.092 | 0.891 |
| O08528 | Hexokinase-2 OS=Mus musculus GN=Hk2 PE=1 SV=1 | 1.093 | 0.888 |
| P68372 | Tubulin beta-4B chain OS=Mus musculus GN=Tubb4b PE=1 SV=1 | 1.095 | 0.885 |
| Q6ZWN5 | 40S ribosomal protein S9 OS=Mus musculus GN=Rps9 PE=1 SV=3 | 1.098 | 0.880 |
| P62918 | 60S ribosomal protein L8 OS=Mus musculus GN=Rpl8 PE=1 SV=2 | 1.099 | 0.878 |
| Q5SS40 | 14-3-3 protein epsilon OS=Mus musculus GN=Ywhae PE=1 SV=1 | 1.099 | 0.878 |
| Q61738 | Integrin alpha-7 OS=Mus musculus GN=Itga7 PE=1 SV=3 | 1.101 | 0.874 |
| O09110 | Dual specificity mitogen-activated protein kinase kinase 3 OS=Mus musculus GN=Map2k3 PE=1 SV=2 | 1.101 | 0.875 |
| Q9JHI5 | Isovaleryl-CoA dehydrogenase, mitochondrial OS=Mus musculus GN=Ivd PE=1 SV=1 | 1.101 | 0.875 |
| P53395 | Lipoamide acyltransferase component of branched-chain alpha-keto acid dehydrogenase complex, mitochondrial OS=Mus musculus GN=Dbt PE=1 SV=2 | 1.103 | 0.872 |
| Q9Z0N1 | Eukaryotic translation initiation factor 2 subunit 3, X-linked OS=Mus musculus GN=Eif2s3x PE=1 SV=2 | 1.104 | 0.870 |
| Q9QZD9 | Eukaryotic translation initiation factor 3 subunit I OS=Mus musculus GN=Eif3i PE=1 SV=1 | 1.105 | 0.868 |
| Q3V235 | Prohibitin 2 OS=Mus musculus GN=Phb2 PE=1 SV=1 | 1.106 | 0.867 |
| P63323 | 40S ribosomal protein S12 OS=Mus musculus GN=Rps12 PE=1 SV=2 | 1.108 | 0.863 |
| Q5M9K7 | 40S ribosomal protein S10 OS=Mus musculus GN=Rps10 PE=1 SV=1 | 1.108 | 0.864 |
| Q9R0P9 | Ubiquitin carboxyl-terminal hydrolase isozyme L1 OS=Mus musculus GN=Uchl1 PE=1 SV=1 | 1.109 | 0.861 |
| O55222 | Integrin-linked protein kinase OS=Mus musculus GN=Ilk PE=1 SV=2 | 1.109 | 0.862 |
| Q8CHS7 | Dehydrogenase/reductase SDR family member 7C OS=Mus musculus GN=Dhrs7c PE=1 SV=3 | 1.109 | 0.862 |
| Q8C0C7 | Phenylalanine--tRNA ligase alpha subunit OS=Mus musculus GN=Farsa PE=1 SV=1 | 1.11 | 0.860 |
| P18572 | Basigin OS=Mus musculus GN=Bsg PE=1 SV=2 | 1.111 | 0.858 |
| E9QPX3 | NADH dehydrogenase [ubiquinone] iron-sulfur protein 4, mitochondrial OS=Mus musculus GN=Ndufs4 PE=1 SV=1 | 1.112 | 0.856 |
| Q91WC3-2 | Isoform 2 of Long-chain-fatty-acid--CoA ligase 6 OS=Mus musculus GN=Acsl6 | 1.115 | 0.852 |
| P48036 | Annexin A5 OS=Mus musculus GN=Anxa5 PE=1 SV=1 | 1.118 | 0.846 |
| Q9D2N4 | Dystrobrevin alpha OS=Mus musculus GN=Dtna PE=1 SV=2 | 1.118 | 0.846 |
| Q93092 | Transaldolase OS=Mus musculus GN=Taldo1 PE=1 SV=2 | 1.118 | 0.847 |
| A0A0R4J0I1 | MCG1051009 OS=Mus musculus GN=Serpina3k PE=1 SV=1 | 1.119 | 0.845 |
| A0A0R4J083 | Long-chain-specific acyl-CoA dehydrogenase, mitochondrial OS=Mus musculus GN=Acadl PE=1 SV=1 | 1.121 | 0.842 |
| Q6PDI5 | Proteasome-associated protein ECM29 homolog OS=Mus musculus GN=Ecm29 PE=1 SV=3 | 1.122 | 0.840 |
| P08228 | Superoxide dismutase [Cu-Zn] OS=Mus musculus GN=Sod1 PE=1 SV=2 | 1.122 | 0.841 |
| Q03265 | ATP synthase subunit alpha, mitochondrial OS=Mus musculus GN=Atp5a1 PE=1 SV=1 | 1.122 | 0.841 |
| P62827 | GTP-binding nuclear protein Ran OS=Mus musculus GN=Ran PE=1 SV=3 | 1.122 | 0.841 |
| Q8BZF8 | Phosphoglucomutase-like protein 5 OS=Mus musculus GN=Pgm5 PE=1 SV=2 | 1.122 | 0.841 |
| Q9CXS4 | Centromere protein V OS=Mus musculus GN=Cenpv PE=1 SV=2 | 1.123 | 0.838 |
| P17879 | Heat shock 70 kDa protein 1B OS=Mus musculus GN=Hspa1b PE=1 SV=3 | 1.123 | 0.839 |
| P09055-2 | Isoform 2 of Integrin beta-1 OS=Mus musculus GN=Itgb1 | 1.123 | 0.839 |
| A2A6J8 | Troponin I, fast skeletal muscle (Fragment) OS=Mus musculus GN=Tnni2 PE=1 SV=1 | 1.125 | 0.835 |
| Q9EQ83 | Gamma sarcoglycan OS=Mus musculus GN=Sgcg PE=1 SV=1 | 1.126 | 0.834 |
| P46412 | Glutathione peroxidase 3 OS=Mus musculus GN=Gpx3 PE=1 SV=2 | 1.128 | 0.831 |
| Q7TSH2 | Phosphorylase b kinase regulatory subunit beta OS=Mus musculus GN=Phkb PE=1 SV=1 | 1.128 | 0.831 |
| Q8R2P8 | Lysine--tRNA ligase OS=Mus musculus GN=Kars PE=1 SV=1 | 1.129 | 0.828 |
| P99026 | Proteasome subunit beta type-4 OS=Mus musculus GN=Psmb4 PE=1 SV=1 | 1.13 | 0.828 |
| P05977 | Myosin light chain 1/3, skeletal muscle isoform OS=Mus musculus GN=Myl1 PE=1 SV=2 | 1.131 | 0.825 |
| Q61171 | Peroxiredoxin-2 OS=Mus musculus GN=Prdx2 PE=1 SV=3 | 1.131 | 0.826 |
| Q8CG76 | Aflatoxin B1 aldehyde reductase member 2 OS=Mus musculus GN=Akr7a2 PE=1 SV=3 | 1.134 | 0.820 |
| E9Q616 | Protein Ahnak OS=Mus musculus GN=Ahnak PE=1 SV=1 | 1.135 | 0.818 |
| Q3V1D3 | AMP deaminase 1 OS=Mus musculus GN=Ampd1 PE=1 SV=2 | 1.135 | 0.819 |
| P62962 | Profilin-1 OS=Mus musculus GN=Pfn1 PE=1 SV=2 | 1.135 | 0.819 |
| Q9JK37 | Myozenin-1 OS=Mus musculus GN=Myoz1 PE=1 SV=1 | 1.136 | 0.817 |
| P58774 | Tropomyosin beta chain OS=Mus musculus GN=Tpm2 PE=1 SV=1 | 1.136 | 0.818 |
| P05132 | cAMP-dependent protein kinase catalytic subunit alpha OS=Mus musculus GN=Prkaca PE=1 SV=3 | 1.136 | 0.818 |
| A2AQB2 | Protein Neb OS=Mus musculus GN=Neb PE=1 SV=1 | 1.137 | 0.816 |
| A2AEX8 | Four and a half LIM domains 1, isoform CRA_b OS=Mus musculus GN=Fhl1 PE=1 SV=1 | 1.138 | 0.814 |
| Q5EBP8 | Heterogeneous nuclear ribonucleoprotein A1 OS=Mus musculus GN=Hnrnpa1 PE=1 SV=1 | 1.138 | 0.815 |
| P40142 | Transketolase OS=Mus musculus GN=Tkt PE=1 SV=1 | 1.141 | 0.809 |
| P14211 | Calreticulin OS=Mus musculus GN=Calr PE=1 SV=1 | 1.141 | 0.810 |
| P18242 | Cathepsin D OS=Mus musculus GN=Ctsd PE=1 SV=1 | 1.143 | 0.807 |
| Q8R010 | Aminoacyl tRNA synthase complex-interacting multifunctional protein 2 OS=Mus musculus GN=Aimp2 PE=1 SV=2 | 1.144 | 0.805 |
| Q8BG13 | RNA-binding protein 3 OS=Mus musculus GN=Rbm3 PE=1 SV=1 | 1.144 | 0.805 |
| Q63918 | Serum deprivation-response protein OS=Mus musculus GN=Sdpr PE=1 SV=3 | 1.145 | 0.804 |
| A2AJQ0 | [Pyruvate dehydrogenase [acetyl-transferring]]-phosphatase 1, mitochondrial OS=Mus musculus GN=Pdp1 PE=1 SV=2 | 1.146 | 0.802 |
| E0CYV0 | Protein-L-isoaspartate O-methyltransferase OS=Mus musculus GN=Pcmt1 PE=1 SV=1 | 1.149 | 0.796 |
| Q9D0F9 | Phosphoglucomutase-1 OS=Mus musculus GN=Pgm1 PE=1 SV=4 | 1.149 | 0.797 |
| P97351 | 40S ribosomal protein S3a OS=Mus musculus GN=Rps3a PE=1 SV=3 | 1.151 | 0.794 |
| Q9WUB3 | Glycogen phosphorylase, muscle form OS=Mus musculus GN=Pygm PE=1 SV=3 | 1.151 | 0.794 |
| P26041 | Moesin OS=Mus musculus GN=Msn PE=1 SV=3 | 1.152 | 0.792 |
| D3YVN7 | Elongation factor Tu OS=Mus musculus GN=Gm9755 PE=3 SV=1 | 1.153 | 0.790 |
| O70209 | PDZ and LIM domain protein 3 OS=Mus musculus GN=Pdlim3 PE=1 SV=1 | 1.154 | 0.788 |
| Q6XLQ8 | Calumenin OS=Mus musculus GN=Calu PE=1 SV=1 | 1.154 | 0.789 |
| Q9JJZ2 | Tubulin alpha-8 chain OS=Mus musculus GN=Tuba8 PE=1 SV=1 | 1.154 | 0.789 |
| P08551 | Neurofilament light polypeptide OS=Mus musculus GN=Nefl PE=1 SV=5 | 1.154 | 0.790 |
| P45952 | Medium-chain specific acyl-CoA dehydrogenase, mitochondrial OS=Mus musculus GN=Acadm PE=1 SV=1 | 1.159 | 0.781 |
| Q8K2B3 | Succinate dehydrogenase [ubiquinone] flavoprotein subunit, mitochondrial OS=Mus musculus GN=Sdha PE=1 SV=1 | 1.159 | 0.782 |
| Q3TM70 | EH domain-containing protein 4 OS=Mus musculus GN=Ehd4 PE=1 SV=1 | 1.16 | 0.779 |
| Z4YNB2 | Troponin T, fast skeletal muscle OS=Mus musculus GN=Tnnt3 PE=1 SV=1 | 1.16 | 0.780 |
| Q9JHU4 | Cytoplasmic dynein 1 heavy chain 1 OS=Mus musculus GN=Dync1h1 PE=1 SV=2 | 1.161 | 0.778 |
| Q5FW97 | Alpha-enolase OS=Mus musculus GN=EG433182 PE=1 SV=1 | 1.161 | 0.778 |
| P50462 | Cysteine and glycine-rich protein 3 OS=Mus musculus GN=Csrp3 PE=1 SV=1 | 1.162 | 0.776 |
| Q8CHT0 | Delta-1-pyrroline-5-carboxylate dehydrogenase, mitochondrial OS=Mus musculus GN=Aldh4a1 PE=1 SV=3 | 1.162 | 0.777 |
| Q9CWF2 | Tubulin beta-2B chain OS=Mus musculus GN=Tubb2b PE=1 SV=1 | 1.166 | 0.770 |
| Q9Z2X1 | Heterogeneous nuclear ribonucleoprotein F OS=Mus musculus GN=Hnrnpf PE=1 SV=3 | 1.166 | 0.770 |
| Q8R429 | Sarcoplasmic/endoplasmic reticulum calcium ATPase 1 OS=Mus musculus GN=Atp2a1 PE=1 SV=1 | 1.167 | 0.769 |
| Q3TX57 | Collagen alpha-2(I) chain OS=Mus musculus GN=Col1a2 PE=1 SV=1 | 1.168 | 0.767 |
| O70624 | Myocilin OS=Mus musculus GN=Myoc PE=1 SV=1 | 1.17 | 0.764 |
| Q4FJX9 | Superoxide dismutase OS=Mus musculus GN=Sod2 PE=1 SV=1 | 1.17 | 0.764 |
| Q9CPY7 | Cytosol aminopeptidase OS=Mus musculus GN=Lap3 PE=1 SV=3 | 1.171 | 0.763 |
| A8IP69 | 14-3-3 protein gamma OS=Mus musculus GN=Ywhag PE=1 SV=1 | 1.171 | 0.763 |
| Q80YX1 | Tenascin OS=Mus musculus GN=Tnc PE=1 SV=1 | 1.172 | 0.761 |
| A2ASS6 | Titin OS=Mus musculus GN=Ttn PE=1 SV=1 | 1.172 | 0.761 |
| Q3TGR2 | Fibrinogen beta chain OS=Mus musculus GN=Fgb PE=1 SV=1 | 1.173 | 0.759 |
| Q9CY58 | Plasminogen activator inhibitor 1 RNA-binding protein OS=Mus musculus GN=Serbp1 PE=1 SV=2 | 1.174 | 0.758 |
| Q9D051 | Pyruvate dehydrogenase E1 component subunit beta, mitochondrial OS=Mus musculus GN=Pdhb PE=1 SV=1 | 1.174 | 0.759 |
| Q9QXS1 | Plectin OS=Mus musculus GN=Plec PE=1 SV=3 | 1.175 | 0.756 |
| Q91YT0 | NADH dehydrogenase [ubiquinone] flavoprotein 1, mitochondrial OS=Mus musculus GN=Ndufv1 PE=1 SV=1 | 1.177 | 0.753 |
| P62141 | Serine/threonine-protein phosphatase PP1-beta catalytic subunit OS=Mus musculus GN=Ppp1cb PE=1 SV=3 | 1.177 | 0.754 |
| Q91XH5 | Sepiapterin reductase OS=Mus musculus GN=Spr PE=1 SV=1 | 1.178 | 0.752 |
| B2RQK7 | Synaptopodin 2-like protein OS=Mus musculus GN=Synpo2l PE=1 SV=1 | 1.18 | 0.750 |
| O88346 | Troponin T, slow skeletal muscle OS=Mus musculus GN=Tnnt1 PE=2 SV=3 | 1.181 | 0.747 |
| P47934 | Carnitine O-acetyltransferase OS=Mus musculus GN=Crat PE=1 SV=3 | 1.181 | 0.747 |
| P60824 | Cold-inducible RNA-binding protein OS=Mus musculus GN=Cirbp PE=1 SV=1 | 1.182 | 0.746 |
| Q99L47 | Hsc70-interacting protein OS=Mus musculus GN=St13 PE=1 SV=1 | 1.183 | 0.745 |
| Q5BLK2 | 40S ribosomal protein S20 OS=Mus musculus GN=Rps20 PE=1 SV=1 | 1.185 | 0.741 |
| Q61941 | NAD(P) transhydrogenase, mitochondrial OS=Mus musculus GN=Nnt PE=1 SV=2 | 1.188 | 0.737 |
| P21107 | Tropomyosin alpha-3 chain OS=Mus musculus GN=Tpm3 PE=1 SV=3 | 1.188 | 0.737 |
| P14152 | Malate dehydrogenase, cytoplasmic OS=Mus musculus GN=Mdh1 PE=1 SV=3 | 1.189 | 0.735 |
| Q5M9K9 | 60S ribosomal protein L31 OS=Mus musculus GN=Rpl31 PE=1 SV=1 | 1.189 | 0.736 |
| Q9D020 | Cytosolic 5'-nucleotidase 3A OS=Mus musculus GN=Nt5c3a PE=1 SV=4 | 1.191 | 0.732 |
| Q8BWT1 | 3-ketoacyl-CoA thiolase, mitochondrial OS=Mus musculus GN=Acaa2 PE=1 SV=3 | 1.193 | 0.729 |
| P28665 | Murinoglobulin-1 OS=Mus musculus GN=Mug1 PE=1 SV=3 | 1.194 | 0.728 |
| P47811 | Mitogen-activated protein kinase 14 OS=Mus musculus GN=Mapk14 PE=1 SV=3 | 1.196 | 0.726 |
| Q99JY0 | Trifunctional enzyme subunit beta, mitochondrial OS=Mus musculus GN=Hadhb PE=1 SV=1 | 1.197 | 0.724 |
| P08752 | Guanine nucleotide-binding protein G(i) subunit alpha-2 OS=Mus musculus GN=Gnai2 PE=1 SV=5 | 1.197 | 0.724 |
| P58771 | Tropomyosin alpha-1 chain OS=Mus musculus GN=Tpm1 PE=1 SV=1 | 1.197 | 0.724 |
| A0A0R4J166 | Kelch-like protein 40 OS=Mus musculus GN=Klhl40 PE=1 SV=1 | 1.198 | 0.722 |
| Q5SXR6 | Clathrin heavy chain OS=Mus musculus GN=Cltc PE=1 SV=1 | 1.199 | 0.720 |
| Q9DB77 | Cytochrome b-c1 complex subunit 2, mitochondrial OS=Mus musculus GN=Uqcrc2 PE=1 SV=1 | 1.2 | 0.719 |
| P10605 | Cathepsin B OS=Mus musculus GN=Ctsb PE=1 SV=2 | 1.206 | 0.711 |
| P48962 | ADP/ATP translocase 1 OS=Mus musculus GN=Slc25a4 PE=1 SV=4 | 1.207 | 0.710 |
| Q9CQZ6 | NADH dehydrogenase [ubiquinone] 1 beta subcomplex subunit 3 OS=Mus musculus GN=Ndufb3 PE=1 SV=1 | 1.207 | 0.710 |
| P27773 | Protein disulfide-isomerase A3 OS=Mus musculus GN=Pdia3 PE=1 SV=2 | 1.21 | 0.705 |
| D3YUE2 | Procollagen C-endopeptidase enhancer 1 OS=Mus musculus GN=Pcolce PE=1 SV=1 | 1.21 | 0.705 |
| Q9CPV4 | Glyoxalase domain-containing protein 4 OS=Mus musculus GN=Glod4 PE=1 SV=1 | 1.211 | 0.704 |
| Q8C2Q7 | Heterogeneous nuclear ribonucleoprotein H OS=Mus musculus GN=Hnrnph1 PE=1 SV=1 | 1.211 | 0.704 |
| Q9R0Y5-2 | Isoform 2 of Adenylate kinase isoenzyme 1 OS=Mus musculus GN=Ak1 | 1.211 | 0.704 |
| P97807 | Fumarate hydratase, mitochondrial OS=Mus musculus GN=Fh PE=1 SV=3 | 1.213 | 0.700 |
| Q9WUM5 | Succinyl-CoA ligase [ADP/GDP-forming] subunit alpha, mitochondrial OS=Mus musculus GN=Suclg1 PE=1 SV=4 | 1.213 | 0.700 |
| P62715 | Serine/threonine-protein phosphatase 2A catalytic subunit beta isoform OS=Mus musculus GN=Ppp2cb PE=1 SV=1 | 1.214 | 0.699 |
| P62267 | 40S ribosomal protein S23 OS=Mus musculus GN=Rps23 PE=1 SV=3 | 1.214 | 0.699 |
| F6RQD1 | Protein Mybpc1 (Fragment) OS=Mus musculus GN=Mybpc1 PE=1 SV=1 | 1.214 | 0.699 |
| O70423 | Membrane primary amine oxidase OS=Mus musculus GN=Aoc3 PE=1 SV=3 | 1.215 | 0.698 |
| A0A0R4J1N4 | NAD(P)(+)--arginine ADP-ribosyltransferase OS=Mus musculus GN=Art3 PE=1 SV=1 | 1.216 | 0.697 |
| Q99KI0 | Aconitate hydratase, mitochondrial OS=Mus musculus GN=Aco2 PE=1 SV=1 | 1.217 | 0.695 |
| P06745 | Glucose-6-phosphate isomerase OS=Mus musculus GN=Gpi PE=1 SV=4 | 1.22 | 0.691 |
| O88569 | Heterogeneous nuclear ribonucleoproteins A2/B1 OS=Mus musculus GN=Hnrnpa2b1 PE=1 SV=2 | 1.22 | 0.691 |
| V9GWW6 | Muscular LMNA-interacting protein OS=Mus musculus GN=Mlip PE=1 SV=1 | 1.22 | 0.691 |
| Q921H8 | 3-ketoacyl-CoA thiolase A, peroxisomal OS=Mus musculus GN=Acaa1a PE=1 SV=1 | 1.222 | 0.688 |
| Q9WVJ2 | 26S proteasome non-ATPase regulatory subunit 13 OS=Mus musculus GN=Psmd13 PE=1 SV=1 | 1.223 | 0.686 |
| P58252 | Elongation factor 2 OS=Mus musculus GN=Eef2 PE=1 SV=2 | 1.224 | 0.685 |
| Q9CRB9 | MICOS complex subunit Mic19 OS=Mus musculus GN=Chchd3 PE=1 SV=1 | 1.225 | 0.683 |
| Q9CRA2 | PDZ and LIM domain protein 5 OS=Mus musculus GN=Pdlim5 PE=1 SV=1 | 1.225 | 0.684 |
| Q14BI5 | Myomesin 2 OS=Mus musculus GN=Myom2 PE=1 SV=1 | 1.228 | 0.680 |
| P38647 | Stress-70 protein, mitochondrial OS=Mus musculus GN=Hspa9 PE=1 SV=3 | 1.229 | 0.678 |
| Q64433 | 10 kDa heat shock protein, mitochondrial OS=Mus musculus GN=Hspe1 PE=1 SV=2 | 1.229 | 0.678 |
| Q91YE8 | Synaptopodin-2 OS=Mus musculus GN=Synpo2 PE=1 SV=2 | 1.233 | 0.672 |
| Q3TJD7-2 | Isoform 2 of PDZ and LIM domain protein 7 OS=Mus musculus GN=Pdlim7 | 1.234 | 0.671 |
| P07309 | Transthyretin OS=Mus musculus GN=Ttr PE=1 SV=1 | 1.236 | 0.669 |
| P45591 | Cofilin-2 OS=Mus musculus GN=Cfl2 PE=1 SV=1 | 1.236 | 0.669 |
| P70402 | Myosin-binding protein H OS=Mus musculus GN=Mybph PE=2 SV=2 | 1.237 | 0.667 |
| P07310 | Creatine kinase M-type OS=Mus musculus GN=Ckm PE=1 SV=1 | 1.238 | 0.665 |
| Q8BVI4 | Dihydropteridine reductase OS=Mus musculus GN=Qdpr PE=1 SV=2 | 1.239 | 0.665 |
| Q8R1G2 | Carboxymethylenebutenolidase homolog OS=Mus musculus GN=Cmbl PE=1 SV=1 | 1.24 | 0.663 |
| P80315 | T-complex protein 1 subunit delta OS=Mus musculus GN=Cct4 PE=1 SV=3 | 1.24 | 0.663 |
| B2RTL6 | Thrombospondin 4 OS=Mus musculus GN=Thbs4 PE=1 SV=1 | 1.241 | 0.661 |
| F6ZHD8 | 1,4-alpha-glucan-branching enzyme OS=Mus musculus GN=Gbe1 PE=1 SV=2 | 1.242 | 0.661 |
| A0A509 | MCG4625 OS=Mus musculus GN=Myot PE=1 SV=1 | 1.246 | 0.654 |
| Q80Y52 | Heat shock protein 90, alpha (Cytosolic), class A member 1 OS=Mus musculus GN=Hsp90aa1 PE=1 SV=2 | 1.247 | 0.653 |
| F8VPN4 | Protein Agl OS=Mus musculus GN=Agl PE=1 SV=1 | 1.248 | 0.652 |
| Q5SX39 | Myosin-4 OS=Mus musculus GN=Myh4 PE=2 SV=1 | 1.248 | 0.652 |
| Q9D0K2 | Succinyl-CoA:3-ketoacid coenzyme A transferase 1, mitochondrial OS=Mus musculus GN=Oxct1 PE=1 SV=1 | 1.249 | 0.650 |
| Q4VAE6 | Ras family member A OS=Mus musculus GN=Rhoa PE=1 SV=1 | 1.25 | 0.649 |
| P52480-2 | Isoform M1 of Pyruvate kinase PKM OS=Mus musculus GN=Pkm | 1.25 | 0.649 |
| Q9R069 | Basal cell adhesion molecule OS=Mus musculus GN=Bcam PE=1 SV=1 | 1.251 | 0.648 |
| Q8VDJ3 | Vigilin OS=Mus musculus GN=Hdlbp PE=1 SV=1 | 1.252 | 0.646 |
| O08600 | Endonuclease G, mitochondrial OS=Mus musculus GN=Endog PE=1 SV=1 | 1.253 | 0.644 |
| E9Q1W3 | Protein Neb OS=Mus musculus GN=Neb PE=1 SV=1 | 1.253 | 0.645 |
| Q8CAQ8-5 | Isoform 5 of MICOS complex subunit Mic60 OS=Mus musculus GN=Immt | 1.253 | 0.645 |
| E0CZ58 | Proteoglycan 4 OS=Mus musculus GN=Prg4 PE=1 SV=1 | 1.253 | 0.645 |
| P04104 | Keratin, type II cytoskeletal 1 OS=Mus musculus GN=Krt1 PE=1 SV=4 | 1.255 | 0.642 |
| P54775 | 26S protease regulatory subunit 6B OS=Mus musculus GN=Psmc4 PE=1 SV=2 | 1.255 | 0.642 |
| Q9D6R2 | Isocitrate dehydrogenase [NAD] subunit alpha, mitochondrial OS=Mus musculus GN=Idh3a PE=1 SV=1 | 1.256 | 0.641 |
| Q61234 | Alpha-1-syntrophin OS=Mus musculus GN=Snta1 PE=1 SV=1 | 1.257 | 0.639 |
| O35295 | Transcriptional activator protein Pur-beta OS=Mus musculus GN=Purb PE=1 SV=3 | 1.258 | 0.638 |
| P16858 | Glyceraldehyde-3-phosphate dehydrogenase OS=Mus musculus GN=Gapdh PE=1 SV=2 | 1.259 | 0.637 |
| E9QQ93 | Xin actin-binding repeat-containing protein 1 OS=Mus musculus GN=Xirp1 PE=1 SV=1 | 1.26 | 0.635 |
| Q6P6L5 | Protein Mybpc1 OS=Mus musculus GN=Mybpc1 PE=1 SV=1 | 1.26 | 0.636 |
| Q9Z2U0 | Proteasome subunit alpha type-7 OS=Mus musculus GN=Psma7 PE=1 SV=1 | 1.262 | 0.634 |
| Q64314 | Hematopoietic progenitor cell antigen CD34 OS=Mus musculus GN=Cd34 PE=1 SV=1 | 1.262 | 0.634 |
| Q3UW66 | Sulfurtransferase OS=Mus musculus GN=Mpst PE=1 SV=1 | 1.263 | 0.631 |
| P63242 | Eukaryotic translation initiation factor 5A-1 OS=Mus musculus GN=Eif5a PE=1 SV=2 | 1.265 | 0.628 |
| E9Q3E2 | Synaptopodin OS=Mus musculus GN=Synpo PE=1 SV=1 | 1.266 | 0.628 |
| A0A0R4J1E2 | Elongation factor 1-delta OS=Mus musculus GN=Eef1d PE=1 SV=1 | 1.269 | 0.623 |
| Q9DCT8 | Cysteine-rich protein 2 OS=Mus musculus GN=Crip2 PE=1 SV=1 | 1.27 | 0.622 |
| Q9DCY1 | Peptidyl-prolyl cis-trans isomerase OS=Mus musculus GN=Ppib PE=1 SV=1 | 1.272 | 0.620 |
| Q60668 | Heterogeneous nuclear ribonucleoprotein D0 OS=Mus musculus GN=Hnrnpd PE=1 SV=2 | 1.274 | 0.617 |
| A2AQA9 | Protein Neb OS=Mus musculus GN=Neb PE=1 SV=1 | 1.276 | 0.614 |
| P50580 | Proliferation-associated protein 2G4 OS=Mus musculus GN=Pa2g4 PE=1 SV=3 | 1.28 | 0.610 |
| P26039 | Talin-1 OS=Mus musculus GN=Tln1 PE=1 SV=2 | 1.281 | 0.609 |
| P19096 | Fatty acid synthase OS=Mus musculus GN=Fasn PE=1 SV=2 | 1.288 | 0.600 |
| Q9CQA3 | Succinate dehydrogenase [ubiquinone] iron-sulfur subunit, mitochondrial OS=Mus musculus GN=Sdhb PE=1 SV=1 | 1.289 | 0.599 |
| O09111 | NADH dehydrogenase [ubiquinone] 1 beta subcomplex subunit 11, mitochondrial OS=Mus musculus GN=Ndufb11 PE=1 SV=2 | 1.291 | 0.596 |
| Q9Z204 | Heterogeneous nuclear ribonucleoproteins C1/C2 OS=Mus musculus GN=Hnrnpc PE=1 SV=1 | 1.292 | 0.594 |
| P13542 | Myosin-8 OS=Mus musculus GN=Myh8 PE=2 SV=2 | 1.293 | 0.594 |
| Q4FJX4 | Csrp1 protein OS=Mus musculus GN=Csrp1 PE=1 SV=1 | 1.294 | 0.592 |
| P17742 | Peptidyl-prolyl cis-trans isomerase A OS=Mus musculus GN=Ppia PE=1 SV=2 | 1.295 | 0.590 |
| Q2HZ94 | Macrophage mannose receptor 1 OS=Mus musculus GN=Mrc1 PE=1 SV=1 | 1.3 | 0.585 |
| E9PWQ3 | Protein Col6a3 OS=Mus musculus GN=Col6a3 PE=1 SV=2 | 1.301 | 0.583 |
| Q8BP67 | 60S ribosomal protein L24 OS=Mus musculus GN=Rpl24 PE=1 SV=2 | 1.301 | 0.584 |
| Q9CZX8 | 40S ribosomal protein S19 OS=Mus musculus GN=Rps19 PE=1 SV=3 | 1.302 | 0.582 |
| P37804 | Transgelin OS=Mus musculus GN=Tagln PE=1 SV=3 | 1.306 | 0.577 |
| Q8VIJ6 | Splicing factor, proline- and glutamine-rich OS=Mus musculus GN=Sfpq PE=1 SV=1 | 1.309 | 0.574 |
| Q9D1A2 | Cytosolic non-specific dipeptidase OS=Mus musculus GN=Cndp2 PE=1 SV=1 | 1.31 | 0.572 |
| G5E814 | MCG5603 OS=Mus musculus GN=Ndufa11 PE=1 SV=1 | 1.31 | 0.572 |
| O70250 | Phosphoglycerate mutase 2 OS=Mus musculus GN=Pgam2 PE=1 SV=3 | 1.312 | 0.570 |
| P99024 | Tubulin beta-5 chain OS=Mus musculus GN=Tubb5 PE=1 SV=1 | 1.312 | 0.570 |
| Q78IK4 | MICOS complex subunit Mic27 OS=Mus musculus GN=Apool PE=1 SV=1 | 1.313 | 0.569 |
| Q01853 | Transitional endoplasmic reticulum ATPase OS=Mus musculus GN=Vcp PE=1 SV=4 | 1.319 | 0.561 |
| Q8VDM4 | 26S proteasome non-ATPase regulatory subunit 2 OS=Mus musculus GN=Psmd2 PE=1 SV=1 | 1.321 | 0.559 |
| Q3UDP9 | Monocarboxylate transporter 4 OS=Mus musculus GN=Slc16a3 PE=1 SV=1 | 1.331 | 0.548 |
| Q542I9 | 26S protease regulatory subunit 4 OS=Mus musculus GN=Psmc1 PE=1 SV=1 | 1.337 | 0.539 |
| P20029 | 78 kDa glucose-regulated protein OS=Mus musculus GN=Hspa5 PE=1 SV=3 | 1.34 | 0.537 |
| P40124 | Adenylyl cyclase-associated protein 1 OS=Mus musculus GN=Cap1 PE=1 SV=4 | 1.34 | 0.537 |
| Q6PHQ9 | Polyadenylate-binding protein OS=Mus musculus GN=Pabpc4 PE=1 SV=1 | 1.342 | 0.535 |
| A0A0R4J0Q5 | Lamin-B2 OS=Mus musculus GN=Lmnb2 PE=1 SV=1 | 1.344 | 0.532 |
| Q80UY1 | Carnosine N-methyltransferase OS=Mus musculus GN=Carnmt1 PE=1 SV=1 | 1.345 | 0.531 |
| Q04857 | Collagen alpha-1(VI) chain OS=Mus musculus GN=Col6a1 PE=1 SV=1 | 1.349 | 0.526 |
| Q8CGY6 | Protein unc-45 homolog B OS=Mus musculus GN=Unc45b PE=1 SV=1 | 1.351 | 0.525 |
| Q62318 | Transcription intermediary factor 1-beta OS=Mus musculus GN=Trim28 PE=1 SV=3 | 1.356 | 0.519 |
| P06728 | Apolipoprotein A-IV OS=Mus musculus GN=Apoa4 PE=1 SV=3 | 1.359 | 0.515 |
| A2A545 | Calcium channel, voltage-dependent, beta 1 subunit, isoform CRA_b OS=Mus musculus GN=Cacnb1 PE=1 SV=1 | 1.362 | 0.512 |
| Q4U4S6 | Xin actin-binding repeat-containing protein 2 OS=Mus musculus GN=Xirp2 PE=1 SV=1 | 1.363 | 0.511 |
| Q60936 | Atypical kinase ADCK3, mitochondrial OS=Mus musculus GN=Adck3 PE=1 SV=2 | 1.364 | 0.509 |
| Q0PD67 | RAB1, member RAS oncogene family, isoform CRA_a OS=Mus musculus GN=Rab1a PE=1 SV=1 | 1.364 | 0.509 |
| G5E902 | MCG10343, isoform CRA_b OS=Mus musculus GN=Slc25a3 PE=1 SV=1 | 1.366 | 0.507 |
| P62631 | Elongation factor 1-alpha 2 OS=Mus musculus GN=Eef1a2 PE=1 SV=1 | 1.367 | 0.506 |
| E9QQ25 | Striated muscle-specific serine/threonine-protein kinase OS=Mus musculus GN=Speg PE=1 SV=1 | 1.37 | 0.503 |
| E9Q8K5 | Titin OS=Mus musculus GN=Ttn PE=1 SV=1 | 1.372 | 0.501 |
| Q8CIB5 | Fermitin family homolog 2 OS=Mus musculus GN=Fermt2 PE=1 SV=1 | 1.372 | 0.501 |
| P52480 | Pyruvate kinase PKM OS=Mus musculus GN=Pkm PE=1 SV=4 | 1.377 | 0.495 |
| Q9QUR6 | Prolyl endopeptidase OS=Mus musculus GN=Prep PE=1 SV=1 | 1.377 | 0.496 |
| P97855 | Ras GTPase-activating protein-binding protein 1 OS=Mus musculus GN=G3bp1 PE=1 SV=1 | 1.378 | 0.494 |
| Q3T9Z2 | Glyoxylate reductase/hydroxypyruvate reductase OS=Mus musculus GN=Grhpr PE=1 SV=1 | 1.379 | 0.493 |
| P63017 | Heat shock cognate 71 kDa protein OS=Mus musculus GN=Hspa8 PE=1 SV=1 | 1.381 | 0.491 |
| Q76MZ3 | Serine/threonine-protein phosphatase 2A 65 kDa regulatory subunit A alpha isoform OS=Mus musculus GN=Ppp2r1a PE=1 SV=3 | 1.382 | 0.490 |
| P08249 | Malate dehydrogenase, mitochondrial OS=Mus musculus GN=Mdh2 PE=1 SV=3 | 1.384 | 0.487 |
| P09411 | Phosphoglycerate kinase 1 OS=Mus musculus GN=Pgk1 PE=1 SV=4 | 1.386 | 0.486 |
| Q922I7 | MCG13402, isoform CRA_c OS=Mus musculus GN=Ptbp1 PE=1 SV=1 | 1.39 | 0.481 |
| A0A0J9YUL3 | Septin 11, isoform CRA_b OS=Mus musculus GN=Sept11 PE=1 SV=1 | 1.39 | 0.482 |
| Q5M9M4 | 40S ribosomal protein S15a OS=Mus musculus GN=Rps15a PE=1 SV=1 | 1.393 | 0.478 |
| A0A087WSN6 | Fibronectin OS=Mus musculus GN=Fn1 PE=1 SV=1 | 1.394 | 0.477 |
| P82349 | Beta-sarcoglycan OS=Mus musculus GN=Sgcb PE=1 SV=1 | 1.394 | 0.477 |
| Q60864 | Stress-induced-phosphoprotein 1 OS=Mus musculus GN=Stip1 PE=1 SV=1 | 1.395 | 0.476 |
| E9PWE8 | Dihydropyrimidinase-related protein 3 OS=Mus musculus GN=Dpysl3 PE=1 SV=1 | 1.395 | 0.476 |
| P97443 | Histone-lysine N-methyltransferase Smyd1 OS=Mus musculus GN=Smyd1 PE=1 SV=3 | 1.398 | 0.473 |
| Q91ZJ5 | UTP--glucose-1-phosphate uridylyltransferase OS=Mus musculus GN=Ugp2 PE=1 SV=3 | 1.399 | 0.472 |
| Q922D8 | C-1-tetrahydrofolate synthase, cytoplasmic OS=Mus musculus GN=Mthfd1 PE=1 SV=4 | 1.399 | 0.473 |
| Q546G4 | Albumin 1 OS=Mus musculus GN=Alb PE=1 SV=1 | 1.4 | 0.471 |
| O54724 | Polymerase I and transcript release factor OS=Mus musculus GN=Ptrf PE=1 SV=1 | 1.401 | 0.470 |
| Q6PA06 | Atlastin-2 OS=Mus musculus GN=Atl2 PE=1 SV=1 | 1.402 | 0.469 |
| Q4FJQ6 | Serine (Or cysteine) peptidase inhibitor, clade B, member 6a, isoform CRA_a OS=Mus musculus GN=Serpinb6a PE=1 SV=1 | 1.404 | 0.467 |
| Q61233 | Plastin-2 OS=Mus musculus GN=Lcp1 PE=1 SV=4 | 1.405 | 0.466 |
| P47911 | 60S ribosomal protein L6 OS=Mus musculus GN=Rpl6 PE=1 SV=3 | 1.406 | 0.464 |
| P40240 | CD9 antigen OS=Mus musculus GN=Cd9 PE=1 SV=2 | 1.406 | 0.465 |
| Q9DCT2 | NADH dehydrogenase [ubiquinone] iron-sulfur protein 3, mitochondrial OS=Mus musculus GN=Ndufs3 PE=1 SV=2 | 1.41 | 0.461 |
| P51859 | Hepatoma-derived growth factor OS=Mus musculus GN=Hdgf PE=1 SV=2 | 1.411 | 0.460 |
| P20108 | Thioredoxin-dependent peroxide reductase, mitochondrial OS=Mus musculus GN=Prdx3 PE=1 SV=1 | 1.414 | 0.457 |
| Q62009 | Periostin OS=Mus musculus GN=Postn PE=1 SV=2 | 1.42 | 0.451 |
| P56375 | Acylphosphatase-2 OS=Mus musculus GN=Acyp2 PE=1 SV=2 | 1.42 | 0.451 |
| Q91Z83 | Myosin-7 OS=Mus musculus GN=Myh7 PE=1 SV=1 | 1.421 | 0.450 |
| Q545M7 | Parvalbumin alpha OS=Mus musculus GN=Pvalb PE=1 SV=1 | 1.422 | 0.449 |
| Q545X8 | 40S ribosomal protein S4 OS=Mus musculus GN=Rps4x PE=1 SV=1 | 1.426 | 0.445 |
| G3X914 | Cullin 5 OS=Mus musculus GN=Cul5 PE=1 SV=1 | 1.433 | 0.438 |
| Q9DCW4 | Electron transfer flavoprotein subunit beta OS=Mus musculus GN=Etfb PE=1 SV=3 | 1.436 | 0.435 |
| Q8VHX6-2 | Isoform 2 of Filamin-C OS=Mus musculus GN=Flnc | 1.436 | 0.436 |
| Q9JKF1 | Ras GTPase-activating-like protein IQGAP1 OS=Mus musculus GN=Iqgap1 PE=1 SV=2 | 1.446 | 0.426 |
| M0QW57 | Junctional sarcoplasmic reticulum protein 1 OS=Mus musculus GN=Jsrp1 PE=1 SV=1 | 1.447 | 0.425 |
| F6YVP7 | Protein Gm10260 OS=Mus musculus GN=Gm10260 PE=3 SV=2 | 1.448 | 0.423 |
| P15626 | Glutathione S-transferase Mu 2 OS=Mus musculus GN=Gstm2 PE=1 SV=2 | 1.453 | 0.419 |
| O35452 | Protein Tnxb OS=Mus musculus GN=Tnxb PE=1 SV=1 | 1.455 | 0.417 |
| Q8R146 | Acylamino-acid-releasing enzyme OS=Mus musculus GN=Apeh PE=1 SV=3 | 1.456 | 0.417 |
| Q9WUK2 | Eukaryotic translation initiation factor 4H OS=Mus musculus GN=Eif4h PE=1 SV=3 | 1.457 | 0.416 |
| Q3UKR1 | Decorin OS=Mus musculus GN=Dcn PE=1 SV=1 | 1.46 | 0.412 |
| Q6ZWV3 | 60S ribosomal protein L10 OS=Mus musculus GN=Rpl10 PE=1 SV=3 | 1.461 | 0.412 |
| Q3TVW6 | LIM and cysteine-rich domains 1 OS=Mus musculus GN=Lmcd1 PE=1 SV=1 | 1.466 | 0.407 |
| P70195 | Proteasome subunit beta type-7 OS=Mus musculus GN=Psmb7 PE=1 SV=1 | 1.467 | 0.406 |
| P11438 | Lysosome-associated membrane glycoprotein 1 OS=Mus musculus GN=Lamp1 PE=1 SV=2 | 1.474 | 0.400 |
| P21550 | Beta-enolase OS=Mus musculus GN=Eno3 PE=1 SV=3 | 1.478 | 0.397 |
| Q3UDC3 | Target of Myb protein 1 OS=Mus musculus GN=Tom1 PE=1 SV=1 | 1.481 | 0.394 |
| P19324 | Serpin H1 OS=Mus musculus GN=Serpinh1 PE=1 SV=3 | 1.484 | 0.391 |
| J3QNY1 | Protein Gm9242 OS=Mus musculus GN=Gm9242 PE=4 SV=1 | 1.487 | 0.389 |
| Q5SVJ0 | Calcium/calmodulin-dependent protein kinase II, beta, isoform CRA_b OS=Mus musculus GN=Camk2b PE=1 SV=1 | 1.488 | 0.388 |
| Q3UL22 | Chaperonin subunit 8 (Theta), isoform CRA_a OS=Mus musculus GN=Cct8 PE=1 SV=1 | 1.493 | 0.384 |
| P14602 | Heat shock protein beta-1 OS=Mus musculus GN=Hspb1 PE=1 SV=3 | 1.511 | 0.368 |
| P13707 | Glycerol-3-phosphate dehydrogenase [NAD(+)], cytoplasmic OS=Mus musculus GN=Gpd1 PE=1 SV=3 | 1.516 | 0.364 |
| J3QPZ9 | Beta-enolase (Fragment) OS=Mus musculus GN=Eno3 PE=1 SV=1 | 1.518 | 0.363 |
| F6RDH3 | Protein Neb (Fragment) OS=Mus musculus GN=Neb PE=1 SV=7 | 1.521 | 0.360 |
| Q3TT94 | Serine/threonine-protein phosphatase 2A 55 kDa regulatory subunit B OS=Mus musculus GN=Ppp2r2a PE=1 SV=1 | 1.526 | 0.356 |
| Q60675 | Laminin subunit alpha-2 OS=Mus musculus GN=Lama2 PE=1 SV=2 | 1.526 | 0.356 |
| P70302 | Stromal interaction molecule 1 OS=Mus musculus GN=Stim1 PE=1 SV=2 | 1.531 | 0.352 |
| Q8R5C5 | Beta-centractin OS=Mus musculus GN=Actr1b PE=1 SV=1 | 1.536 | 0.348 |
| P50608 | Fibromodulin OS=Mus musculus GN=Fmod PE=2 SV=1 | 1.54 | 0.345 |
| Q9R1P4 | Proteasome subunit alpha type-1 OS=Mus musculus GN=Psma1 PE=1 SV=1 | 1.547 | 0.339 |
| P70168 | Importin subunit beta-1 OS=Mus musculus GN=Kpnb1 PE=1 SV=2 | 1.547 | 0.340 |
| Q5XKE0 | Myosin-binding protein C, fast-type OS=Mus musculus GN=Mybpc2 PE=1 SV=1 | 1.548 | 0.339 |
| Q9WTR5 | Cadherin-13 OS=Mus musculus GN=Cdh13 PE=1 SV=2 | 1.55 | 0.337 |
| Q8C2Q8 | ATP synthase subunit gamma OS=Mus musculus GN=Atp5c1 PE=1 SV=1 | 1.553 | 0.335 |
| P63101 | 14-3-3 protein zeta/delta OS=Mus musculus GN=Ywhaz PE=1 SV=1 | 1.557 | 0.332 |
| P35979 | 60S ribosomal protein L12 OS=Mus musculus GN=Rpl12 PE=1 SV=2 | 1.569 | 0.323 |
| Q564E8 | 60S ribosomal protein L4 OS=Mus musculus GN=Rpl4 PE=1 SV=1 | 1.571 | 0.322 |
| Q8BU30 | Isoleucine--tRNA ligase, cytoplasmic OS=Mus musculus GN=Iars PE=1 SV=2 | 1.58 | 0.315 |
| P16045 | Galectin-1 OS=Mus musculus GN=Lgals1 PE=1 SV=3 | 1.595 | 0.305 |
| Q8VEK3 | Heterogeneous nuclear ribonucleoprotein U OS=Mus musculus GN=Hnrnpu PE=1 SV=1 | 1.602 | 0.300 |
| P68040 | Receptor of activated protein C kinase 1 OS=Mus musculus GN=Rack1 PE=1 SV=3 | 1.603 | 0.299 |
| Q3UTJ2-2 | Isoform 2 of Sorbin and SH3 domain-containing protein 2 OS=Mus musculus GN=Sorbs2 | 1.612 | 0.293 |
| Q9CR57 | 60S ribosomal protein L14 OS=Mus musculus GN=Rpl14 PE=1 SV=3 | 1.614 | 0.291 |
| P97449 | Aminopeptidase N OS=Mus musculus GN=Anpep PE=1 SV=4 | 1.616 | 0.291 |
| P14148 | 60S ribosomal protein L7 OS=Mus musculus GN=Rpl7 PE=1 SV=2 | 1.624 | 0.285 |
| Q8QZS1 | 3-hydroxyisobutyryl-CoA hydrolase, mitochondrial OS=Mus musculus GN=Hibch PE=1 SV=1 | 1.624 | 0.285 |
| Q543N3 | LIM and SH3 domain protein 1 OS=Mus musculus GN=Lasp1 PE=1 SV=1 | 1.629 | 0.282 |
| B0QZL1 | Alpha-enolase (Fragment) OS=Mus musculus GN=Eno1 PE=1 SV=1 | 1.635 | 0.278 |
| Q9D1R9 | 60S ribosomal protein L34 OS=Mus musculus GN=Rpl34 PE=1 SV=2 | 1.636 | 0.278 |
| P28271 | Cytoplasmic aconitate hydratase OS=Mus musculus GN=Aco1 PE=1 SV=3 | 1.641 | 0.274 |
| P09103 | Protein disulfide-isomerase OS=Mus musculus GN=P4hb PE=1 SV=2 | 1.644 | 0.272 |
| A0A0G2JFH2 | Microtubule-associated protein (Fragment) OS=Mus musculus GN=Map4 PE=1 SV=1 | 1.645 | 0.272 |
| O08539-2 | Isoform 2 of Myc box-dependent-interacting protein 1 OS=Mus musculus GN=Bin1 | 1.649 | 0.269 |
| P58774-2 | Isoform 2 of Tropomyosin beta chain OS=Mus musculus GN=Tpm2 | 1.651 | 0.268 |
| Q9DBG6 | Dolichyl-diphosphooligosaccharide--protein glycosyltransferase subunit 2 OS=Mus musculus GN=Rpn2 PE=1 SV=1 | 1.656 | 0.265 |
| D3Z7H8 | Protein Cilp2 OS=Mus musculus GN=Cilp2 PE=1 SV=1 | 1.659 | 0.263 |
| Q8CI12 | Smoothelin-like protein 2 OS=Mus musculus GN=Smtnl2 PE=1 SV=1 | 1.663 | 0.261 |
| Q9DB60 | Prostamide/prostaglandin F synthase OS=Mus musculus GN=Fam213b PE=1 SV=1 | 1.671 | 0.256 |
| Q8BRK8 | 5'-AMP-activated protein kinase catalytic subunit alpha-2 OS=Mus musculus GN=Prkaa2 PE=1 SV=3 | 1.672 | 0.255 |
| A2ABU4 | Myomesin-3 OS=Mus musculus GN=Myom3 PE=1 SV=1 | 1.676 | 0.253 |
| Q61102 | ATP-binding cassette sub-family B member 7, mitochondrial OS=Mus musculus GN=Abcb7 PE=1 SV=3 | 1.677 | 0.253 |
| Q3UI33 | Methionine aminopeptidase 2 OS=Mus musculus GN=Metap2 PE=1 SV=1 | 1.679 | 0.252 |
| P24270 | Catalase OS=Mus musculus GN=Cat PE=1 SV=4 | 1.681 | 0.250 |
| Q91VR5 | ATP-dependent RNA helicase DDX1 OS=Mus musculus GN=Ddx1 PE=1 SV=1 | 1.684 | 0.249 |
| P62849 | 40S ribosomal protein S24 OS=Mus musculus GN=Rps24 PE=1 SV=1 | 1.686 | 0.248 |
| E9PY39 | Protein Gm20431 OS=Mus musculus GN=Gm20431 PE=4 SV=1 | 1.69 | 0.245 |
| Q3UEK9 | Alpha-2-HS-glycoprotein OS=Mus musculus GN=Ahsg PE=1 SV=1 | 1.691 | 0.245 |
| Q9DCZ1 | GMP reductase 1 OS=Mus musculus GN=Gmpr PE=1 SV=1 | 1.694 | 0.243 |
| G3UXL2 | Protein Prps1l3 OS=Mus musculus GN=Prps1l3 PE=3 SV=1 | 1.7 | 0.240 |
| E9QAZ2 | Ribosomal protein L15 OS=Mus musculus GN=Gm10020 PE=3 SV=1 | 1.701 | 0.239 |
| P40936 | Indolethylamine N-methyltransferase OS=Mus musculus GN=Inmt PE=1 SV=1 | 1.706 | 0.236 |
| Q99NF7 | Ppm1b protein OS=Mus musculus GN=Ppm1b PE=1 SV=1 | 1.72 | 0.229 |
| P48678 | Prelamin-A/C OS=Mus musculus GN=Lmna PE=1 SV=2 | 1.721 | 0.228 |
| Q5BLK1 | 40S ribosomal protein S6 OS=Mus musculus GN=Rps6 PE=1 SV=1 | 1.73 | 0.223 |
| E9Q447 | Spectrin alpha chain, non-erythrocytic 1 OS=Mus musculus GN=Sptan1 PE=1 SV=1 | 1.73 | 0.224 |
| P14142 | Solute carrier family 2, facilitated glucose transporter member 4 OS=Mus musculus GN=Slc2a4 PE=1 SV=3 | 1.737 | 0.220 |
| P52503 | NADH dehydrogenase [ubiquinone] iron-sulfur protein 6, mitochondrial OS=Mus musculus GN=Ndufs6 PE=1 SV=2 | 1.739 | 0.219 |
| Q62351 | Transferrin receptor protein 1 OS=Mus musculus GN=Tfrc PE=1 SV=1 | 1.741 | 0.218 |
| P19246 | Neurofilament heavy polypeptide OS=Mus musculus GN=Nefh PE=1 SV=3 | 1.776 | 0.201 |
| Q5M9L7 | 40S ribosomal protein S17 OS=Mus musculus GN=Rps17 PE=1 SV=1 | 1.778 | 0.200 |
| Q9QY80 | Very-long-chain (3R)-3-hydroxyacyl-CoA dehydratase 1 OS=Mus musculus GN=Hacd1 PE=2 SV=1 | 1.78 | 0.199 |
| O08583 | THO complex subunit 4 OS=Mus musculus GN=Alyref PE=1 SV=3 | 1.789 | 0.195 |
| Q99LB4 | Capping protein (Actin filament), gelsolin-like OS=Mus musculus GN=Capg PE=1 SV=1 | 1.795 | 0.192 |
| Q9CQW2 | ADP-ribosylation factor-like protein 8B OS=Mus musculus GN=Arl8b PE=1 SV=1 | 1.801 | 0.190 |
| P09541 | Myosin light chain 4 OS=Mus musculus GN=Myl4 PE=1 SV=3 | 1.801 | 0.190 |
| Q9DBC7 | cAMP-dependent protein kinase type I-alpha regulatory subunit OS=Mus musculus GN=Prkar1a PE=1 SV=3 | 1.805 | 0.188 |
| Q9DBG5 | Perilipin-3 OS=Mus musculus GN=Plin3 PE=1 SV=1 | 1.805 | 0.188 |
| P14685 | 26S proteasome non-ATPase regulatory subunit 3 OS=Mus musculus GN=Psmd3 PE=1 SV=3 | 1.806 | 0.188 |
| Q9JJW5 | Myozenin-2 OS=Mus musculus GN=Myoz2 PE=1 SV=1 | 1.82 | 0.182 |
| Q61292 | Laminin subunit beta-2 OS=Mus musculus GN=Lamb2 PE=1 SV=2 | 1.825 | 0.180 |
| A2ASS6-3 | Isoform 3 of Titin OS=Mus musculus GN=Ttn | 1.851 | 0.169 |
| Q3TLP8 | RAS-related C3 botulinum substrate 1, isoform CRA_a OS=Mus musculus GN=Rac1 PE=1 SV=1 | 1.854 | 0.168 |
| S4R2K9 | Ankyrin-3 (Fragment) OS=Mus musculus GN=Ank3 PE=1 SV=1 | 1.856 | 0.167 |
| Q9Z1N5 | Spliceosome RNA helicase Ddx39b OS=Mus musculus GN=Ddx39b PE=1 SV=1 | 1.861 | 0.165 |
| Q9D154 | Leukocyte elastase inhibitor A OS=Mus musculus GN=Serpinb1a PE=1 SV=1 | 1.861 | 0.165 |
| Q5YLW3 | 40S ribosomal protein S3 OS=Mus musculus GN=Rps3 PE=1 SV=1 | 1.864 | 0.164 |
| Q7TMM9 | Tubulin beta-2A chain OS=Mus musculus GN=Tubb2a PE=1 SV=1 | 1.89 | 0.154 |
| Q4FK49 | Inorganic pyrophosphatase OS=Mus musculus GN=Ppa1 PE=1 SV=1 | 1.899 | 0.151 |
| Q3UR55 | Sodium/potassium-transporting ATPase subunit beta OS=Mus musculus GN=Atp1b2 PE=1 SV=1 | 1.904 | 0.149 |
| Q00897 | Alpha-1-antitrypsin 1-4 OS=Mus musculus GN=Serpina1d PE=1 SV=1 | 1.91 | 0.147 |
| Q02788 | Collagen alpha-2(VI) chain OS=Mus musculus GN=Col6a2 PE=1 SV=3 | 1.912 | 0.146 |
| B2RUC7 | Serine-threonine kinase receptor-associated protein OS=Mus musculus GN=Strap PE=1 SV=1 | 1.933 | 0.139 |
| Q9DC69 | NADH dehydrogenase [ubiquinone] 1 alpha subcomplex subunit 9, mitochondrial OS=Mus musculus GN=Ndufa9 PE=1 SV=2 | 1.944 | 0.136 |
| Q9ERS2 | NADH dehydrogenase [ubiquinone] 1 alpha subcomplex subunit 13 OS=Mus musculus GN=Ndufa13 PE=1 SV=3 | 1.947 | 0.135 |
| F8WIA1 | CAP-Gly domain-containing linker protein 1 OS=Mus musculus GN=Clip1 PE=1 SV=1 | 1.951 | 0.134 |
| Q3U1J4 | DNA damage-binding protein 1 OS=Mus musculus GN=Ddb1 PE=1 SV=2 | 1.961 | 0.131 |
| O54734 | Dolichyl-diphosphooligosaccharide--protein glycosyltransferase 48 kDa subunit OS=Mus musculus GN=Ddost PE=1 SV=2 | 1.967 | 0.129 |
| Q00623 | Apolipoprotein A-I OS=Mus musculus GN=Apoa1 PE=1 SV=2 | 1.97 | 0.128 |
| Q99KQ4 | Nicotinamide phosphoribosyltransferase OS=Mus musculus GN=Nampt PE=1 SV=1 | 1.972 | 0.128 |
| Q5BLJ9 | 60S ribosomal protein L27 OS=Mus musculus GN=Rpl27 PE=1 SV=1 | 1.978 | 0.126 |
| Q05CI8 | Ccbl1 protein OS=Mus musculus GN=Ccbl1 PE=1 SV=1 | 2 | 0.119 |
| Q9DCD0 | 6-phosphogluconate dehydrogenase, decarboxylating OS=Mus musculus GN=Pgd PE=1 SV=3 | 2.022 | 0.113 |
| P54823 | Probable ATP-dependent RNA helicase DDX6 OS=Mus musculus GN=Ddx6 PE=1 SV=1 | 2.026 | 0.113 |
| Q3TVK3 | Aspartyl aminopeptidase OS=Mus musculus GN=Dnpep PE=1 SV=1 | 2.042 | 0.108 |
| P47963 | 60S ribosomal protein L13 OS=Mus musculus GN=Rpl13 PE=1 SV=3 | 2.045 | 0.108 |
| Q99KF1 | Transmembrane emp24 domain-containing protein 9 OS=Mus musculus GN=Tmed9 PE=1 SV=2 | 2.048 | 0.107 |
| Q9D8N0 | Elongation factor 1-gamma OS=Mus musculus GN=Eef1g PE=1 SV=3 | 2.048 | 0.107 |
| Q8R086 | Sulfite oxidase, mitochondrial OS=Mus musculus GN=Suox PE=1 SV=2 | 2.055 | 0.105 |
| Q9CXI0 | 2-methoxy-6-polyprenyl-1,4-benzoquinol methylase, mitochondrial OS=Mus musculus GN=Coq5 PE=1 SV=2 | 2.059 | 0.104 |
| P10493 | Nidogen-1 OS=Mus musculus GN=Nid1 PE=1 SV=2 | 2.06 | 0.104 |
| A2AQ53 | Fibrillin-1 OS=Mus musculus GN=Fbn1 PE=1 SV=1 | 2.066 | 0.102 |
| Q543J5 | Antithrombin OS=Mus musculus GN=Serpinc1 PE=1 SV=1 | 2.098 | 0.095 |
| Q9ESL4-2 | Isoform 2 of Mitogen-activated protein kinase kinase kinase MLT OS=Mus musculus GN=Zak | 2.114 | 0.091 |
| P97429 | Annexin A4 OS=Mus musculus GN=Anxa4 PE=1 SV=4 | 2.119 | 0.090 |
| Q4VAA2 | Protein CDV3 OS=Mus musculus GN=Cdv3 PE=1 SV=2 | 2.121 | 0.090 |
| Q8CDN6 | Thioredoxin-like protein 1 OS=Mus musculus GN=Txnl1 PE=1 SV=3 | 2.124 | 0.090 |
| Q99JI6 | Ras-related protein Rap-1b OS=Mus musculus GN=Rap1b PE=1 SV=2 | 2.153 | 0.084 |
| Q9ERD7 | Tubulin beta-3 chain OS=Mus musculus GN=Tubb3 PE=1 SV=1 | 2.156 | 0.083 |
| P97384 | Annexin A11 OS=Mus musculus GN=Anxa11 PE=1 SV=2 | 2.173 | 0.080 |
| P11087 | Collagen alpha-1(I) chain OS=Mus musculus GN=Col1a1 PE=1 SV=4 | 2.25 | 0.067 |
| P62843 | 40S ribosomal protein S15 OS=Mus musculus GN=Rps15 PE=1 SV=2 | 2.258 | 0.066 |
| F6X9B6 | Protein Neb (Fragment) OS=Mus musculus GN=Neb PE=1 SV=1 | 2.28 | 0.062 |
| Q91YH5 | Atlastin-3 OS=Mus musculus GN=Atl3 PE=1 SV=1 | 2.282 | 0.062 |
| Q62465 | Synaptic vesicle membrane protein VAT-1 homolog OS=Mus musculus GN=Vat1 PE=1 SV=3 | 2.322 | 0.057 |
| Q99KK7 | Dipeptidyl peptidase 3 OS=Mus musculus GN=Dpp3 PE=1 SV=2 | 2.331 | 0.055 |
| Q3UAD6 | Endoplasmin OS=Mus musculus GN=Hsp90b1 PE=1 SV=1 | 2.387 | 0.049 |
| Q9CWJ9 | Bifunctional purine biosynthesis protein PURH OS=Mus musculus GN=Atic PE=1 SV=2 | 2.397 | 0.048 |
| Q9JKB3-2 | Isoform 2 of Y-box-binding protein 3 OS=Mus musculus GN=Ybx3 | 2.508 | 0.037 |
| E9PUM4 | Talin-2 OS=Mus musculus GN=Tln2 PE=1 SV=1 | 2.541 | 0.034 |
| P62830 | 60S ribosomal protein L23 OS=Mus musculus GN=Rpl23 PE=1 SV=1 | 2.559 | 0.033 |
| A4QPC5 | Chymase OS=Mus musculus GN=Cma1 PE=1 SV=1 | 2.563 | 0.032 |
| E9QK41 | Actin-binding LIM protein 1 OS=Mus musculus GN=Ablim1 PE=1 SV=1 | 2.61 | 0.029 |
| Q99LX0 | Protein deglycase DJ-1 OS=Mus musculus GN=Park7 PE=1 SV=1 | 2.661 | 0.026 |
| A0A0G2JGL0 | Ubiquitin-conjugating enzyme E2 D3 OS=Mus musculus GN=Ube2d3 PE=1 SV=1 | 2.753 | 0.021 |
| P97298 | Pigment epithelium-derived factor OS=Mus musculus GN=Serpinf1 PE=1 SV=2 | 2.763 | 0.021 |
| Q5M9L0 | Eukaryotic translation initiation factor 3 subunit H OS=Mus musculus GN=Eif3h PE=1 SV=1 | 2.824 | 0.018 |
| K3W4R4 | Collagen alpha-1(XIV) chain OS=Mus musculus GN=Col14a1 PE=1 SV=1 | 2.841 | 0.017 |
| Q70IV5-2 | Isoform 2 of Synemin OS=Mus musculus GN=Synm | 2.859 | 0.017 |
| P22752 | Histone H2A type 1 OS=Mus musculus GN=Hist1h2ab PE=1 SV=3 | 2.956 | 0.013 |
| P62301 | 40S ribosomal protein S13 OS=Mus musculus GN=Rps13 PE=1 SV=2 | 2.965 | 0.013 |
| P56391 | Cytochrome c oxidase subunit 6B1 OS=Mus musculus GN=Cox6b1 PE=1 SV=2 | 3.189 | 0.008 |
| P62334 | 26S protease regulatory subunit 10B OS=Mus musculus GN=Psmc6 PE=1 SV=1 | 3.239 | 0.007 |
| Q78PY7 | Staphylococcal nuclease domain-containing protein 1 OS=Mus musculus GN=Snd1 PE=1 SV=1 | 3.329 | 0.006 |
| Q6ZQI3 | Malectin OS=Mus musculus GN=Mlec PE=1 SV=2 | 3.441 | 0.005 |
| Q9JHW2 | Omega-amidase NIT2 OS=Mus musculus GN=Nit2 PE=1 SV=1 | 3.443 | 0.005 |
| Q3TML0 | Protein disulfide-isomerase A6 OS=Mus musculus GN=Pdia6 PE=1 SV=1 | 3.456 | 0.005 |
| P62071 | Ras-related protein R-Ras2 OS=Mus musculus GN=Rras2 PE=1 SV=1 | 3.538 | 0.004 |
| Q3ULJ0 | Glycerol-3-phosphate dehydrogenase 1-like protein OS=Mus musculus GN=Gpd1l PE=1 SV=2 | 3.597 | 0.003 |
| P97927 | Laminin subunit alpha-4 OS=Mus musculus GN=Lama4 PE=1 SV=2 | 3.78 | 0.002 |
| P62137 | Serine/threonine-protein phosphatase PP1-alpha catalytic subunit OS=Mus musculus GN=Ppp1ca PE=1 SV=1 | 4.206 | 0.001 |
| D3YTQ3 | Heterogeneous nuclear ribonucleoprotein D-like OS=Mus musculus GN=Hnrnpdl PE=1 SV=1 | 4.393 | 0.001 |
| P17427 | AP-2 complex subunit alpha-2 OS=Mus musculus GN=Ap2a2 PE=1 SV=2 | 4.492 | 0.001 |
| P42208 | Septin-2 OS=Mus musculus GN=Sept2 PE=1 SV=2 | 4.559 | 0.000 |
| Q3U4U6 | T-complex protein 1 subunit gamma OS=Mus musculus GN=Cct3 PE=1 SV=1 | 4.736 | 0.000 |
| G3X9D8 | Kelch-like protein 31 OS=Mus musculus GN=Klhl31 PE=1 SV=1 | 5.217 | 0.000 |
| Q6ZWZ4 | 60S ribosomal protein L36 OS=Mus musculus GN=Rpl36 PE=1 SV=1 | 5.354 | 0.000 |
| Q545F5 | ATP synthase subunit epsilon, mitochondrial OS=Mus musculus GN=Atp5e PE=1 SV=1 | 6.057 | 0.000 |
| Q8BFS6 | Serine/threonine-protein phosphatase CPPED1 OS=Mus musculus GN=Cpped1 PE=1 SV=1 | 6.121 | 0.000 |
| Q08091 | Calponin-1 OS=Mus musculus GN=Cnn1 PE=1 SV=1 | 6.191 | 0.000 |
| P14131 | 40S ribosomal protein S16 OS=Mus musculus GN=Rps16 PE=1 SV=4 | 6.201 | 0.000 |
| Q9ERK4 | Exportin-2 OS=Mus musculus GN=Cse1l PE=1 SV=1 | 6.548 | 0.000 |
| Q3UAI3 | CD36 antigen, isoform CRA_a OS=Mus musculus GN=Cd36 PE=1 SV=1 | 6.736 | 0.000 |
| O35643 | AP-1 complex subunit beta-1 OS=Mus musculus GN=Ap1b1 PE=1 SV=2 | 8.253 | 0.000 |
| Q9CQ75 | NADH dehydrogenase [ubiquinone] 1 alpha subcomplex subunit 2 OS=Mus musculus GN=Ndufa2 PE=1 SV=3 | 9.921 | 0.000 |
| A0A0R4J093 | UMP-CMP kinase OS=Mus musculus GN=Cmpk1 PE=1 SV=1 | 12.708 | 0.000 |
| Q9JK92 | Heat shock protein beta-8 OS=Mus musculus GN=Hspb8 PE=1 SV=1 | 20.534 | 0.000 |
| P02089 | Hemoglobin subunit beta-2 OS=Mus musculus GN=Hbb-b2 PE=1 SV=2 | 37.122 | 0.000 |
| P02088 | Hemoglobin subunit beta-1 OS=Mus musculus GN=Hbb-b1 PE=1 SV=2 | 93.427 | 0.000 |
| P43025 | Tetranectin OS=Mus musculus GN=Clec3b PE=1 SV=2 | 100 | 0.000 |
