# Supplementary material for: Defects in sarcolemma repair and skeletal muscle function after injury in a mouse model of Niemann-Pick type A/B disease
Source: Skelet Muscle. 2019 Jan 5;9:1. doi: 10.1186/s13395-018-0187-5 (PMC6320626; doi:10.1186/s13395-018-0187-5)
Supplement: Supplementary file 5 — Table S4. Skeletal muscle master protein subset. (DOCX 57 kb) [file 13395_2018_187_MOESM5_ESM.docx]

**Table S4. Skeletal Muscle Master Protein Subset.**

Extracted from Uniprot database (https://www.uniprot.org/) using skeletal muscle annotation, plus supplementation with functionally important skeletal muscle proteins from the literature [29].

| **Uniprot Accession #** | **Description** | **Abundance Ratio: (QF, KO) / (QF, WT)** | **Abundance Ratio P-Value: (QF, KO) / (QF, WT)** | **Abundance Ratio: (TP, KO) / (TP, WT)** | **Abundance Ratio P-Value: (TP, KO) / (TP, WT)** | **Abundance Ratio: (FDB, KO) / (FDB, WT)** | **Abundance Ratio P-Value: (FDB, KO) / (FDB, WT)** |
| --- | --- | --- | --- | --- | --- | --- | --- |
| Q9JK42 | [Pyruvate dehydrogenase (acetyl-transferring)] kinase isozyme 2, mitochondrial OS=Mus musculus GN=Pdk2 PE=1 SV=2 | 0.828 | 0.844759 | 0.532 | 0.121932 | 0.653 | 0.361182 |
| P63323 | 40S ribosomal protein S12 OS=Mus musculus GN=Rps12 PE=1 SV=2 | 1.264 | 0.37881 | 1.108 | 0.863398 | 0.686 | 0.427684 |
| P14206 | 40S ribosomal protein SA OS=Mus musculus GN=Rpsa PE=1 SV=4 | 0.839 | 0.8709 | 0.616 | 0.22856 | 0.948 | 0.87451 |
| Q8BRK8 | 5'-AMP-activated protein kinase catalytic subunit alpha-2 OS=Mus musculus GN=Prkaa2 PE=1 SV=3 | 1.103 | 0.592857 | 1.672 | 0.255239 | 1.024 | 0.764292 |
| Q564E8 | 60S ribosomal protein L4 OS=Mus musculus GN=Rpl4 PE=1 SV=1 | 3.494 | 0.000531 | 1.571 | 0.321767 | 0.61 | 0.13596 |
| P68134 | Actin, alpha skeletal muscle OS=Mus musculus GN=Acta1 PE=1 SV=1 | 0.887 | 0.983956 | 1.049 | 0.965343 | 0.72 | 0.379372 |
| P62737 | Actin, aortic smooth muscle OS=Mus musculus GN=Acta2 PE=1 SV=1 | 1.029 | 0.721147 | 1.031 | 0.997221 | 1.002 | 0.715465 |
| E9QK41 | Actin-binding LIM protein 1 OS=Mus musculus GN=Ablim1 PE=1 SV=1 | 1.496 | 0.190617 | 2.61 | 0.029132 | 1.252 | 0.334863 |
| Q3ULT2 | Actinin alpha 4 OS=Mus musculus GN=Actn4 PE=1 SV=1 | 0.955 | 0.868117 | 0.846 | 0.64544 | 1.045 | 0.600698 |
| P61161 | Actin-related protein 2 OS=Mus musculus GN=Actr2 PE=1 SV=1 | 1.929 | 0.050692 | 0.662 | 0.300815 | 1.257 | 0.272833 |
| Q99JY9 | Actin-related protein 3 OS=Mus musculus GN=Actr3 PE=1 SV=3 | 0.703 | 0.541508 | 0.843 | 0.638853 | 1.022 | 0.696314 |
| Q60994 | Adiponectin OS=Mus musculus GN=Adipoq PE=1 SV=2 | 0.614 | 0.340327 | 0.581 | 0.180072 | 0.766 | 0.631133 |
| P48962 | ADP/ATP translocase 1 OS=Mus musculus GN=Slc25a4 PE=1 SV=4 | 0.581 | 0.272595 | 1.207 | 0.709508 | 0.557 | 0.06546 |
| P51881 | ADP/ATP translocase 2 OS=Mus musculus GN=Slc25a5 PE=1 SV=3 | 0.526 | 0.177444 | 1.013 | 0.969858 | 0.908 | 0.99655 |
| Q8CG76 | Aflatoxin B1 aldehyde reductase member 2 OS=Mus musculus GN=Akr7a2 PE=1 SV=3 | 1.203 | 0.450553 | 1.134 | 0.819908 | 0.657 | 0.324335 |
| P45376 | Aldose reductase OS=Mus musculus GN=Akr1b1 PE=1 SV=3 | 0.893 | 0.996271 | 0.814 | 0.582614 | 0.899 | 0.967326 |
| A1BN54 | Alpha actinin 1a OS=Mus musculus GN=Actn1 PE=1 SV=1 | 1.736 | 0.091672 | 0.787 | 0.529278 | 1.033 | 0.631333 |
| Q61234 | Alpha-1-syntrophin OS=Mus musculus GN=Snta1 PE=1 SV=1 | 1.072 | 0.644629 | 1.257 | 0.639312 | 0.949 | 0.873018 |
| Q9JI91 | Alpha-actinin-2 OS=Mus musculus GN=Actn2 PE=1 SV=2 | 0.847 | 0.889579 | 0.83 | 0.613978 | 0.965 | 0.823102 |
| O88990 | Alpha-actinin-3 OS=Mus musculus GN=Actn3 PE=2 SV=1 | 1.035 | 0.709187 | 0.979 | 0.905854 | 0.973 | 0.798943 |
| P82350 | Alpha-sarcoglycan OS=Mus musculus GN=Sgca PE=1 SV=1 | 1.077 | 0.635713 | 0.58 | 0.177925 | 1.325 | 0.226983 |
| P10107 | Annexin A1 OS=Mus musculus GN=Anxa1 PE=1 SV=2 | 0.893 | 0.996863 | 0.846 | 0.645324 | 0.496 | 0.022786 |
| P17426 | AP-2 complex subunit alpha-1 OS=Mus musculus GN=Ap2a1 PE=1 SV=1 | 1.767 | 0.083388 | 0.557 | 0.150062 | 0.35 | 0.008451 |
| A6H6K1 | Aspn protein OS=Mus musculus GN=Aspn PE=1 SV=1 | 0.556 | 0.226358 | 0.913 | 0.777696 | 0.743 | 0.446813 |
| Q60936 | Atypical kinase ADCK3, mitochondrial OS=Mus musculus GN=Adck3 PE=1 SV=2 | 1.037 | 0.706227 | 1.364 | 0.509096 | 1.005 | 0.706504 |
| P21550 | Beta-enolase OS=Mus musculus GN=Eno3 PE=1 SV=3 | 0.802 | 0.782697 | 1.478 | 0.396864 | 0.886 | 0.922429 |
| P82349 | Beta-sarcoglycan OS=Mus musculus GN=Sgcb PE=1 SV=1 | 0.99 | 0.796559 | 1.394 | 0.477423 | 1.165 | 0.351474 |
| Q8VBT1 | Beta-taxilin OS=Mus musculus GN=Txlnb PE=1 SV=2 | 1.618 | 0.131525 | 1.042 | 0.977954 | 0.954 | 0.865963 |
| Q9WV35 | C->U-editing enzyme APOBEC-2 OS=Mus musculus GN=Apobec2 PE=1 SV=1 | 1.054 | 0.676275 | 0.836 | 0.626046 | 0.729 | 0.405848 |
| Q923T9 | Calcium/calmodulin-dependent protein kinase type II subunit gamma OS=Mus musculus GN=Camk2g PE=1 SV=1 | 1.324 | 0.318381 | 0.974 | 0.896339 | 0.75 | 0.580026 |
| O35350 | Calpain-1 catalytic subunit OS=Mus musculus GN=Capn1 PE=1 SV=1 | 1.708 | 0.099923 | 0.892 | 0.736299 | 1.067 | 0.556645 |
| O08529 | Calpain-2 catalytic subunit OS=Mus musculus GN=Capn2 PE=1 SV=4 | 1.328 | 0.31463 | 1.043 | 0.97638 | 0.926 | 0.944846 |
| Q64691 | Calpain-3 OS=Mus musculus GN=Capn3 PE=2 SV=2 | 0.754 | 0.664893 | 0.643 | 0.269987 | 0.872 | 0.964949 |
| P51125 | Calpastatin OS=Mus musculus GN=Cast PE=1 SV=2 | 3.509 | 0.00051 | 0.723 | 0.406554 | 1.049 | 0.687571 |
| O09165 | Calsequestrin-1 OS=Mus musculus GN=Casq1 PE=1 SV=3 | 1.841 | 0.06639 | 0.708 | 0.379757 | 0.542 | 0.051816 |
| O09161 | Calsequestrin-2 OS=Mus musculus GN=Casq2 PE=1 SV=3 | 0.243 | 0.000918 | 0.101 | 4.95E-08 | 0.478 | 0.026428 |
| Q9DBC7 | cAMP-dependent protein kinase type I-alpha regulatory subunit OS=Mus musculus GN=Prkar1a PE=1 SV=3 | 0.881 | 0.971025 | 1.805 | 0.188035 | 0.804 | 0.801866 |
| P24270 | Catalase OS=Mus musculus GN=Cat PE=1 SV=4 | 1.978 | 0.04354 | 1.681 | 0.250055 | 0.972 | 0.83706 |
| P49817 | Caveolin-1 OS=Mus musculus GN=Cav1 PE=1 SV=1 | 0.61 | 0.331432 | 0.634 | 0.254841 | 1.354 | 0.134802 |
| Q91WS0 | CDGSH iron-sulfur domain-containing protein 1 OS=Mus musculus GN=Cisd1 PE=1 SV=1 | 0.62 | 0.351275 | 0.358 | 0.013211 | 0.789 | 0.76407 |
| Q8R4N0 | Citrate lyase subunit beta-like protein, mitochondrial OS=Mus musculus GN=Clybl PE=1 SV=2 | 0.737 | 0.624044 | 0.998 | 0.941359 | 1.272 | 0.299344 |
| P18760 | Cofilin-1 OS=Mus musculus GN=Cfl1 PE=1 SV=3 | 1.448 | 0.220084 | 0.566 | 0.161129 | 1.408 | 0.10069 |
| P45591 | Cofilin-2 OS=Mus musculus GN=Cfl2 PE=1 SV=1 | 0.829 | 0.847374 | 1.236 | 0.668743 | 0.866 | 0.854105 |
| O35206 | Collagen alpha-1(XV) chain OS=Mus musculus GN=Col15a1 PE=1 SV=2 | 0.608 | 0.327213 | 0.998 | 0.941854 | 0.945 | 0.88347 |
| Q02788 | Collagen alpha-2(VI) chain OS=Mus musculus GN=Col6a2 PE=1 SV=3 | 0.855 | 0.909923 | 1.912 | 0.146476 | 0.811 | 0.666199 |
| A6H584 | Collagen alpha-5(VI) chain OS=Mus musculus GN=Col6a5 PE=1 SV=4 | 1.655 | 0.117411 | 0.736 | 0.430658 | 1.125 | 0.577179 |
| P61202 | COP9 signalosome complex subunit 2 OS=Mus musculus GN=Cops2 PE=1 SV=1 | 1.245 | 0.399996 | 0.842 | 0.636894 | 0.54 | 0.233249 |
| Q04447 | Creatine kinase B-type OS=Mus musculus GN=Ckb PE=1 SV=1 | 2.954 | 0.002384 | 1.011 | 0.965622 | 0.825 | 0.715657 |
| P07310 | Creatine kinase M-type OS=Mus musculus GN=Ckm PE=1 SV=1 | 1.031 | 0.717856 | 1.238 | 0.665386 | 1.15 | 0.376846 |
| Q6P8J7 | Creatine kinase S-type, mitochondrial OS=Mus musculus GN=Ckmt2 PE=1 SV=1 | 0.855 | 0.908526 | 0.934 | 0.819099 | 1.149 | 0.379169 |
| Q4FJX4 | Csrp1 protein OS=Mus musculus GN=Csrp1 PE=1 SV=1 | 0.625 | 0.361711 | 1.294 | 0.591926 | 0.935 | 0.916862 |
| P50462 | Cysteine and glycine-rich protein 3 OS=Mus musculus GN=Csrp3 PE=1 SV=1 | 0.334 | 0.012407 | 1.162 | 0.776245 | 0.742 | 0.444404 |
| Q9DCT8 | Cysteine-rich protein 2 OS=Mus musculus GN=Crip2 PE=1 SV=1 | 1.123 | 0.561831 | 1.27 | 0.622334 | 1.305 | 0.174951 |
| Q9D020 | Cytosolic 5'-nucleotidase 3A OS=Mus musculus GN=Nt5c3a PE=1 SV=4 | 0.678 | 0.48116 | 1.191 | 0.73213 | 1.066 | 0.549415 |
| Q3UKR1 | Decorin OS=Mus musculus GN=Dcn PE=1 SV=1 | 0.527 | 0.179369 | 1.46 | 0.412235 | 1.02 | 0.666679 |
| P82347 | Delta-sarcoglycan OS=Mus musculus GN=Sgcd PE=1 SV=1 | 0.417 | 0.052743 | 1.082 | 0.906522 | 1.07 | 0.550561 |
| P31001 | Desmin OS=Mus musculus GN=Des PE=1 SV=3 | 0.813 | 0.808397 | 0.537 | 0.126802 | 0.705 | 0.340124 |
| Q9R0P5 | Destrin OS=Mus musculus GN=Dstn PE=1 SV=3 | 1.14 | 0.537247 | 0.656 | 0.290738 | 0.694 | 0.311264 |
| Q8BK84 | Dual specificity phosphatase DUPD1 OS=Mus musculus GN=Dupd1 PE=2 SV=1 | 2.208 | 0.021559 | 0.614 | 0.225352 | 0.943 | 0.924608 |
| Q9D2N4 | Dystrobrevin alpha OS=Mus musculus GN=Dtna PE=1 SV=2 | 6.362 | 6.1E-07 | 1.118 | 0.846267 | 9.009 | 6.66E-16 |
| Q62165 | Dystroglycan OS=Mus musculus GN=Dag1 PE=1 SV=4 | 0.779 | 0.725879 | 0.937 | 0.824372 | 1.203 | 0.37175 |
| P11531 | Dystrophin OS=Mus musculus GN=Dmd PE=1 SV=3 | 0.931 | 0.918862 | 1.073 | 0.922195 | 1.183 | 0.322199 |
| Q8BH64 | EH domain-containing protein 2 OS=Mus musculus GN=Ehd2 PE=1 SV=1 | 1.229 | 0.418414 | 0.772 | 0.499133 | 0.643 | 0.1937 |
| Q9DCW4 | Electron transfer flavoprotein subunit beta OS=Mus musculus GN=Etfb PE=1 SV=3 | 0.9 | 0.986081 | 1.436 | 0.435449 | 0.882 | 0.908247 |
| P58252 | Elongation factor 2 OS=Mus musculus GN=Eef2 PE=1 SV=2 | 0.748 | 0.64941 | 1.224 | 0.684858 | 1.175 | 0.334891 |
| Q6ZWX6 | Eukaryotic translation initiation factor 2 subunit 1 OS=Mus musculus GN=Eif2s1 PE=1 SV=3 | 1.163 | 0.504505 | 0.97 | 0.888914 | 0.95 | 0.896801 |
| Q9WUK2 | Eukaryotic translation initiation factor 4H OS=Mus musculus GN=Eif4h PE=1 SV=3 | 0.623 | 0.358065 | 1.457 | 0.415874 | 1.067 | 0.587078 |
| P63242 | Eukaryotic translation initiation factor 5A-1 OS=Mus musculus GN=Eif5a PE=1 SV=2 | 0.631 | 0.376377 | 1.265 | 0.628485 | 0.983 | 0.768189 |
| Q9ERK4 | Exportin-2 OS=Mus musculus GN=Cse1l PE=1 SV=1 | 1.548 | 0.163054 | 6.548 | 1.44E-05 | 0.97 | 0.85102 |
| P47757 | F-actin-capping protein subunit beta OS=Mus musculus GN=Capzb PE=1 SV=3 | 0.584 | 0.278279 | 1.03 | 0.999208 | 1.101 | 0.471817 |
| Q91V79 | Fat storage-inducing transmembrane protein 1 OS=Mus musculus GN=Fitm1 PE=1 SV=1 | 0.76 | 0.67911 | 0.572 | 0.168644 | 0.978 | 0.833683 |
| Q8CIB5 | Fermitin family homolog 2 OS=Mus musculus GN=Fermt2 PE=1 SV=1 | 1.094 | 0.60864 | 1.372 | 0.500833 | 1.022 | 0.660029 |
| P50608 | Fibromodulin OS=Mus musculus GN=Fmod PE=2 SV=1 | 0.747 | 0.648092 | 1.54 | 0.344694 | 0.725 | 0.395333 |
| Q8BTM8 | Filamin-A OS=Mus musculus GN=Flna PE=1 SV=5 | 0.885 | 0.97929 | 0.827 | 0.606965 | 0.828 | 0.72681 |
| Q80X90 | Filamin-B OS=Mus musculus GN=Flnb PE=1 SV=3 | 0.79 | 0.751837 | 0.646 | 0.273898 | 0.985 | 0.790514 |
| Q9R059 | Four and a half LIM domains protein 3 OS=Mus musculus GN=Fhl3 PE=1 SV=2 | 0.62 | 0.352851 | 0.517 | 0.105908 | 0.991 | 0.745733 |
| Q61584 | Fragile X mental retardation syndrome-related protein 1 OS=Mus musculus GN=Fxr1 PE=1 SV=2 | 0.583 | 0.277814 | 1.014 | 0.971392 | 1.172 | 0.367667 |
| P70695 | Fructose-1,6-bisphosphatase isozyme 2 OS=Mus musculus GN=Fbp2 PE=1 SV=2 | 1.284 | 0.357733 | 0.375 | 0.017977 | 1.114 | 0.532185 |
| Q5FWB7 | Fructose-bisphosphate aldolase OS=Mus musculus GN=Aldoa PE=1 SV=1 | 0.847 | 0.891155 | 1.003 | 0.950761 | 1.063 | 0.557334 |
| P16045 | Galectin-1 OS=Mus musculus GN=Lgals1 PE=1 SV=3 | 1.037 | 0.706914 | 1.595 | 0.304753 | 1.186 | 0.317831 |
| Q9EQ83 | Gamma sarcoglycan OS=Mus musculus GN=Sgcg PE=1 SV=1 | 0.629 | 0.371422 | 1.126 | 0.834316 | 0.248 | 0.000166 |
| P11352 | Glutathione peroxidase 1 OS=Mus musculus GN=Gpx1 PE=1 SV=2 | 0.863 | 0.928923 | 1.047 | 0.96788 | 0.759 | 0.601762 |
| Q9Z1E4 | Glycogen [starch] synthase, muscle OS=Mus musculus GN=Gys1 PE=1 SV=2 | 0.861 | 0.92333 | 1.085 | 0.902057 | 0.828 | 0.726599 |
| Q9WUB3 | Glycogen phosphorylase, muscle form OS=Mus musculus GN=Pygm PE=1 SV=3 | 0.918 | 0.947415 | 1.151 | 0.794316 | 0.925 | 0.947754 |
| Q9JK92 | Heat shock protein beta-8 OS=Mus musculus GN=Hspb8 PE=1 SV=1 | 0.564 | 0.240672 | 20.534 | 2.25E-12 | 1.696 | 0.147329 |
| P51859 | Hepatoma-derived growth factor OS=Mus musculus GN=Hdgf PE=1 SV=2 | 1.351 | 0.294418 | 1.411 | 0.460244 | 0.817 | 0.762131 |
| Q7TMK9 | Heterogeneous nuclear ribonucleoprotein Q OS=Mus musculus GN=Syncrip PE=1 SV=2 | 0.863 | 0.928508 | 0.881 | 0.714 | 0.821 | 0.775986 |
| P97443 | Histone-lysine N-methyltransferase Smyd1 OS=Mus musculus GN=Smyd1 PE=1 SV=3 | 0.969 | 0.838318 | 1.398 | 0.473022 | 0.909 | 0.999706 |
| Q61738 | Integrin alpha-7 OS=Mus musculus GN=Itga7 PE=1 SV=3 | 0.616 | 0.342693 | 1.101 | 0.874135 | 1.465 | 0.134071 |
| O55222 | Integrin-linked protein kinase OS=Mus musculus GN=Ilk PE=1 SV=2 | 1.109 | 0.584344 | 1.109 | 0.861618 | 0.993 | 0.753707 |
| Q8VCX5-2 | Isoform 2 of Calcium uptake protein 1, mitochondrial OS=Mus musculus GN=Micu1 | 0.313 | 0.007652 | 0.619 | 0.232665 | 0.73 | 0.624232 |
| P58281-2 | Isoform 2 of Dynamin-like 120 kDa protein, mitochondrial OS=Mus musculus GN=Opa1 | 1.496 | 0.190516 | 0.38 | 0.019309 | 0.578 | 0.31957 |
| P09055-2 | Isoform 2 of Integrin beta-1 OS=Mus musculus GN=Itgb1 | 0.948 | 0.881487 | 1.123 | 0.839181 | 0.879 | 0.898333 |
| Q62234-2 | Isoform 2 of Myomesin-1 OS=Mus musculus GN=Myom1 | 0.853 | 0.904204 | 0.945 | 0.84143 | 1.103 | 0.468397 |
| Q3TJD7-2 | Isoform 2 of PDZ and LIM domain protein 7 OS=Mus musculus GN=Pdlim7 | 0.87 | 0.945334 | 1.234 | 0.670556 | 0.946 | 0.882737 |
| Q9QYG0-2 | Isoform 2 of Protein NDRG2 OS=Mus musculus GN=Ndrg2 | 0.738 | 0.625091 | 0.852 | 0.656752 | 4.563 | 0.000159 |
| O70622-2 | Isoform 2 of Reticulon-2 OS=Mus musculus GN=Rtn2 | 0.74 | 0.629949 | 0.769 | 0.49355 | 0.433 | 0.00537 |
| Q70IV5-2 | Isoform 2 of Synemin OS=Mus musculus GN=Synm | 1.175 | 0.487037 | 2.859 | 0.016604 | 0.63 | 0.167388 |
| P58774-2 | Isoform 2 of Tropomyosin beta chain OS=Mus musculus GN=Tpm2 | 2.575 | 0.00716 | 1.651 | 0.268178 | 0.05 | 1E-17 |
| Q9JKB3-2 | Isoform 2 of Y-box-binding protein 3 OS=Mus musculus GN=Ybx3 | 0.758 | 0.674642 | 2.508 | 0.036782 | 0.806 | 0.650933 |
| P47857-3 | Isoform 3 of ATP-dependent 6-phosphofructokinase, muscle type OS=Mus musculus GN=Pfkm | 0.774 | 0.713293 | 0.961 | 0.870756 | 0.814 | 0.677029 |
| A2ASS6-3 | Isoform 3 of Titin OS=Mus musculus GN=Ttn | 6.707 | 3.02E-07 | 1.851 | 0.168847 | 0.322 | 0.000124 |
| Q9JKS4-4 | Isoform 4 of LIM domain-binding protein 3 OS=Mus musculus GN=Ldb3 | 1.186 | 0.472485 | 1.075 | 0.918556 | 0.825 | 0.713309 |
| Q9JKS4-6 | Isoform 6 of LIM domain-binding protein 3 OS=Mus musculus GN=Ldb3 | 0.744 | 0.641157 | 0.327 | 0.00717 | 0.967 | 0.818582 |
| P52480-2 | Isoform M1 of Pyruvate kinase PKM OS=Mus musculus GN=Pkm | 0.764 | 0.69043 | 1.25 | 0.648792 | 0.97 | 0.808873 |
| Q02357-7 | Isoform Mu7 of Ankyrin-1 OS=Mus musculus GN=Ank1 | 0.553 | 0.222006 | 0.466 | 0.063138 | 0.923 | 0.928738 |
| M0QW57 | Junctional sarcoplasmic reticulum protein 1 OS=Mus musculus GN=Jsrp1 PE=1 SV=1 | 0.621 | 0.3542 | 1.447 | 0.425057 | 0.363 | 0.000566 |
| Q9ET80 | Junctophilin-1 OS=Mus musculus GN=Jph1 PE=1 SV=1 | 0.976 | 0.822887 | 1.091 | 0.891649 | 0.697 | 0.318247 |
| Q9ET78 | Junctophilin-2 OS=Mus musculus GN=Jph2 PE=1 SV=2 | 0.644 | 0.404598 | 1.03 | 0.999926 | 0.936 | 0.914185 |
| A0A0R4J166 | Kelch-like protein 40 OS=Mus musculus GN=Klhl40 PE=1 SV=1 | 0.956 | 0.865892 | 1.198 | 0.721962 | 1.143 | 0.401189 |
| A2AUC9 | Kelch-like protein 41 OS=Mus musculus GN=Klhl41 PE=1 SV=1 | 0.771 | 0.706718 | 0.775 | 0.505138 | 1.101 | 0.472363 |
| Q60675 | Laminin subunit alpha-2 OS=Mus musculus GN=Lama2 PE=1 SV=2 | 0.561 | 0.23571 | 1.526 | 0.356375 | 0.849 | 0.795779 |
| P97927 | Laminin subunit alpha-4 OS=Mus musculus GN=Lama4 PE=1 SV=2 | 2.287 | 0.016946 | 3.78 | 0.002291 | 0.611 | 0.255465 |
| E9QN70 | Laminin subunit beta-1 OS=Mus musculus GN=Lamb1 PE=1 SV=1 | 1.342 | 0.302161 | 0.641 | 0.26706 | 0.738 | 0.473017 |
| Q6PB66 | Leucine-rich PPR motif-containing protein, mitochondrial OS=Mus musculus GN=Lrpprc PE=1 SV=2 | 0.379 | 0.02915 | 0.851 | 0.654486 | 0.932 | 0.900224 |
| G3UW82 | MCG140437, isoform CRA_d OS=Mus musculus GN=Myh2 PE=1 SV=1 | 0.856 | 0.911339 | 0.86 | 0.672258 | 0.869 | 0.864807 |
| A0A509 | MCG4625 OS=Mus musculus GN=Myot PE=1 SV=1 | 0.845 | 0.886687 | 1.246 | 0.654132 | 0.973 | 0.799267 |
| O08911 | Mitogen-activated protein kinase 12 OS=Mus musculus GN=Mapk12 PE=1 SV=1 | 0.634 | 0.382693 | 0.827 | 0.6077 | 1.203 | 0.397238 |
| P47811 | Mitogen-activated protein kinase 14 OS=Mus musculus GN=Mapk14 PE=1 SV=3 | 1.229 | 0.419338 | 1.196 | 0.725602 | 0.623 | 0.343605 |
| Q3UDP9 | Monocarboxylate transporter 4 OS=Mus musculus GN=Slc16a3 PE=1 SV=1 | 0.855 | 0.908276 | 1.331 | 0.54753 | 0.878 | 0.957597 |
| A2AMM0 | Muscle-related coiled-coil protein OS=Mus musculus GN=Murc PE=1 SV=1 | 0.81 | 0.802609 | 0.621 | 0.236463 | 1.398 | 0.106361 |
| O70624 | Myocilin OS=Mus musculus GN=Myoc PE=1 SV=1 | 1.059 | 0.66759 | 1.17 | 0.763563 | 1.349 | 0.138534 |
| P04247 | Myoglobin OS=Mus musculus GN=Mb PE=1 SV=3 | 0.538 | 0.195729 | 0.465 | 0.06247 | 0.683 | 0.282176 |
| Q14BI5 | Myomesin 2 OS=Mus musculus GN=Myom2 PE=1 SV=1 | 0.888 | 0.985494 | 1.228 | 0.679865 | 0.855 | 0.817722 |
| A2ABU4 | Myomesin-3 OS=Mus musculus GN=Myom3 PE=1 SV=1 | 1.188 | 0.470536 | 1.676 | 0.253245 | 1.05 | 0.588091 |
| Q5DTJ9 | Myopalladin OS=Mus musculus GN=Mypn PE=1 SV=2 | 1.44 | 0.225445 | 0.907 | 0.765919 | 0.554 | 0.105597 |
| P05977 | Myosin light chain 1/3, skeletal muscle isoform OS=Mus musculus GN=Myl1 PE=1 SV=2 | 0.743 | 0.638576 | 1.131 | 0.825144 | 0.476 | 0.015054 |
| P09542 | Myosin light chain 3 OS=Mus musculus GN=Myl3 PE=1 SV=4 | 1.483 | 0.198347 | 0.574 | 0.170952 | 0.709 | 0.349415 |
| P09541 | Myosin light chain 4 OS=Mus musculus GN=Myl4 PE=1 SV=3 | 0.576 | 0.262852 | 1.801 | 0.189855 | 0.67 | 0.390966 |
| Q8CI43 | Myosin light chain 6B OS=Mus musculus GN=Myl6b PE=1 SV=1 | 2.08 | 0.031885 | 0.954 | 0.85727 | 0.671 | 0.254213 |
| Q8VCR8 | Myosin light chain kinase 2, skeletal/cardiac muscle OS=Mus musculus GN=Mylk2 PE=1 SV=2 | 1.089 | 0.615602 | 1.027 | 0.996012 | 0.235 | 3.76E-07 |
| B1B1A8 | Myosin light chain kinase, smooth muscle OS=Mus musculus GN=Mylk PE=1 SV=1 | 0.631 | 0.375015 | 0.665 | 0.304581 | 0.399 | 0.066499 |
| Q60605 | Myosin light polypeptide 6 OS=Mus musculus GN=Myl6 PE=1 SV=3 | 0.452 | 0.0833 | 0.392 | 0.023667 | 0.736 | 0.67874 |
| Q3THE2 | Myosin regulatory light chain 12B OS=Mus musculus GN=Myl12b PE=1 SV=2 | 1.008 | 0.761096 | 0.916 | 0.783802 | 1.648 | 0.06717 |
| A0A0U1RP93 | Myosin regulatory light chain 2, skeletal muscle isoform OS=Mus musculus GN=Mylpf PE=1 SV=1 | 11.608 | 7.17E-11 | 1.086 | 0.900403 | 1.498 | 0.309616 |
| P97457 | Myosin regulatory light chain 2, skeletal muscle isoform OS=Mus musculus GN=Mylpf PE=1 SV=3 | 1.292 | 0.349332 | 0.621 | 0.236442 | 0.78 | 0.565643 |
| P51667 | Myosin regulatory light chain 2, ventricular/cardiac muscle isoform OS=Mus musculus GN=Myl2 PE=1 SV=3 | 1.205 | 0.44834 | 0.673 | 0.318576 | 0.636 | 0.178718 |
| Q9CQ19 | Myosin regulatory light polypeptide 9 OS=Mus musculus GN=Myl9 PE=1 SV=3 |  |  |  |  |  |  |
| Q5SX40 | Myosin-1 OS=Mus musculus GN=Myh1 PE=1 SV=1 | 1.196 | 0.459372 | 0.877 | 0.706398 | 0.96 | 0.83912 |
| E9QPE7 | Myosin-11 OS=Mus musculus GN=Myh11 PE=1 SV=1 | 0.773 | 0.710614 | 0.754 | 0.465615 | 0.711 | 0.356434 |
| P13541 | Myosin-3 OS=Mus musculus GN=Myh3 PE=2 SV=2 | 2.804 | 0.003666 | 0.568 | 0.162817 | 1.329 | 0.154347 |
| Q5SX39 | Myosin-4 OS=Mus musculus GN=Myh4 PE=2 SV=1 | 0.895 | 0.998342 | 1.248 | 0.652131 | 0.952 | 0.862818 |
| Q91Z83 | Myosin-7 OS=Mus musculus GN=Myh7 PE=1 SV=1 | 0.901 | 0.98492 | 1.421 | 0.450021 | 0.695 | 0.312908 |
| P13542 | Myosin-8 OS=Mus musculus GN=Myh8 PE=2 SV=2 | 1.02 | 0.738422 | 1.293 | 0.593625 | 1.258 | 0.22227 |
| Q8VDD5 | Myosin-9 OS=Mus musculus GN=Myh9 PE=1 SV=4 | 1.204 | 0.449446 | 0.88 | 0.711957 | 0.887 | 0.926469 |
| Q5XKE0 | Myosin-binding protein C, fast-type OS=Mus musculus GN=Mybpc2 PE=1 SV=1 | 1.058 | 0.669453 | 1.548 | 0.339001 | 1.22 | 0.269172 |
| P70402 | Myosin-binding protein H OS=Mus musculus GN=Mybph PE=2 SV=2 | 0.841 | 0.876685 | 1.237 | 0.66729 | 1.214 | 0.278273 |
| Q9JK37 | Myozenin-1 OS=Mus musculus GN=Myoz1 PE=1 SV=1 | 0.611 | 0.332507 | 1.136 | 0.817414 | 0.946 | 0.880884 |
| Q9JJW5 | Myozenin-2 OS=Mus musculus GN=Myoz2 PE=1 SV=1 | 0.475 | 0.108294 | 1.82 | 0.181605 | 0.655 | 0.219039 |
| Q9CQ75 | NADH dehydrogenase [ubiquinone] 1 alpha subcomplex subunit 2 OS=Mus musculus GN=Ndufa2 PE=1 SV=3 | 1.143 | 0.532011 | 9.921 | 1.08E-07 | 0.413 | 0.126833 |
| P70670 | Nascent polypeptide-associated complex subunit alpha, muscle-specific form OS=Mus musculus GN=Naca PE=1 SV=2 | 1.412 | 0.245686 | 0.907 | 0.76577 | 0.924 | 0.952044 |
| Q80XB4 | Nebulin-related-anchoring protein OS=Mus musculus GN=Nrap PE=1 SV=3 | 1.236 | 0.410079 | 0.605 | 0.213088 | 0.945 | 0.887446 |
| Q545M7 | Parvalbumin alpha OS=Mus musculus GN=Pvalb PE=1 SV=1 | 0.949 | 0.880087 | 1.422 | 0.449035 | 0.865 | 0.851724 |
| O70400 | PDZ and LIM domain protein 1 OS=Mus musculus GN=Pdlim1 PE=1 SV=4 | 0.507 | 0.149361 | 0.787 | 0.529258 | 0.73 | 0.410087 |
| O70209 | PDZ and LIM domain protein 3 OS=Mus musculus GN=Pdlim3 PE=1 SV=1 | 0.989 | 0.797533 | 1.154 | 0.788386 | 0.878 | 0.895852 |
| O88492 | Perilipin-4 OS=Mus musculus GN=Plin4 PE=1 SV=2 | 0.597 | 0.30505 | 0.551 | 0.142872 | 1.145 | 0.385817 |
| Q61171 | Peroxiredoxin-2 OS=Mus musculus GN=Prdx2 PE=1 SV=3 | 0.988 | 0.800116 | 1.131 | 0.826167 | 0.803 | 0.642197 |
| P09411 | Phosphoglycerate kinase 1 OS=Mus musculus GN=Pgk1 PE=1 SV=4 | 0.959 | 0.858323 | 1.386 | 0.485665 | 1.129 | 0.415871 |
| P07934 | Phosphorylase b kinase gamma catalytic chain, skeletal muscle/heart isoform OS=Mus musculus GN=Phkg1 PE=1 SV=3 | 0.706 | 0.547943 | 0.809 | 0.571449 | 1.247 | 0.324528 |
| Q9QXS1 | Plectin OS=Mus musculus GN=Plec PE=1 SV=3 | 0.582 | 0.274411 | 1.175 | 0.756481 | 1.184 | 0.32104 |
| O54724 | Polymerase I and transcript release factor OS=Mus musculus GN=Ptrf PE=1 SV=1 | 0.825 | 0.836665 | 1.401 | 0.470306 | 0.679 | 0.272428 |
| P48678 | Prelamin-A/C OS=Mus musculus GN=Lmna PE=1 SV=2 | 0.721 | 0.584071 | 1.721 | 0.228417 | 0.825 | 0.715992 |
| Q543S0 | Prolargin OS=Mus musculus GN=Prelp PE=1 SV=1 | 0.738 | 0.625775 | 0.891 | 0.733903 | 0.874 | 0.883518 |
| Q9R1P4 | Proteasome subunit alpha type-1 OS=Mus musculus GN=Psma1 PE=1 SV=1 | 1.411 | 0.246191 | 1.547 | 0.339446 | 0.624 | 0.247324 |
| Q9QUM9 | Proteasome subunit alpha type-6 OS=Mus musculus GN=Psma6 PE=1 SV=1 | 0.624 | 0.359672 | 0.81 | 0.572884 | 1.116 | 0.447209 |
| E9QA15 | Protein Cald1 OS=Mus musculus GN=Cald1 PE=1 SV=1 | 1.165 | 0.501403 | 0.681 | 0.331884 | 1.233 | 0.497731 |
| D3Z7H8 | Protein Cilp2 OS=Mus musculus GN=Cilp2 PE=1 SV=1 | 1.645 | 0.121353 | 1.659 | 0.263243 | 0.458 | 0.009992 |
| Q99JB8 | Protein kinase C and casein kinase II substrate protein 3 OS=Mus musculus GN=Pacsin3 PE=1 SV=1 | 0.838 | 0.86828 | 0.638 | 0.261059 | 1.453 | 0.078385 |
| F6RQD1 | Protein Mybpc1 (Fragment) OS=Mus musculus GN=Mybpc1 PE=1 SV=1 | 1.06 | 0.664748 | 1.214 | 0.699251 | 0.705 | 0.339512 |
| Q6P6L5 | Protein Mybpc1 OS=Mus musculus GN=Mybpc1 PE=1 SV=1 | 0.912 | 0.959485 | 1.26 | 0.635851 | 0.991 | 0.747826 |
| D3YU50 | Protein Mybpc1 OS=Mus musculus GN=Mybpc1 PE=1 SV=1 | 1.449 | 0.219628 | 0.814 | 0.58112 | 1.139 | 0.397874 |
| Q9QYG0 | Protein NDRG2 OS=Mus musculus GN=Ndrg2 PE=1 SV=1 | 0.946 | 0.885702 | 0.884 | 0.72037 | 1.128 | 0.417493 |
| Q99MR9 | Protein phosphatase 1 regulatory subunit 3A OS=Mus musculus GN=Ppp1r3a PE=1 SV=2 | 0.542 | 0.203312 | 0.633 | 0.25352 | 0.686 | 0.289177 |
| Q8CGY6 | Protein unc-45 homolog B OS=Mus musculus GN=Unc45b PE=1 SV=1 | 1.205 | 0.448626 | 1.351 | 0.524526 | 0.813 | 0.797984 |
| P35486 | Pyruvate dehydrogenase E1 component subunit alpha, somatic form, mitochondrial OS=Mus musculus GN=Pdha1 PE=1 SV=1 | 0.664 | 0.449817 | 0.95 | 0.850399 | 1.12 | 0.433478 |
| P52480 | Pyruvate kinase PKM OS=Mus musculus GN=Pkm PE=1 SV=4 | 0.58 | 0.271324 | 1.377 | 0.495072 | 0.89 | 0.998599 |
| P50396 | Rab GDP dissociation inhibitor alpha OS=Mus musculus GN=Gdi1 PE=1 SV=3 | 0.929 | 0.923632 | 0.774 | 0.502665 | 1.099 | 0.496998 |
| Q4VAE6 | Ras family member A OS=Mus musculus GN=Rhoa PE=1 SV=1 | 1.096 | 0.604107 | 1.25 | 0.648648 | 0.77 | 0.73661 |
| P68040 | Receptor of activated protein C kinase 1 OS=Mus musculus GN=Rack1 PE=1 SV=3 | 0.642 | 0.398683 | 1.603 | 0.299391 | 1.157 | 0.364612 |
| E9PZQ0 | Ryanodine receptor 1 OS=Mus musculus GN=Ryr1 PE=1 SV=1 | 1.011 | 0.755184 | 0.867 | 0.687793 | 1.022 | 0.659398 |
| Q7TQ48 | Sarcalumenin OS=Mus musculus GN=Srl PE=1 SV=1 | 0.912 | 0.960879 | 0.837 | 0.627138 | 0.928 | 0.939672 |
| Q8R429 | Sarcoplasmic/endoplasmic reticulum calcium ATPase 1 OS=Mus musculus GN=Atp2a1 PE=1 SV=1 | 0.897 | 0.994324 | 1.167 | 0.769079 | 0.815 | 0.680293 |
| O55143 | Sarcoplasmic/endoplasmic reticulum calcium ATPase 2 OS=Mus musculus GN=Atp2a2 PE=1 SV=2 | 0.502 | 0.142084 | 0.826 | 0.605916 | 2.502 | 0.002317 |
| P63328 | Serine/threonine-protein phosphatase 2B catalytic subunit alpha isoform OS=Mus musculus GN=Ppp3ca PE=1 SV=1 | 1.121 | 0.565456 | 0.487 | 0.07897 | 1.435 | 0.13152 |
| P62137 | Serine/threonine-protein phosphatase PP1-alpha catalytic subunit OS=Mus musculus GN=Ppp1ca PE=1 SV=1 | 0.207 | 0.000203 | 4.206 | 0.000965 | 0.802 | 0.812818 |
| P62141 | Serine/threonine-protein phosphatase PP1-beta catalytic subunit OS=Mus musculus GN=Ppp1cb PE=1 SV=3 | 0.753 | 0.662837 | 1.177 | 0.754226 | 0.488 | 0.047284 |
| Q8BZ71 | SH3 and cysteine-rich domain-containing protein 3 OS=Mus musculus GN=Stac3 PE=2 SV=1 | 0.684 | 0.496499 | 0.794 | 0.542458 | 0.726 | 0.397167 |
| Q99LM3 | Smoothelin-like protein 1 OS=Mus musculus GN=Smtnl1 PE=1 SV=1 | 0.938 | 0.90245 | 0.514 | 0.103612 | 0.386 | 0.001277 |
| Q8BJS4 | SUN domain-containing protein 2 OS=Mus musculus GN=Sun2 PE=1 SV=3 | 1.691 | 0.105107 | 0.707 | 0.37862 | 0.899 | 0.996907 |
| P60712 | SWISS-PROT:P60712 (Bos taurus) Actin, cytoplasmic 1 | 0.786 | 0.743829 | 0.686 | 0.341395 | 0.938 | 0.906243 |
| Q91YE8 | Synaptopodin-2 OS=Mus musculus GN=Synpo2 PE=1 SV=2 | 0.875 | 0.957019 | 1.233 | 0.671785 | 1.036 | 0.624714 |
| P43025 | Tetranectin OS=Mus musculus GN=Clec3b PE=1 SV=2 | 0.921 | 0.940662 | 100 | 1E-17 | 1.173 | 0.430889 |
| O08583 | THO complex subunit 4 OS=Mus musculus GN=Alyref PE=1 SV=3 | 3.514 | 0.000502 | 1.789 | 0.19499 | 1.218 | 0.322463 |
| A2ASS6 | Titin OS=Mus musculus GN=Ttn PE=1 SV=1 | 0.92 | 0.941625 | 1.172 | 0.76117 | 0.967 | 0.81616 |
| P82198 | Transforming growth factor-beta-induced protein ig-h3 OS=Mus musculus GN=Tgfbi PE=1 SV=1 | 0.914 | 0.956171 | 0.651 | 0.282058 | 0.792 | 0.605689 |
| E9Q9K5 | Triadin OS=Mus musculus GN=Trdn PE=1 SV=1 | 1.169 | 0.496116 | 1.08 | 0.909909 | 0.753 | 0.477832 |
| Q3TMP8 | Trimeric intracellular cation channel type A OS=Mus musculus GN=Tmem38a PE=1 SV=2 | 0.83 | 0.849994 | 0.738 | 0.434435 | 1.028 | 0.64472 |
| Q1XH17 | Tripartite motif-containing protein 72 OS=Mus musculus GN=Trim72 PE=1 SV=1 | 0.91 | 0.965097 | 0.865 | 0.683915 | 0.899 | 0.967152 |
| Q3UN19 | Tropomodulin 4 OS=Mus musculus GN=Tmod4 PE=1 SV=1 | 1.371 | 0.277129 | 0.605 | 0.212204 | 1.109 | 0.462063 |
| P49813 | Tropomodulin-1 OS=Mus musculus GN=Tmod1 PE=1 SV=2 | 0.621 | 0.354958 | 1.072 | 0.925282 | 8.196 | 2.22E-16 |
| P58771 | Tropomyosin alpha-1 chain OS=Mus musculus GN=Tpm1 PE=1 SV=1 | 1.442 | 0.22464 | 1.197 | 0.724183 | 0.98 | 0.778455 |
| P21107 | Tropomyosin alpha-3 chain OS=Mus musculus GN=Tpm3 PE=1 SV=3 | 0.563 | 0.24046 | 1.188 | 0.736984 | 0.759 | 0.497437 |
| P58774 | Tropomyosin beta chain OS=Mus musculus GN=Tpm2 PE=1 SV=1 | 1.08 | 0.630764 | 1.136 | 0.817702 | 0.766 | 0.519369 |
| P20801 | Troponin C, skeletal muscle OS=Mus musculus GN=Tnnc2 PE=1 SV=2 | 1.328 | 0.314982 | 0.633 | 0.25415 | 0.698 | 0.320444 |
| P19123 | Troponin C, slow skeletal and cardiac muscles OS=Mus musculus GN=Tnnc1 PE=1 SV=1 | 0.969 | 0.83707 | 0.373 | 0.017223 | 0.713 | 0.364363 |
| A2A6J8 | Troponin I, fast skeletal muscle (Fragment) OS=Mus musculus GN=Tnni2 PE=1 SV=1 | 0.626 | 0.364886 | 1.125 | 0.835005 | 1.081 | 0.516156 |
| Q9WUZ5 | Troponin I, slow skeletal muscle OS=Mus musculus GN=Tnni1 PE=1 SV=3 | 0.677 | 0.478716 | 0.688 | 0.34463 | 1.339 | 0.145724 |
| A0A0R4J1B0 | Troponin T, fast skeletal muscle OS=Mus musculus GN=Tnnt3 PE=1 SV=1 | 1.009 | 0.75884 | 0.918 | 0.788283 | 1.076 | 0.526282 |
| Z4YNB2 | Troponin T, fast skeletal muscle OS=Mus musculus GN=Tnnt3 PE=1 SV=1 | 1.015 | 0.747729 | 1.16 | 0.779919 | 0.938 | 0.905341 |
| O88346 | Troponin T, slow skeletal muscle OS=Mus musculus GN=Tnnt1 PE=2 SV=3 | 0.77 | 0.704538 | 1.181 | 0.746868 | 0.8 | 0.629247 |
| P68369 | Tubulin alpha-1A chain OS=Mus musculus GN=Tuba1a PE=1 SV=1 | 0.79 | 0.753203 | 0.985 | 0.917771 | 0.666 | 0.24286 |
| Q9JJZ2 | Tubulin alpha-8 chain OS=Mus musculus GN=Tuba8 PE=1 SV=1 | 0.414 | 0.050536 | 1.154 | 0.789474 | 0.794 | 0.61711 |
| Q9WTI7 | Unconventional myosin-Ic OS=Mus musculus GN=Myo1c PE=1 SV=2 | 1.115 | 0.574614 | 1.068 | 0.931502 | 0.996 | 0.73196 |
| Q78IK2 | Up-regulated during skeletal muscle growth protein 5 OS=Mus musculus GN=Usmg5 PE=1 SV=1 | 0.226 | 0.000481 | 0.533 | 0.123107 | 0.258 | 0.003452 |
| Q9QY80 | Very-long-chain (3R)-3-hydroxyacyl-CoA dehydratase 1 OS=Mus musculus GN=Hacd1 PE=2 SV=1 | 0.144 | 3.54E-06 | 1.78 | 0.199115 | 0.76 | 0.717077 |
| Q60932 | Voltage-dependent anion-selective channel protein 1 OS=Mus musculus GN=Vdac1 PE=1 SV=3 | 0.915 | 0.952916 | 0.786 | 0.526491 | 1.111 | 0.450893 |
| Q60930 | Voltage-dependent anion-selective channel protein 2 OS=Mus musculus GN=Vdac2 PE=1 SV=2 | 0.863 | 0.929122 | 1.071 | 0.926378 | 0.879 | 0.898584 |
| Q4KL26 | Voltage-dependent calcium channel gamma subunit OS=Mus musculus GN=Cacng1 PE=2 SV=1 | 0.401 | 0.041579 | 0.784 | 0.522447 | 2.9 | 0.006047 |
| O08532 | Voltage-dependent calcium channel subunit alpha-2/delta-1 OS=Mus musculus GN=Cacna2d1 PE=1 SV=1 | 0.811 | 0.804546 | 0.873 | 0.698636 | 1.115 | 0.443733 |
| Q02789 | Voltage-dependent L-type calcium channel subunit alpha-1S OS=Mus musculus GN=Cacna1s PE=1 SV=2 | 0.754 | 0.664366 | 0.68 | 0.330007 | 0.946 | 0.8809 |
| Q4U4S6 | Xin actin-binding repeat-containing protein 2 OS=Mus musculus GN=Xirp2 PE=1 SV=1 | 1.248 | 0.396682 | 1.363 | 0.511069 | 0.697 | 0.45843 |
