# Supplementary material for: Defects in sarcolemma repair and skeletal muscle function after injury in a mouse model of Niemann-Pick type A/B disease
Source: Skelet Muscle. 2019 Jan 5;9:1. doi: 10.1186/s13395-018-0187-5 (PMC6320626; doi:10.1186/s13395-018-0187-5)
Supplement: Supplementary file 6 — Table S5. Master proteins from a skeletal muscle subset significantly up- or downregulated in QF, FDB, or TP. (DOCX 23 kb) [file 13395_2018_187_MOESM6_ESM.docx]

**Table S5. Master Proteins From Skeletal Muscle Subset Significantly Up or Downregulated in QF, FDB or TP.**

Green, master proteins downregulated in ASM^-/-^ relative to WT.

Pink, master proteins upregulated in ASM^-/-^ relative to WT.

**QF**

| **Uniprot Accession #** | **Description** | **Abundance Ratio: (Quad, KO) / (Quad, WT)** | **Abundance Ratio P-Value: (Quad, KO)/ (Quad, WT)** |
| --- | --- | --- | --- |
| Q9QY80 | Very-long-chain (3R)-3-hydroxyacyl-CoA dehydratase 1 OS=Mus musculus GN=Hacd1 PE=2 SV=1 | 0.144 | 0.000 |
| P62137 | Serine/threonine-protein phosphatase PP1-alpha catalytic subunit OS=Mus musculus GN=Ppp1ca PE=1 SV=1 | 0.207 | 0.000 |
| Q78IK2 | Up-regulated during skeletal muscle growth protein 5 OS=Mus musculus GN=Usmg5 PE=1 SV=1 | 0.226 | 0.000 |
| O09161 | Calsequestrin-2 OS=Mus musculus GN=Casq2 PE=1 SV=3 | 0.243 | 0.001 |
| Q8VCX5-2 | Isoform 2 of Calcium uptake protein 1, mitochondrial OS=Mus musculus GN=Micu1 | 0.313 | 0.008 |
| P50462 | Cysteine and glycine-rich protein 3 OS=Mus musculus GN=Csrp3 PE=1 SV=1 | 0.334 | 0.012 |
| Q6PB66 | Leucine-rich PPR motif-containing protein, mitochondrial OS=Mus musculus GN=Lrpprc PE=1 SV=2 | 0.379 | 0.029 |
| Q4KL26 | Voltage-dependent calcium channel gamma subunit OS=Mus musculus GN=Cacng1 PE=2 SV=1 | 0.401 | 0.042 |
| Q8CI43 | Myosin light chain 6B OS=Mus musculus GN=Myl6b PE=1 SV=1 | 2.08 | 0.032 |
| Q8BK84 | Dual specificity phosphatase DUPD1 OS=Mus musculus GN=Dupd1 PE=2 SV=1 | 2.208 | 0.022 |
| P97927 | Laminin subunit alpha-4 OS=Mus musculus GN=Lama4 PE=1 SV=2 | 2.287 | 0.017 |
| P58774-2 | Isoform 2 of Tropomyosin beta chain OS=Mus musculus GN=Tpm2 | 2.575 | 0.007 |
| P13541 | Myosin-3 OS=Mus musculus GN=Myh3 PE=2 SV=2 | 2.804 | 0.004 |
| Q04447 | Creatine kinase B-type OS=Mus musculus GN=Ckb PE=1 SV=1 | 2.954 | 0.002 |
| Q564E8 | 60S ribosomal protein L4 OS=Mus musculus GN=Rpl4 PE=1 SV=1 | 3.494 | 0.001 |
| P51125 | Calpastatin OS=Mus musculus GN=Cast PE=1 SV=2 | 3.509 | 0.001 |
| O08583 | THO complex subunit 4 OS=Mus musculus GN=Alyref PE=1 SV=3 | 3.514 | 0.001 |
| Q9D2N4 | Dystrobrevin alpha OS=Mus musculus GN=Dtna PE=1 SV=2 | 6.362 | 0.000 |
| A2ASS6-3 | Isoform 3 of Titin OS=Mus musculus GN=Ttn | 6.707 | 0.000 |
| A0A0U1RP93 | Myosin regulatory light chain 2, skeletal muscle isoform OS=Mus musculus GN=Mylpf PE=1 SV=1 | 11.608 | 0.000 |

**FDB**

| **Accession** | **Description** | **Abundance Ratio: (FDB, KO) / (FDB, WT)** | **Abundance Ratio P-Value: (FDB, KO) / (FDB, WT)** |
| --- | --- | --- | --- |
| P58774-2 | Isoform 2 of Tropomyosin beta chain OS=Mus musculus GN=Tpm2 | 0.05 | 0.000 |
| Q8VCR8 | Myosin light chain kinase 2, skeletal/cardiac muscle OS=Mus musculus GN=Mylk2 PE=1 SV=2 | 0.235 | 0.000 |
| Q9EQ83 | Gamma sarcoglycan OS=Mus musculus GN=Sgcg PE=1 SV=1 | 0.248 | 0.000 |
| Q78IK2 | Up-regulated during skeletal muscle growth protein 5 OS=Mus musculus GN=Usmg5 PE=1 SV=1 | 0.258 | 0.003 |
| A2ASS6-3 | Isoform 3 of Titin OS=Mus musculus GN=Ttn | 0.322 | 0.000 |
| P17426 | AP-2 complex subunit alpha-1 OS=Mus musculus GN=Ap2a1 PE=1 SV=1 | 0.35 | 0.008 |
| M0QW57 | Junctional sarcoplasmic reticulum protein 1 OS=Mus musculus GN=Jsrp1 PE=1 SV=1 | 0.363 | 0.001 |
| Q99LM3 | Smoothelin-like protein 1 OS=Mus musculus GN=Smtnl1 PE=1 SV=1 | 0.386 | 0.001 |
| O70622-2 | Isoform 2 of Reticulon-2 OS=Mus musculus GN=Rtn2 | 0.433 | 0.005 |
| D3Z7H8 | Protein Cilp2 OS=Mus musculus GN=Cilp2 PE=1 SV=1 | 0.458 | 0.010 |
| P05977 | Myosin light chain 1/3, skeletal muscle isoform OS=Mus musculus GN=Myl1 PE=1 SV=2 | 0.476 | 0.015 |
| O09161 | Calsequestrin-2 OS=Mus musculus GN=Casq2 PE=1 SV=3 | 0.478 | 0.026 |
| P62141 | Serine/threonine-protein phosphatase PP1-beta catalytic subunit OS=Mus musculus GN=Ppp1cb PE=1 SV=3 | 0.488 | 0.047 |
| P10107 | Annexin A1 OS=Mus musculus GN=Anxa1 PE=1 SV=2 | 0.496 | 0.023 |
| O55143 | Sarcoplasmic/endoplasmic reticulum calcium ATPase 2 OS=Mus musculus GN=Atp2a2 PE=1 SV=2 | 2.502 | 0.002 |
| Q4KL26 | Voltage-dependent calcium channel gamma subunit OS=Mus musculus GN=Cacng1 PE=2 SV=1 | 2.9 | 0.006 |
| Q9QYG0-2 | Isoform 2 of Protein NDRG2 OS=Mus musculus GN=Ndrg2 | 4.563 | 0.000 |
| P49813 | Tropomodulin-1 OS=Mus musculus GN=Tmod1 PE=1 SV=2 | 8.196 | 0.000 |
| Q9D2N4 | Dystrobrevin alpha OS=Mus musculus GN=Dtna PE=1 SV=2 | 9.009 | 0.000 |

**TP**

| **Accession** | **Description** | **Abundance Ratio: (TP, KO) / (TP, WT)** | **Abundance Ratio P-Value: (TP, KO) / (TP, WT)** |
| --- | --- | --- | --- |
| O09161 | Calsequestrin-2 OS=Mus musculus GN=Casq2 PE=1 SV=3 | 0.101 | 0.000 |
| Q9JKS4-6 | Isoform 6 of LIM domain-binding protein 3 OS=Mus musculus GN=Ldb3 | 0.327 | 0.007 |
| Q91WS0 | CDGSH iron-sulfur domain-containing protein 1 OS=Mus musculus GN=Cisd1 PE=1 SV=1 | 0.358 | 0.013 |
| P19123 | Troponin C, slow skeletal and cardiac muscles OS=Mus musculus GN=Tnnc1 PE=1 SV=1 | 0.373 | 0.017 |
| P70695 | Fructose-1,6-bisphosphatase isozyme 2 OS=Mus musculus GN=Fbp2 PE=1 SV=2 | 0.375 | 0.018 |
| P58281-2 | Isoform 2 of Dynamin-like 120 kDa protein, mitochondrial OS=Mus musculus GN=Opa1 | 0.38 | 0.019 |
| Q60605 | Myosin light polypeptide 6 OS=Mus musculus GN=Myl6 PE=1 SV=3 | 0.392 | 0.024 |
| Q9JKB3-2 | Isoform 2 of Y-box-binding protein 3 OS=Mus musculus GN=Ybx3 | 2.508 | 0.037 |
| E9QK41 | Actin-binding LIM protein 1 OS=Mus musculus GN=Ablim1 PE=1 SV=1 | 2.61 | 0.029 |
| Q70IV5-2 | Isoform 2 of Synemin OS=Mus musculus GN=Synm | 2.859 | 0.017 |
| P97927 | Laminin subunit alpha-4 OS=Mus musculus GN=Lama4 PE=1 SV=2 | 3.78 | 0.002 |
| P62137 | Serine/threonine-protein phosphatase PP1-alpha catalytic subunit OS=Mus musculus GN=Ppp1ca PE=1 SV=1 | 4.206 | 0.001 |
| Q9ERK4 | Exportin-2 OS=Mus musculus GN=Cse1l PE=1 SV=1 | 6.548 | 0.000 |
| Q9CQ75 | NADH dehydrogenase [ubiquinone] 1 alpha subcomplex subunit 2 OS=Mus musculus GN=Ndufa2 PE=1 SV=3 | 9.921 | 0.000 |
| Q9JK92 | Heat shock protein beta-8 OS=Mus musculus GN=Hspb8 PE=1 SV=1 | 20.534 | 0.000 |
| P43025 | Tetranectin OS=Mus musculus GN=Clec3b PE=1 SV=2 | 100 | 0.000 |
